# Stereogenic-at-iron mesoionic carbene complex for enantioselective C–H amidation†

**DOI:** 10.1039/d4sc03504f

**Published:** 2024-09-10

**Authors:** Nemrud Demirel, Mahiob Dawor, Greta Nadler, Sergei I. Ivlev, Eric Meggers

**Affiliations:** a Fachbereich Chemie, Philipps-Universität Marburg Hans-Meerwein-Strasse 4 35043 Marburg Germany meggers@chemie.uni-marburg.de

## Abstract

Electronically tuned *C*_2_-symmetric stereogenic-at-iron complexes, featuring strongly σ-donating 1,2,3-triazolin-5-ylidene mesoionic carbene (MIC) ligands, exhibit enhanced catalytic efficiency compared to conventional imidazol-2-ylidene analogs, as demonstrated in nitrene-mediated ring-closing C(sp^3^)–H amidation reactions. Furthermore, a chiral pinene-derived pyridyl triazole ligand enables a highly diastereoselective synthesis of a non-racemic chiral iron catalyst, thereby controlling the absolute configuration at the metal center, as confirmed by NMR and X-ray crystallography. This pinene-modified stereogenic-at-iron MIC complex demonstrates high catalytic activity and a respectable asymmetric induction in the ring-closing C(sp^3^)–H amination of *N*-benzoyloxyurea, yielding 2-imidazolidinones with enantiomeric ratios of up to 92 : 8. These findings reflect the profound potential of this new class of mesoionic carbene iron complexes in further understanding and tuning the reactivity of iron-based catalysts.

## Abstract 2

Stereogenic-at-iron complexes, featuring strongly σ-donating mesoionic carbene (MIC) ligands, exhibit enhanced catalytic efficiency compared to their normal N-heterocyclic carbene congeners in nitrene-mediated ring-closing C(sp^3^)–H amidations.

## Graphical abstract

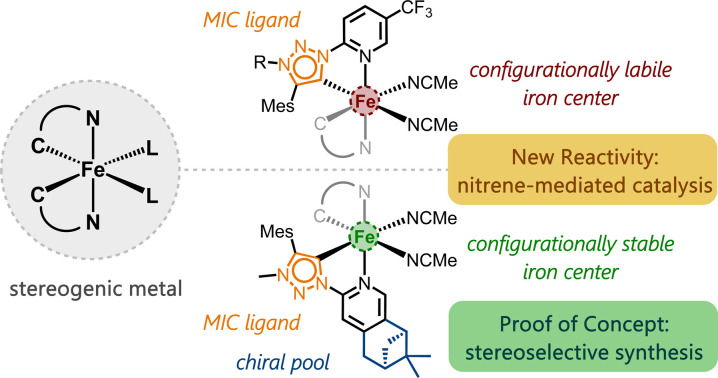

## Introduction

Iron-catalyzed reactions have recently gained significant attention in both academia and industry, driven by the increasing demand for nontoxic, sustainable, and cost-effective alternatives to commonly used precious metals.^1–3^ Given iron's versatility and the capabilities of its complexes in redox and radical chemistry, coupled with its high abundance in the Earth's crust, low toxicity, and its natural role as a catalytic metal, it is no surprise that iron emerges as a particularly promising candidate to fulfill these criteria. For example, the field of asymmetric iron catalysis has seen notable progress over the last two decades, resulting in a diverse array of chiral iron catalysts.^4^ However, for iron to rival its precious metal counterparts, numerous challenges persist, including improving catalytic performance, discovering new and unique catalytic processes, identifying new chiral iron catalyst scaffolds, and establishing economic syntheses for these chiral iron catalysts.

**Fig. 1:**
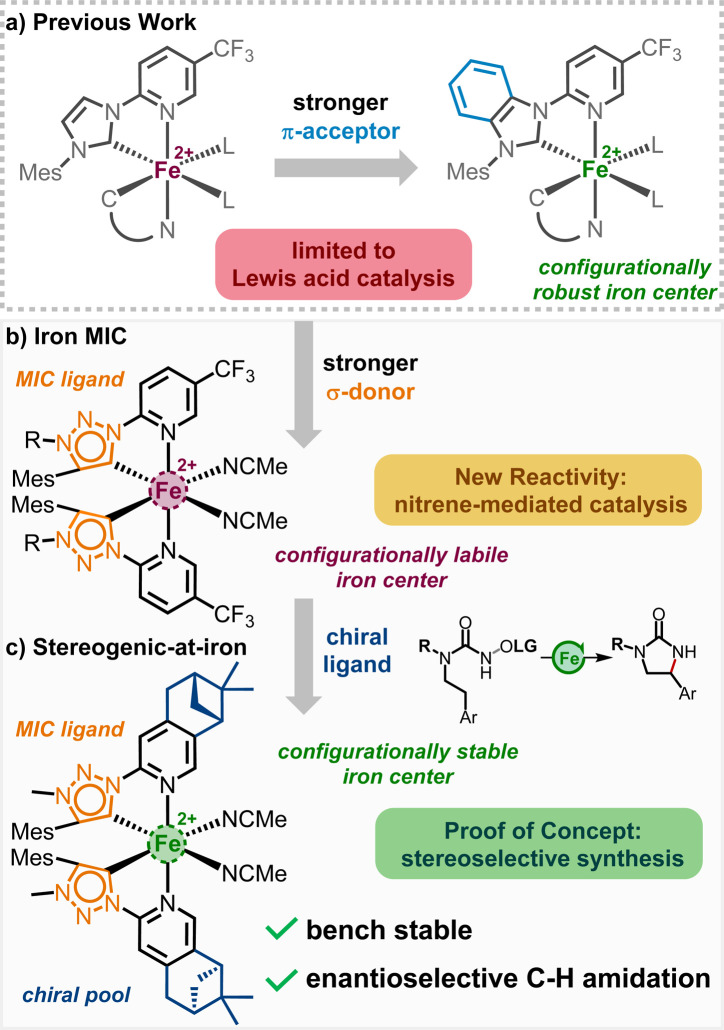
Stereogenic-at-iron N-heterocyclic carbene complexes: previous work and this study.

With respect to the development of novel iron catalyst scaffolds, our group recently reported the first examples of chiral iron catalysts in which the overall chirality is exclusively due to a stereogenic iron center (Fig. 1a).^5^ This is a noteworthy accomplishment considering the general configurational lability of 3d-metals compared to its 4d- and 5d-congeners. In this chiral-at-iron catalyst scaffold, iron is coordinated by two chelating *N*-(2-pyridyl)-substituted N-heterocyclic carbenes (PyNHC) in a *C*_2_-symmetrical manner, resulting in a helical topology with an iron center possessing stereogenicity in either the Λ (left-handed helicity) or Δ (right-handed helicity) configuration.^6–8^

The coordination sphere is completed by two acetonitrile molecules, providing an overall octahedral geometry. In this arrangement, the PyNHC ligands are designed to be configurationally stable to preserve the stereochemical information, while the acetonitrile ligands are intended to be labile to facilitate catalysis. Configurational robustness was further improved by replacing the imidazol-2-ylidene carbene moieties with slightly more π-accepting benzimidazol-2-ylidenes.^5*c*^ The obtained benzimidazol-2-ylidene chiral-at-iron complex was demonstrated to be an excellent catalyst for an asymmetric hetero-Diels–Alder reaction under open flask conditions. However, using this class of chiral-at-iron catalysts, thus far we only accomplished chiral Lewis acid catalysis.

To alter the catalytic activity, we envisioned to replace the normal NHC (nNHC) of our bidentate PyNHC ligands with a stronger σ-donating abnormal NHC (aNHC). For that reason, 1,2,3-triazolin-5-ylidene was chosen to substitute the imidazoline-2-ylidene moiety, as the triazole scaffold can be easily synthesized and modified (Fig. 1b).^9^ 1,2,3-Triazolin-5-ylidenes belong to a subclass of aNHCs, which are known as mesoionic carbenes (MICs), due to the zwitterionic character of all its sensible mesomeric structures. Since the first report by Albrecht *et al.* in 2008,^10^ the triazole based aNHCs have been widely accepted as an addition to the toolbox of organometallic chemistry.^9,11^ Recently, several iron triazolin-5-ylidene complexes emerged in the literature, serving as catalyst for the intramolecular C–H amination of organic azides,^12^ or as photosensitizer with long excited-state lifetime.^13^ The simplicity of the triazole core synthesis *via* click chemistry has even been acknowledged in 2022 with a Nobel Prize in chemistry.

Herein, we demonstrate the influence of the strong σ-donating properties of the MIC ligands on the reactivity of the corresponding iron complexes in nitrene transfer catalysis and the modulation in the configurational stability of the corresponding iron complexes. While replacing the imidazol-2-ylidene carbene moieties with 1,2,3-triazolin-5-ylidenes significantly enhances the reactivity of the iron complexes towards a nitrene mediated intramolecular C(sp^3^)–H amidation reaction, the configurational stability is severely impacted. However, a combination of the electron rich 1,2,3-triazolin-5-ylidene MICs with a chiral pinene-modified pyridyl ligand gives rise to a new class of stereogenic-at-iron catalysts (Fig. 1c), which assemble from the ligands in a highly diastereoselective fashion. This new non-racemic stereogenic-at-iron catalyst catalyzes an asymmetric nitrene-mediated intramolecular C(sp^3^)–H amidation to afford chiral 2-imidazolidinones with high yield and satisfactory enantioselectivity.

## Results and discussion

### Design and synthesis of racemic FeMIC complexes

**Scheme 1:**
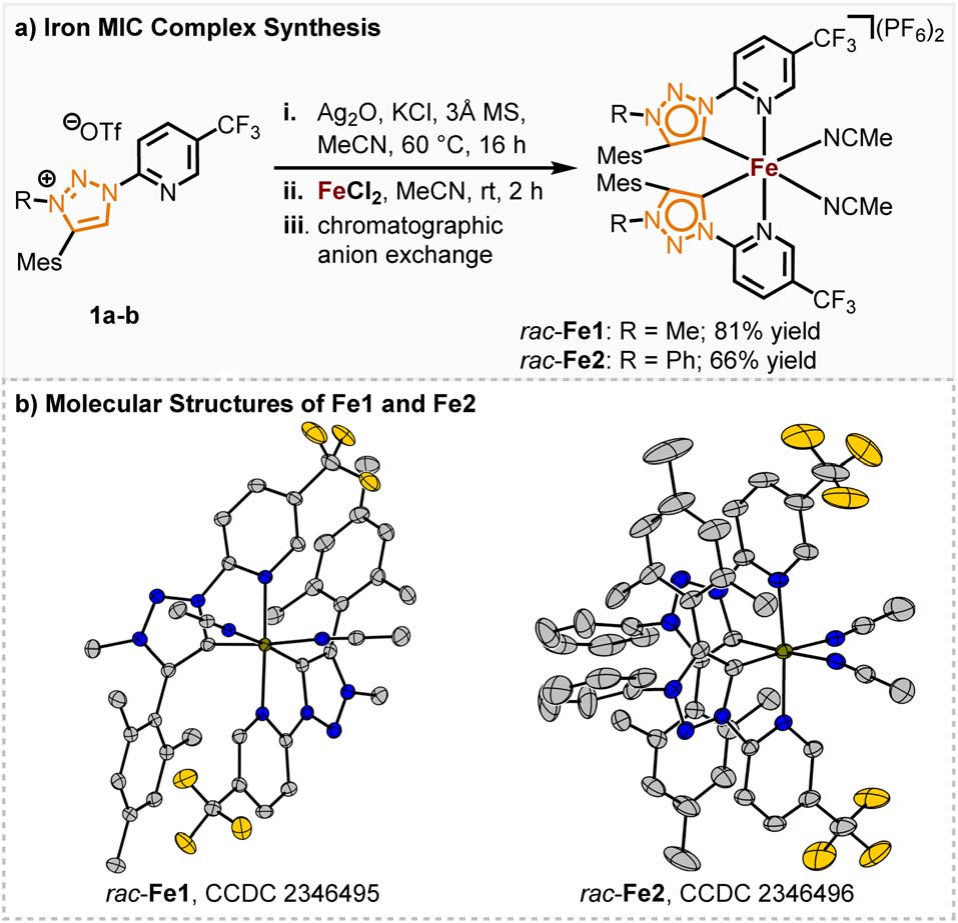
Synthesis and structure of the racemic iron MIC complexes *rac*-Fe1 and *rac*-Fe2.

We started our investigation with the pyridyl triazole ligands 1a, b (Scheme 1). Among others, the research groups of Sarkar, Košmrlj and Albrecht developed multiple synthetic pathways to pyridyl triazoles,^14^ assessing their electronic properties^15^ and implementing them as ligands.^16^ MICs generally exhibit stronger σ-donating properties than traditional nNHCs, leading to a more electron-rich iron center and thereby potentially unlocking novel reactivities. Possessing nearly identical ligand geometries to their nNHC counterparts, altered catalytic properties of the corresponding iron complexes should therefore be traced back to the altered electronic properties of the ligands.

Previously, we accomplished the synthesis of chiral-at-iron complexes bearing achiral nNHC ligands which could be synthesized through electrochemical or standard base induced methods.^5^ While the synthesis was simplified and even the configurational stability significantly enhanced through a fine-tuning of the electronic properties of the 2-pyridyl-NHC ligand, the catalytic reactivity of these complexes towards non-Lewis acid mediated reactions is thus far limited. In this work we investigated the influence of stronger σ-donating, but also weaker π-accepting MIC ligands on the catalytic performance of the corresponding chiral-at-iron complexes. The synthesis of the ligands 1a, b was performed following a slightly modified literature procedure of Košmrlj and co-workers.^14^ Both methylated and arylated triazoline ligands were synthesized for comparison (see ESI† for details of the ligand synthesis). The synthesis of the racemic iron(ii) complexes was achieved *via* a silver carbene route and subsequent *in situ* complex formation after addition of FeCl_2_. After chromatographic anion exchange, the complexes *rac*-Fe1 and *rac*-Fe2 were obtained in 81% and 66% yields, respectively (Scheme 1a). Single crystal X-ray diffraction confirmed the *C*_2_-symmetry of these iron(ii) complexes (Scheme 1b). Both racemic complexes exhibit robustness towards air and water and could be stored under ambient temperature over months and at 4 °C for over 2 years without any sign of decomposition.

We subsequently discovered that the more electron rich iron(ii) complexes *rac*-Fe1, 2 displayed significant catalytic activity in the nitrene-mediated ring-closing C(sp^3^)–H-amidation reaction of *N*-benzoyloxyurea 2 to 2-imidazolidinone 3 (Table 1).^17^ For example, at room temperature after 24 h, *rac*-Fe1 and *rac*-Fe2 at catalyst loadings of 5 mol% provided conversions of over 80% with product NMR yields of 40% and 55%, respectively (entries 1 and 2), while for the previous nNHC congeners *rac*-Fe3 and *rac*-Fe4 the conversions under identical reaction conditions were just 44% and 12% with NMR yields of 18% and 9%, respectively (entries 3 and 4).

**Table 1:** Nitrene-mediated C(sp^3^)–H amidation with iron MIC and NHC complexes^a^

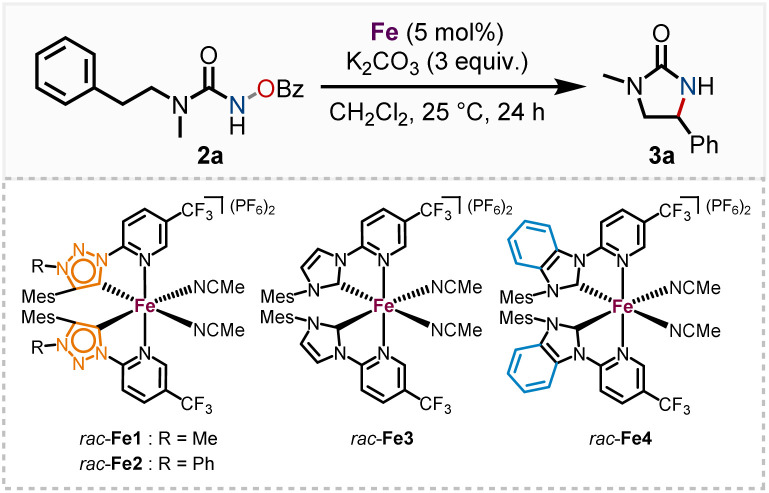			
#	Catalyst	Conversion^b^ (%)	Yield^b^ (%)
1	*rac*-Fe1	85	40
2	*rac*-Fe2	86	55
3	*rac*-Fe3	44	18
4	*rac*-Fe4	12	9

a Reaction conditions: *rac*-Fe1–4 (5 mol%), urea 2 (0.05 mmol) and K_2_CO_3_ (0.15 mmol) were dissolved in distilled CH_2_Cl_2_ (1.00 mL) and stirred under indicated conditions for 24 h.

b Yields and conversion were determined *via*^1^H NMR analysis with 1,1,2,2-tetrachloroethane as standard.

Apparently, the more electron-rich MIC ligands are beneficial for such nitrene-mediated C–H amidation, presumably by increasing the rate of formation of the iron–nitrene intermediate. This aligns with findings of Maldivi and Latour, who asserted that nitrene formation is favored by electron-rich catalysts, while nitrene transfer requires a more electrophilic species.^18^ Investigation of the MIC ligands 1a, b*via* Ganter's ^77^Se NMR method^19^ confirmed the expected decrease in π-accepting properties for the stronger σ-donating MIC ligands. The increase in σ-donation is further supported by the Szostak parameter^20^ (see ESI† for details).

### Non-racemic iron MIC complexes

**Scheme 2:**
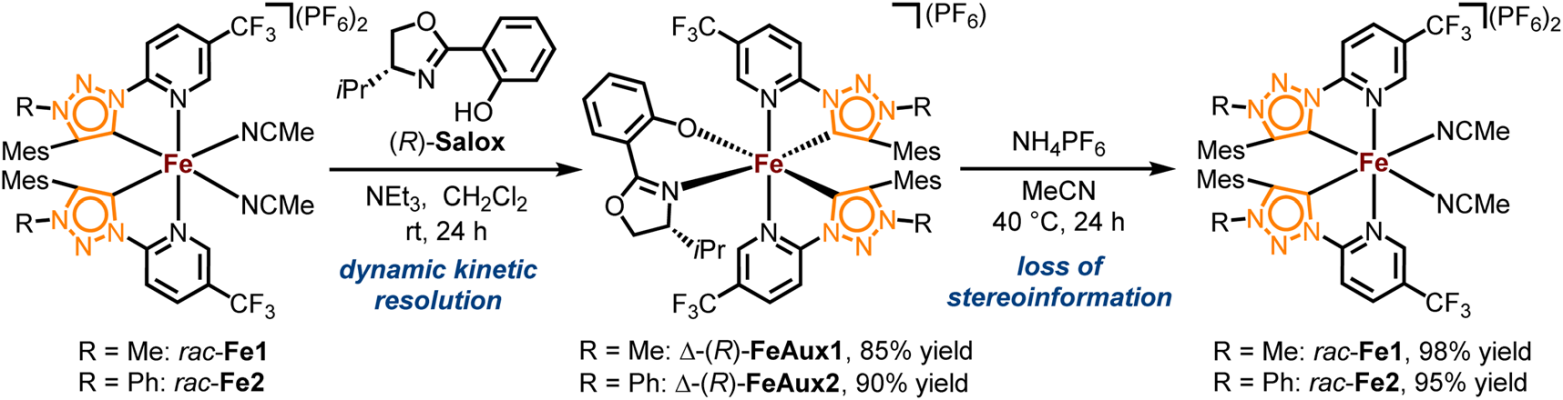
Attempted auxiliary-mediated chiral resolution of iron MIC complexes *rac*-Fe1 and *rac*-Fe2.

The complexes *rac*-Fe1, 2 contain only achiral ligands but are chiral due to the stereogenic iron center. With the aim to next investigate the enantioselective version of the nitrene-mediated ring-closing C(sp^3^)–H amidation, we were seeking to obtain non-racemic iron(ii) triazolin-5-ylidene complexes. Unfortunately, a chiral resolution of *rac*-Fe1, 2 using our previously established auxiliary-mediated route^5^ failed due to an increased configurational lability of these iron MIC complexes compared to the related iron nNHC congeners. Accordingly, using the chiral auxiliary ligand (*R*)-Salox, Δ-(*R*)-FeAux1 and Δ-(*R*)-FeAux2 were obtained with yields of 85% and 90% respectively (Scheme 2), suggesting a dynamic kinetic resolution and hinting at a limited configurational stability of the iron center. It was therefore not surprising that after cleavage of the chiral auxiliary, the iron complexes were obtained as racemic mixtures. Also, exchanging the *N*-5-methyl with a phenyl, which is known to result in a more stable carbene,^21^ had no influence on the configurational stability of the corresponding iron complex. This configurational lability of the MIC complexes can be attributed to the imbalance in the electronic properties of the weaker π-accepting and stronger σ-donating MIC ligands. This is consistent with our previous finding, in which a slight decrease in σ-donating combined with an increase in π-accepting properties afforded a configurational more robust chiral-at-iron complex.^5*c*^ Therefore, we next pursued a strategy employing chiral ligands to control the iron-centered configuration.^22^

**Scheme 3:**
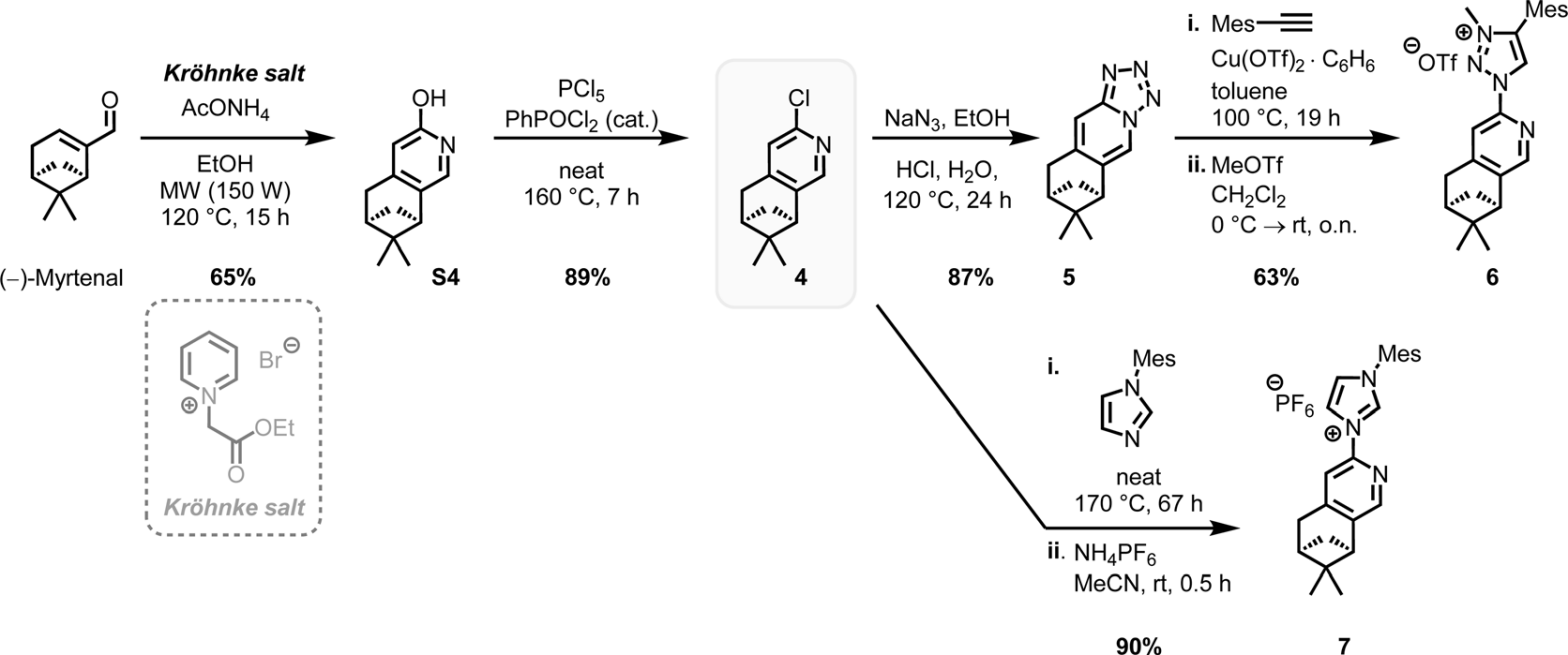
Synthesis of pinene-based ligands 6 and 7 from (−)-myrtenal.

We designed the chiral bidentate MIC ligand 6 as illustrated in Scheme 3 which is based on pinene-modified chiral pyridine ligands previously developed by von Zelewsky.^23^ Considering the inter-ligand stacking between mesityl groups and coordinated pyridines seen in Fe1, 2 and similar complexes involving mesityl groups coordinating pyridines, we predicted that this pinene MIC ligand 6 would exhibit a strong preference for one diastereomer, consequently resulting in a specific metal-centered configuration (see ESI† for more details). For comparison, we also synthesized the corresponding pinene nNHC ligand 7. Both ligands were synthesized from the literature known chloro pyridyl precursor 4 which can be obtained in 2-steps from (−)-myrtenal, a readily available and cheap reagent from the “chiral pool”.^23,24^ We significantly optimized the reaction conditions for the Kröhnke annulation of (−)-myrtenal with the Kröhnke salt using a microwave-assisted setup. This enhancement increased the yield for S4 from 27%^24*b*^ to 65% yield while also reducing the reaction time from 7 d to 15 h. The subsequent deoxochlorination with PCl_5_ and catalytic PhPOCl_2_ to obtain the chloro pyridyl 4 not only significantly improved the yield from 73%^24*a*^ to 89% by utilizing the method from Zhu *et al.*,^25^ but was also now reliably reproducible.

**Scheme 4:**
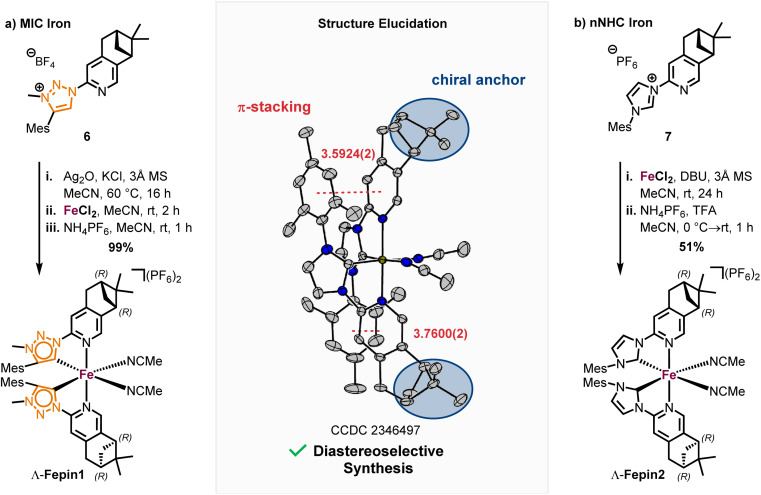
Synthesis of pinene-based stereogenic-at-iron complexes Λ-Fepin1, 2.

With the precursor 4 in hand, the MIC ligand 6 was synthesized *via* a tetrazole formation followed by a CuAAC and subsequent methylation to yield the desired ligand in 55% yield over 3 steps. Following a procedure form Chattopadhyay and co-workers,^26^ tetrazole 5 was formed by an S_N_Ar with NaN_3_ under acidic conditions in 87% yield. The CuAAc was performed in toluene, a solvent with low dielectric constant, and high temperatures of 100 °C to shift the equilibrium of the tetrazole 5 to the open azide form, which then can take part in the CuAAc with mesitylene alkyne.^14*b*^ As the optimal copper catalyst, Cu(OTf)_2_·C_6_H_6_ was chosen, as CuBr(PPh_3_)_3_ showed lower reactivity and higher rate of the undesired Glaser coupling product. In the final step, the triazole was methylated using MeOTf to obtain the desired triazolium ligand 6 with 63% yield over two steps. The corresponding nNHC ligand 7 was synthesized from the precursor 4*via* our previously established S_N_Ar method^5^ with mesityl imidazole under neat conditions with an excellent yield of 90%. The chiral pinene-based MIC ligand 6 was converted to the complex Λ-Fepin1 following the silver carbene route in analogy to the racemic synthesis of *rac*-Fe1, 2 (Scheme 4). It turned out to be beneficial starting from the ligand 6 as its BF_4_ salt, as this ensured a clean conversion to the desired complex and easier purification on the silica column. The counterion of the complex was afterwards readily exchanged with PF_6_ by just stirring the complex with an excess of NH_4_PF_6_ in MeCN. With this, the chiral MIC iron complex Λ-Fepin1 was obtained in quantitative yield (Scheme 4a). ^1^H and ^13^C NMR spectroscopy are consistent with the formation of a single stereoisomer. Furthermore, CD spectroscopy revealed the optical activity of this complex. The corresponding nNHC complex Λ-Fepin2 was synthesized utilizing our previously developed organic base mediated method (Scheme 4b).^5*c*^ However, due to the less acidic character of the NHC, a stronger base had to be used, in this case DBU.

Since we observed DBU coordinating to the newly formed complex, TFA was added afterwards under presence of additional NH_4_PF_6_ to ensure a complete dissociation of DBU and exchange with acetonitrile. After recrystallization from i-PrOH the complex was obtained in a yield of 51%. Also here, NMR spectroscopy revealed the formation of a single diastereomer. A structural confirmation *via* single crystal XRD showed, that only one enantiomer and diastereomer of the complex is present, due to the chiral anchor favoring the inter-ligand π-stacking from one site which leads to the formation of the Λ-configuration at the iron center (Scheme 4, see also ESI† for more details). With this, an enantiomerically and diastereomerically pure complex was formed without any chiral resolution necessary. For comparison, for previously reported rhodium based chiral-at-metal complexes bearing chiral pinene-based ligands, always both Λ- and Δ-diastereomers were obtained.^27^ Furthermore, the complexes showed no sign of configurational lability after 1 week in acetonitrile under air. However, in the non-coordinating solvent dichloromethane and in the absence of any potential exogeneous ligand, slow decomposition was observed, which we attribute to a dissociation of at least one MeCN ligand to provide a coordinatively unsaturated, less stable iron complex.^28^ The steric maps of the iron MIC/NHC complexes bearing achiral or chiral ligands, as well the formation of both possible configurational isomers of the chiral iron complexes are further discussed in the ESI.†

A highly diastereoselective formation of octahedral iron complexes from bidentate ligands is rare. For example, Scott reported the diastereoselective formation of *fac*-tris(iminopyridine) complexes,^29^ while Yamamoto used a binaphthyl-modified 2,9-phenanthroline for diastereoselective iron-coordination.^30^

### Enantioselective catalysis

With the non-racemic stereogenic-at-iron complexes Λ-Fepin1, 2 in hand, we next investigated the asymmetric version of the nitrene-mediated ring-closing C(sp^3^)–H amidation to 2-imidazolidinones (Table 2). First, Λ-Fepin1 and Λ-Fepin2 were tested with standard substrate 2a showing a conversion of 97% and 75% NMR yield with an enantiomeric ratio of 82 : 18 for the MIC complex Λ-Fepin1 (entry 1), while for the nNHC complex Λ-Fepin2 a conversion of only 61% and NMR yield of just 36% was obtained (entry 2). As expected, the more electron-rich MIC complex displayed superior catalytic activity compared to the nNHC complex and provided an encouraging enantioselectivity. Furthermore, the higher catalytic activity of the MIC complex Λ-Fepin1 (Table 2, entry 1) compared to the MIC complex *rac*-Fe1 (Table 1, entry 1) can be rationalized with a higher electron density at the iron in Λ-Fepin1 due to a more electron rich pyridyl ligand (CF_3_ replaced with pinene moiety). Next, in order to optimize this conversion, the role of the benzoate leaving group was investigated (entries 3–6). As a result, the more electron rich and bulky leaving groups 4-*t*bu-Bz 2b and 3,5-Me_2_-Bz 2c showed both a diminished conversion of 42% and 48%, respectively, with an er of 82 : 18 for both leaving groups (entries 3 and 4). The electron withdrawing CF_3_-group 3,5-(CF_3_)_2_-Bz 2d showed almost full conversion of 97% with an NMR yield of 54% but a reduced er of 80 : 20 for Λ-Fepin1 (entry 5). Also, Λ-Fepin2 was tested with 3,5-(CF_3_)_2_-Bz 2d as the leaving group, revealing an increased conversion of 87% compared to the Bz leaving group. The NMR yield remained low with 41% but displaying an enantioselectivity of 83 : 17 er comparable with Λ-Fepin1 (entry 6). The leaving group screen revealed, that altering the electronic properties of the leaving group only influenced the conversion and yield but had no effect on the enantioselectivity of the C–H amidation product, suggesting that the leaving group does not remain coordinated to the iron center after N–O bond cleavage. This is in contrast to previous work from our group, where utilizing an Fe–N_4_ catalyst showed that coordination of the carboxylate leaving group to the iron center greatly influences the enantioselectivity in the ring closing C–H amidation.^17*c*^ Next, the influence of increased sterics on the methylated nitrogen was investigated by replacing the methyl moiety with either an ethyl (2e, entry 7) or *n*-butyl (2f, entry 8). Both reactions provided almost full conversion (97% for 2e and 95% 2f) with 75% and 67% NMR yield and 78 : 22 and 76 : 24 er, respectively. This revealed that an increase in steric bulk on the nitrogen leads diminished enantioselectivity, while reactivity remained almost unchanged. To our surprise, changing the counterion of the complex from PF_6_ to BF_4_ resulted in diminished product formation of 26% with almost full conversion, thus demonstrating a counterion effect for this ring-closing C–H amidation (entry 9). Lastly, we tested the influence of temperature on the reaction (entries 10 and 11). At 4 °C, full conversion was achieved with an increased NMR yield of 90% and slightly higher er of 84 : 16 (entry 10). At −10 °C, full conversion was still maintained with a similar NMR yield of 89% and an er of 86 : 14 (entry 11). By lowering the temperature, we could observe an increased stability of the catalyst in solution, marked by a colored solution, enabling an efficient participation of the iron catalyst in the reaction pathway, which leads to an increased yield. As expected, even the enantiomeric ratio could be slightly increased from 82 : 18 at 25 °C to 86 : 14 at −10 °C. Further decreasing the temperature was not feasible, as the reaction rate strongly decreased. In entries 12–14 steric effects were investigated by substituting a hydrogen in *para*-, *meta*-, or *ortho*-position of the phenyl ring with a methyl moiety, leading to er values of 86 : 14 (2g), 84 : 16 (2h) and 91 : 9 (2i), respectively, with yields ranging from 80–90% at 4 °C. To our surprise, a steric increase in the *ortho*-position positively effected the enantiomeric ratio in contrary to our previous published results.^17*c*^ The er could even be further increased to 92 : 8 at −10 °C (entry 15).

**Table 2:** Enantioselective nitrene-mediated C(sp^3^)–H amidation with iron MIC and NHC complexes^a^

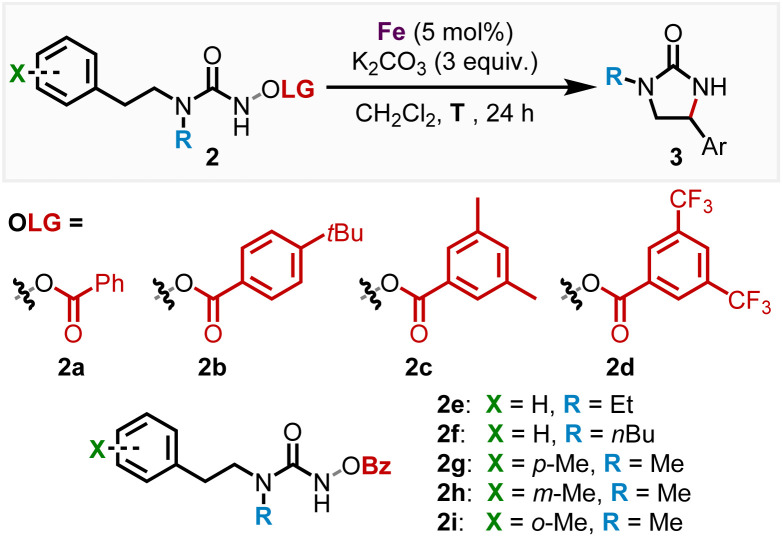						
#	Catalyst	*T* (°C)	Conversion^b^ (%)	Substr.	Yield^b^ (%)	er^c^
1	Λ-Fepin1	25	97	2a	75	82 : 18
2	Λ-Fepin2	25	61	2a	36	nd
3	Λ-Fepin1	25	42	2b	nd	82 : 18
4	Λ-Fepin1	25	48	2c	nd	82 : 18
5	Λ-Fepin1	25	97	2d	54	80 : 20
6	Λ-Fepin2	25	87	2d	41	83 : 17
7	Λ-Fepin1	25	97	2e	75	78 : 22
8	Λ-Fepin1	25	95	2f	67	76 : 24
9	Λ-Fepin1BF_4_	25	97	2a	26	nd
10	Λ-Fepin1	4	100	2a	90	84 : 16
11	Λ-Fepin1	−10	100	2a	89	86 : 14
12	Λ-Fepin1	4	100	2g	90	86 : 14
13	Λ-Fepin1	4	100	2h	80	84 : 16
14	Λ-Fepin1	4	100	2i	80	91 : 9
15	Λ-Fepin1	−10	100	2i	81	92 : 8

a Reaction conditions: Λ-Fepin1, 2 (5 mol%), urea 2a–i (0.05 mmol) and K_2_CO_3_ (0.15 mmol) were dissolved in distilled CH_2_Cl_2_ (1.00 mL) and stirred under indicated conditions for 24 h.

b Yields and conversion were determined *via*^1^H NMR analysis with 1,1,2,2-tetrachloroethane as standard.

c er were determined by HPLC analysis on a chiral stationary phase; nd = not determined.

## Conclusions

In conclusion, this work investigated the influence of strongly σ-donating 1,2,3-triazolin-5-ylidene mesoionic carbene ligands (MICs) on the configurational stability and reactivity of stereogenic-at-iron complexes. *C*_2_-symmetric iron complexes with two coordinated pyridyl triazolin-5-ylidene ligands display an increased catalytic activity compared to their imidazol-2-ylidene congeners regarding a nitrene-mediated ring-closing C(sp^3^)–H amidation. However, the MIC ligands render the stereogenic iron center configurationally labile. To nevertheless achieve the generation of non-racemic complexes, the pyridyl moiety was functionalized with a chiral pinene moiety which resulted in the formation of a single diastereomer in the course of ligand coordination. We subsequently demonstrated that a non-racemic pinene-modified iron MIC complex can catalyze the ring-closing C–H amidation of *N*-benzoyloxyurea to 2-imidazolidinones with an enantiomeric ratio of up to 92 : 8. Future work will investigate other applications for this new class of chiral iron MIC catalysts.

## Author contributions

N. D. and E. M. conceived and designed the project. N. D. performed the majority of the synthetic experiments and analyzed the data. M. D. and G. N. contributed with synthetic experiments. S. I. I. performed the X-ray crystallography. N. D. and E. M. wrote the manuscript.

## Conflicts of interest

The authors declare no conflict of interest.

## Supplementary Material

SC-015-D4SC03504F-s001

SC-015-D4SC03504F-s002

## Data Availability

The ESI† contains detailed synthetic procedures and complete characterization data for all new compounds, CD spectra of all non-racemic iron complexes, as well UV/vis absorbance spectra of the here discussed iron complexes. Investigation of the electronic properties of the MIC ligands and detailed steric maps of the iron complexes are further discussed in the ESI.†
