# Supplementary material for: Stereogenic-at-iron mesoionic carbene complex for enantioselective C–H amidation
Source: Chem Sci. 2024 Sep 10;15(38):15625–31. doi: 10.1039/d4sc03504f (PMC11385695; doi:10.1039/d4sc03504f)
Supplement: SC-015-D4SC03504F-s001 [file SC-015-D4SC03504F-s001.pdf]

Supplementary Information (SI) for Chemical Science.
This journal is © The Royal Society of Chemistry 2024

Supplementary Information

Stereogenic-at-Iron Mesoionic Carbene Complex for Enantioselective C-H Amidation

Nemrud Demirel, Mahiob Dawor, Greta Nadler, Sergei I. Ivlev, and Eric Meggers*

Fachbereich Chemie, Philipps-Universität Marburg, Hans-Meerwein-Strasse 4, 35043 Marburg, Germany

*Corresponding author: meggers@chemie.uni-marburg.de

Table of Contents

1. **General Information** S1
2. **Ligand Synthesis** S2
3. **Iron Complex Synthesis** S13
4. **Auxiliary Complex Synthesis** S26
5. **Auxiliary Cleavage** S30
6. **Investigation of the Electronic Properties of MIC Ligands** S31
7. **Steric Maps of Iron MIC Complexes** S38
8. **Iron-Catalyzed Ring-Closing C(sp^3)-H Amidation** S40
9. **Stability and Reactivity of Fepin Complexes** S42
10. **NMR Spectra** S46
11. **Chiral HPLC Traces** S72
12. **CD-Spectra** S75
13. **UV-Visible Spectra** S77
14. **Single Crystal X-Ray Diffraction** S78
15. **References** S86

1. General Information

All reactions were carried out under a nitrogen atmosphere in oven-dried glassware unless noted otherwise. Solvents were distilled under nitrogen from sodium/benzophenone (THF, Et_2O) or calcium hydride (MeCN, CH_2Cl_2, $CHCl_3$, toluene) prior to use. Reagents that were purchased from commercial suppliers were used without further purification. Flash column chromatography was performed with silica gel 60 M from Macherey-Nagel (irregular shaped, 230−400 mesh, pH 6.8, pore volume: 0.81 mL g^{-1}, mean pore size: 66 Å, specific surface: 492 m^2 g^{-1}, particle size distribution: 0.5% < 25 µm and 1.7% > 71 µm, water content: 1.6%). ^{1}H NMR, ^{13}C {^{1}H} NMR, ^{19}F{^{1}H} NMR and ^{77}Se{^{1}H} NMR spectra were recorded on a Bruker AV NEO 300 MHz, AV II 300 MHz, AV III 500 MHz, or AV III HD 500 MHz spectrometer at ambient temperature. Chemical shift values δ are reported in ppm with the solvent resonance as internal standard. ^{19}F{^{1}H} NMR spectra were calibrated to trichlorofluoromethane ($CFCl_3$, δ = 0 ppm) as external standard. ^{77}Se{^{1}H} NMR spectra were calibrated to dimethyl selenide ($SeMe_2$, δ = 0 ppm) as external standard. IR spectra were recorded on a Bruker Alpha FT−IR spectrometer. Chiral HPLC was performed on an Agilent 1200 or 1260. CD spectra were acquired with a JASCO J-810 CD spectropolarimeter (parameters: 600−200 nm, 1 nm band-width, 50 nm min^{-1} scanning speed, accumulation of 3 scans). UV/Vis absorbance spectra were recorded on a JASCO V-650 in a 10.0 mm quartz cuvette (parameters: 900-230 nm, 2.0 nm bandwidth, 400 nm/min scanning speed, 0.5 nm data interval). Melting points (MPs) were determined on a Mettler Toledo MP70 using one end closed capillary tubes. High-resolution mass spectrometry was performed on a Finnigan LTQ-FT Ultra mass spectrometer (Thermo Fischer Scientific) using ESI or APCI as ionization source. Optical rotations were measured with a Krüss P8000-T polarimeter with $[\alpha]_D^{22}$ values listed in degrees with concentrations reported in g/100 mL. Compounds **S1**[1] and **S2**[2] were synthesized after a literature procedure.

2. Ligand Synthesis

Procedure for the Racemic Ligands 1a-b

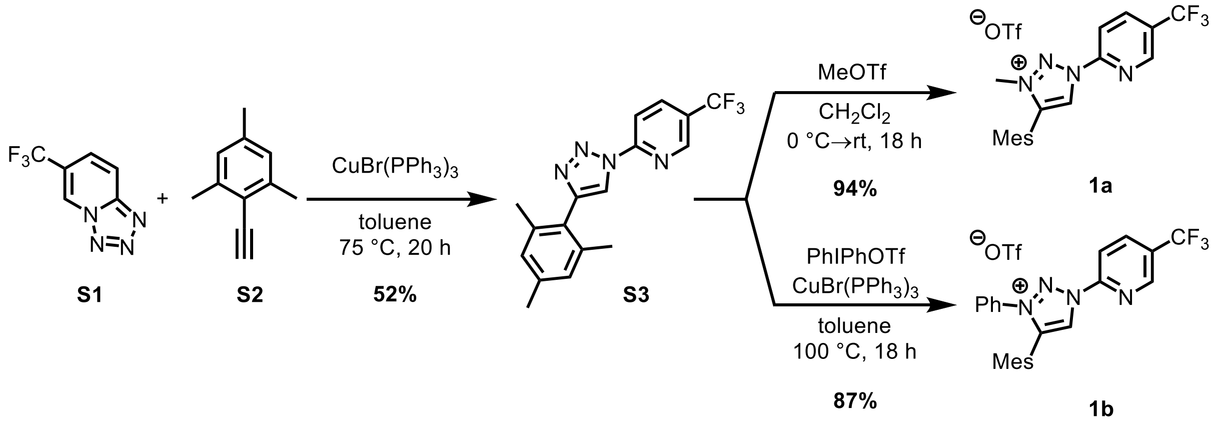

2-(4-mesityl-1H-1,2,3-triazol-1-yl)-5-(trifluoromethyl)pyridine (S3)

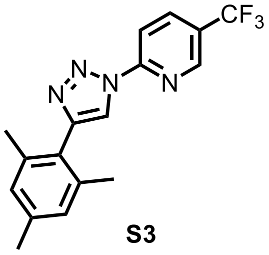

S3

Following a modified procedure of Košmrlj *et al.*[3] Tetrazole **S1** (1.00 g, 5.32 mmol, 1.00 eq) and $CuBr(PPh_3)_3$ (247 mg, 267 μmol, 0.05 eq) were dissolved in toluene (70.0 mL) under nitrogen atmosphere. Subsequently, alkyne **S2** (0.92 g, 6.38 mmol, 1.20 eq) was added and the reaction mixture was put in a pre-heated heating block and stirred at 75 °C for20 h. After full conversion was confirmed via TLC, the solvent was removed under reduced pressure and the crude product was purified by column chromatography (silica, *n*-Pentane/EtOAc 100:0 → 20:1 → 15:1 → 10:1) to obtain Triazole **S3** (0.92 g, 2.79 mmol, 52%) as a colorless solid.

TLC (*n*-Pentane/EtOAc 10:1): R_f=0.47. **MP** 120–121 °C. **^{1}H NMR** (300 MHz, $CDCl_3$): δ = 8.80 (s, 1H), 8.52 (s, 1H), 8.43 (d, J = 8.6 Hz, 1H), 8.19 (dd, J = 8.6, 2.4 Hz, 1H), 6.99 (s, 2H), 2.35 (s, 3H), 2.18 (s, 6H) ppm. **^{13}C NMR** (75 MHz, $CDCl_3$): δ = 151.60, 146.63, 146.17 ($J_{C,F}$ = 4.4 Hz), 138.66, 137.86, 136.70 ($J_{C,F}$ = 3.8 Hz), 128.63, 126.42, 126.35 ($J_{C,F}$ = 33.1 Hz), 123.22 ($J_{C,F}$ = 272.3 Hz), 120.33, 113.66, 21.18, 20.18 (2C) ppm. **^{19}F NMR** (282 MHz, $CDCl_3$): δ = –62.19 (s, CF_3) ppm. **IR** (neat): $\tilde{\nu}$ = 3192 (w), 3113 (w), 3083 (w), 2972 (w), 2922 (w), 2864 (w), 1728 (w), 1607 (m), 1495 (w), 1477 (w), 1446 (m), 1378 (w), 1325 (m), 1265 (w), 1222 (m), 1193 (w), 1166 (m),

1121 (s), 1087 (w), 1072 (w), 1031 (m), 996 (w), 941 (w), 860 (m), 849 (w), 814 (m), 795 (w), 761 (w), 743 (w), 715 (w), 655 (w), 637 (w), 610 (w), 572 (w), 516 (w), 475 (w), 442 (w), 415 (w) cm^{-1}. **HRMS** ESI; *m/z* calcd. for $C_{17}H_{15}F_3N_4H_1$ $[M]^+$: 333.1322, found: 333.1312.

4-Mesityl-3-methyl-1-(5-(trifluoromethyl)pyridin-2-yl)-1*H*-1,2,3-triazol-3-ium OTf (1a)

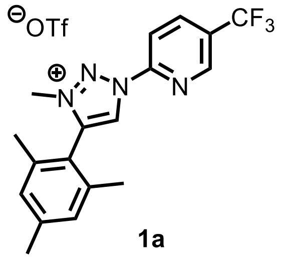

1a

Following a modified procedure of Košmrlj *et al.*[4] Triazole **S3** (0.69 g, 2.08 mmol, 1.00 eq) was dissolved in CH_2Cl_2 (10.0 mL) under nitrogen atmosphere. The reaction mixture was cooled to 0 °C and MeOTf (219 µL, 2.49 mmol, 1.20 eq) was added and the reaction mixture was allowed to warm up to room temperature and stirred overnight for18 h. After full conversion was confirmed via TLC, the reaction mixture was filtered over a silica pad and rinsed with CH_2Cl_2 to wash out remaining starting material and impurities. The product was then eluted from the silica pad with a mixture of CH_2Cl_2/MeOH 10:1. After removal of the solvents under reduced pressure Triazolium **1a** (0.97 g, 1.95 mmol, 94%) was obtained as an off-white solid.

TLC (CH_2Cl_2/MeOH 10:1): R_f=0.31. **MP** 66–67 °C. **^{1}H NMR** (300 MHz, $CDCl_3$): δ = 9.02 (s, 1H), 8.86 (dd, J = 1.5, 0.8 Hz, 1H), 8.60 (d, J = 8.6 Hz, 1H), 8.37 (dd, J = 8.7, 2.3 Hz, 1H), 7.07 (s, 2H), 4.23 (s, 3H, N-CH_3), 2.38 (s, 3H), 2.16 (s, 6H) ppm. **^{13}C NMR** (75 MHz, $CDCl_3$): δ = 148.93, 146.38 (q, $^3J_{C,F}$ = 4.1 Hz), 143.38, 143.12, 138.83, 138.35 (q, $^3J_{C,F}$ = 3.5 Hz), 129.97 (q, $^2J_{C,F}$ = 34.2 Hz), 129.63, 125.79, 122.60 (q, $^1J_{C,F}$ = 273.2 Hz), 120.72 (q, $^1J_{C,F}$ = 320.6 Hz, $^-OSO_2CF_3$), 117.15, 116.80, 38.76, 21.43, 20.24. ppm. **^{19}F NMR** (282 MHz, $CDCl_3$): δ = –62.44 (s, CF_3), –78.49 (s, $^-OSO_2CF_3$) ppm. **IR** (neat): $\tilde{\nu}$ = 3081 (w), 2926 (w), 1605 (m), 1483 (w), 1436 (w), 1389 (w), 1327 (s), 1258 (s), 1224 (w), 1134 (s), 1075 (m), 1030 (s), 997 (w), 942 (w), 853 (w), 797 (w), 756 (w), 734 (w), 695 (w), 636 (s), 574 (w), 517 (w), 469 (w), 444 (w), 426 (w) cm^{-1}. **HRMS** ESI; *m/z* calcd. for $C_{18}H_{18}F_3N_4$ $[M – OTf]^+$: 347.1478, found: 347.1465.

4-Mesityl-3-phenyl-1-(5-(trifluoromethyl)pyridin-2-yl)-1*H*-1,2,3-triazol-3-ium OTf (1b)

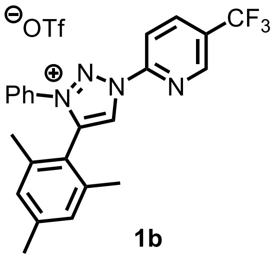

1b

Following a modified procedure of Košmrlj *et al.*[5] Triazole **S3** (0.20 g, 0.60 mmol, 1.00 eq), Diphenyliodonium-trifluormethanesulfonate* (0.31 g, 0.72 mmol, 1.20 eq) and $CuBr(PPh_3)_3$ (28.0 mg, 30.0 µmol, 0.05 eq) were dissolved in toluene (6.0 mL) under nitrogen atmosphere. The reaction mixture was heated to 100 °C and stirred for18 h. Then, the solution was filtered over a pad of Celite and the residue rinsed with toluene. The solvent was removed under reduced pressure and the crude product was purified by column chromatography (silica, CH_2Cl_2/Acetone 20:1 → 10:1 → 5:1) to obtain Triazolium **1b** (293 mg, 0.52 mmol, 87%) as a yellow solid.

* The quality of the Iodonium salt has a big influence on the yield of the reaction, as well the formation of side products.

TLC (CH_2Cl_2/Acetone 5:1): R_f=0.36. **MP** 87–88 °C. **^{1}H NMR** (300 MHz, $CDCl_3$): δ = 9.19 (s, 1H), 8.91 (s, 1H), 8.69 (d, J = 8.6 Hz, 1H), 8.41 (d, J = 9.0 Hz, 1H), 7.66-7.49 (m, 5H), 6.67 (s, 2H), 2.34 (s, 3H), 2.15 (s, 6H) ppm. **^{13}C NMR** (125 MHz, $CDCl_3$): δ = 148.96, 146.43 (q, $^3J_{C,F}$ = 3.8 Hz), 142.82, 142.72, 138.58, 138.24 (q, $^3J_{C,F}$ = 3.8 Hz), 134.22, 132.46, 130.29 (2C), 129.99 (q, $^2J_{C,F}$ = 34.0 Hz), 129.58 (2C), 127.17, 124.41 (2C), 122.59 (q, $^1J_{C,F}$ = 273.0 Hz), 120.71 (q, $^1J_{C,F}$ = 331.2 Hz, $^-OSO_2CF_3$), 117.75, 116.95, 21.41, 20.42 (2C) ppm. **^{19}F NMR** (282 MHz, $CDCl_3$): δ = –62.38 (s, CF_3), –78.46 (s, $^-OSO_2CF_3$) ppm.(at –62.58 ppm is the peak for the minor sideproduct) **IR** (neat): $\tilde{\nu}$ = 3070 (w), 1732 (w), 1603 (m), 1484 (w), 1461 (w), 1434 (w), 1393 (w), 1328 (s), 1261 (s), 1224 (w), 1139 (s), 1076 (m), 1030 (m), 1003 (w), 941 (w), 852 (w), 796 (w), 770 (w), 719 (w), 691 (w), 637 (s), 573 (w), 517 (w), 490 (w), 437 (w). cm^{-1}. **HRMS** ESI; *m/z* calcd. for $C_{23}H_{20}F_3N_4$ [M – OTf]$^+$: 409.1635, found: 409.1625.

Procedure for the Chloro-Precursor 4

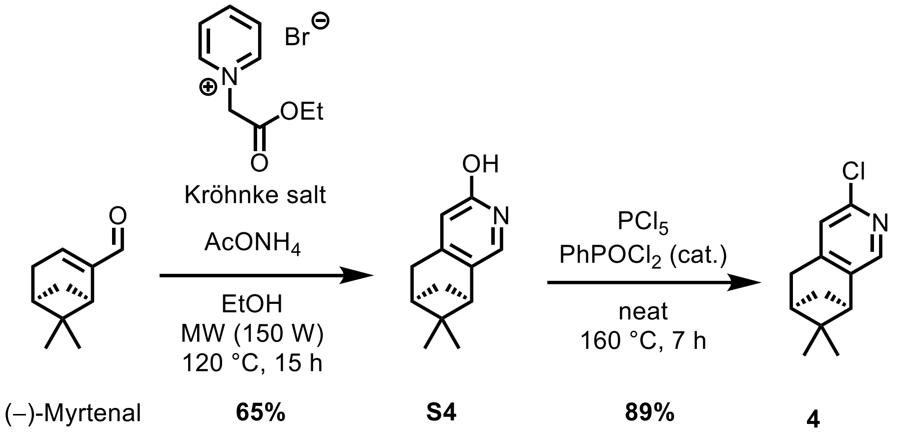

1-(2-ethoxy-2-oxoethyl)pyridin-1-ium bromide (Kröhnke salt)

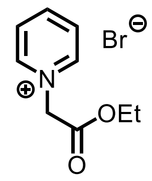

Kröhnke salt

Following a modified literature procedure,[6] ethyl α-bromacetate (5.59 mL, 50.2 mmol, 1.00 eq) was added drop by drop to a solution of pyridine (4.05 mL, 50.2 mmol, 1.00 eq) in Ethanol (10.0 mL) and the reaction mixture was heated at 50 °C for 1.5 h. The solvent was removed under reduced pressure and without further purification the Kröhnke salt was obtained (12.0 g, 48.7 mmol, 97%) as an off-white solid.

MP 138–139 °C. **^{1}H NMR** (300 MHz, $CDCl_3$): δ = 9.50 (d, J = 6.0 Hz, 2H), 8.53 (t, J = 7.8 Hz, 1H), 8.06 (t, J = 6.9 Hz, 2H), 8.38 (s, 2H), 4.28 (q, J = 7.1 Hz, 2H), 1.31 (t, J = 7.1 Hz, 3H) ppm. **^{13}C NMR** (75 MHz, $CDCl_3$): δ = 165.71, 146.59 (2C), 146.40, 127.80 (2C), 63.26, 60.87, 14.04 ppm. **IR** (neat): $\tilde{\nu}$ = 3417 (w), 3135 (w), 3033 (w), 2980 (w), 2202 (w), 1743 (m), 1636 (w), 1581 (w), 1490 (m), 1433 (w), 1396 (w), 1374 (w), 1348 (w), 1300 (w), 1214 (s), 1099 (w), 1019 (m), 979 (w), 917 (m), 881 (w), 827 (w), 779 (w), 722 (s), 674 (w), 642 (w), 572 (w), 458 (w) cm^{-1}. **HRMS** ESI; *m/z* calcd. for $C_9H_{12}N_1O_2$ $[M - Br]^+$: 166.0863, found: 166.0857.

The analytics are in agreement with literature.[6]

(6*R*,8*R*)-7,7-dimethyl-5,6,7,8-tetrahydro-6,8-methanoisoquinolin-3-ol (S4)

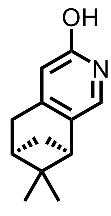

S4

Following a modified procedure from Sala *et al.*,[7] Kröhnke salt (5.04 g, 20.5 mmol, 1.20 eq) and ammonium formiate (6.58 g, 85.4 mmol, 5.00 eq) were dissolved in EtOH (46.0 mL). To the pale-yellow solution (–)-myrtenal (2.59 mL, 17.1 mmol, 1.00 eq) was added. The reaction was conducted in a microwave where the mixture was stirred at 120 °C and 150 W for 15 h. The complete conversion of the starting material was confirmed by TLC. The reaction mixture was transferred with EtOAc (10.0 mL) and the solvents evaporated under reduced pressure (important: thorough removal of EtOH). The solution was washed with H_2O (10.0 mL) and extracted with CH_2Cl_2 (5 x 30.0 mL). The combined organic phases were dried over Na_2SO_4, filtered over Celite and the solvent was removed under reduced pressure and the crude product was purified by column chromatography (silica, CH_2Cl_2/EtOAc 1:1 → EtOAc/MeOH 9:1) to obtain tetrahydroquinolinol **S4** (2.51 g, 13.2 mmol, 65%) as a pale-yellow solid.

TLC (EtOAc/MeOH 9:1): R_f=0.31. **MP** 137–138 °C. **^{1}H NMR** (300 MHz, $CDCl_3$): δ = 12.99 (br s, 1H), 6.89 (s, 1H), 6.38 (s, 1H), 2.90-2.78 (m, 2H), 2.65-2.50 (m, 2H), 2.19-2.09 (m, 1H), 1.31 (s, 3H), 1.13 (d, J = 9.3 Hz, 1H), 0.64 (s, 3H) ppm. **^{13}C NMR** (75 MHz, $CDCl_3$): δ = 165.40, 152.42, 127.79, 126.47, 118.09, 43.88, 40.09, 39.46, 32.88, 32.31, 25.92, 21.53 ppm. **IR** (neat): $\tilde{\nu}$ = 3122 (w), 2923 (w), 2229 (w), 1662 (s), 1623 (m), 1533 (w), 1469 (w), 1435 (w), 1419 (w), 1385 (w), 1370 (w), 1269 (w), 1222 (w), 1186 (w), 1144 (w), 1125 (w), 1071 (w), 905 (s), 857 (w), 761 (w), 724 (s), 644 (w), 609 (w), 570 (w), 512 (w), 483 (m), 431 (w), 413 (w) cm^{-1}. **HRMS** ESI; *m/z* calcd. for $C_{12}H_{15}N_1O_1H_1$ $[M]^+$: 190.1226, found: 190.1222.

$[\alpha]_D^{23}$ = –50.6° (c = 1.0, CH_2Cl_2) {Lit.:[7] **$[\alpha]_D^{25}$** = –50.3°(CH_2Cl_2)}

The analytics are in agreement with literature.[7]

(6*R*,8*R*)-3-chloro-7,7-dimethyl-5,6,7,8-tetrahydro-6,8-methanoisoquinoline (4)

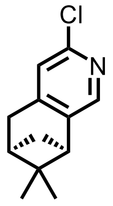

4

Following a procedure from Zhu *et al.*,[8] tetrahydroquinolinol **S4** (0.55 g, 2.90 mmol, 1.00 eq), PCl_5 (0.66 g, 3.19 mmol, 1.10 eq) and phenylphosphonic dichloride (44.0 µL, 0.32 mmol, 0.11 eq) were put in a pressure tube under nitrogen atmosphere. The reaction mixture was put in a heating block and stirred at 160 °C. After 1 h the by-product ($POCl_3$) was removed under vacuum over a cooling trap. The reaction was stirred further for 7 h. After complete conversion of the starting material was confirmed by TLC, the remaining $POCl_3$ was removed under vacuum and the reaction mixture quenched with ice water. The mixture was neutralized with aq. Na_2CO_3-solution and extracted with CH_2Cl_2 (3 x 30.0 mL). The combined organic phases were washed with H_2O (25 mL) and dried over Na_2SO_4, filtered over Celite and the solvent was removed under reduced pressure. The crude product was purified by column chromatography (silica, *n*-Pentane/EtOAc 15:1 to obtain chloro-tetrahydroquinoline **4** (534 mg, 2.57 mmol, 89%) as a pale-yellow solid.

TLC (*n*-Pentane/EtOAc 15:1): R_f=0.77. **MP** 43–44 °C. **^{1}H NMR** (300 MHz, $CDCl_3$): δ = 7.91 (s, 1H), 7.10 (s, 1H), 2.99-2.92 (m, 2H), 2.81 (t, *J* = 5.2 Hz, 1H), 2.69 (dt, *J* = 10.4, 5.2 Hz 1H), 2.32-2.25 (m, 1H), 1.40 (s, 3H), 1.17 (d, *J* = 9.6 Hz, 1H), 0.62 (s, 3H) ppm. **^{13}C NMR** (75 MHz, $CDCl_3$): δ = 148.89, 148.04, 145.44, 141.49, 123.35, 44.02, 39.71, 39.13, 32.65, 31.67, 25.88, 21.31 ppm. **IR** (neat): $\tilde{\nu}$ = 2973 (w), 2932 (m), 2830 (w), 2234 (w), 1592 (m), 1554 (m), 1521 (w), 1467 (s), 1424 (w), 1385 (w), 1366 (s), 1287 (w), 1264 (w), 1237 (w), 1221 (w), 1204 (w), 1173 (m), 1100 (w), 1074 (s), 1027 (w), 947 (w), 922 (w), 901 (s), 863 (m), 836 (w), 762 (w), 730 (m), 691 (m), 642 (w), 606 (w), 486 (w), 465 (w), 435 (m) cm^{-1}. **HRMS** ESI; *m/z* calcd. for $C_{12}H_{14}Cl_1N_1H_1$ $[M]^+$: 208.0899, found: 208.0882.

$[\alpha]_D^{23}$ = –53.5° (*c* = 1.0, $CHCl_3$) {Lit.:[9] **$[\alpha]_D^{25}$** = –54.5°(c = 3.19, $CHCl_3$)}

The analytics are in agreement with literature.[9]

Procedure for the Chiral Pinene-based MIC-Ligand 6

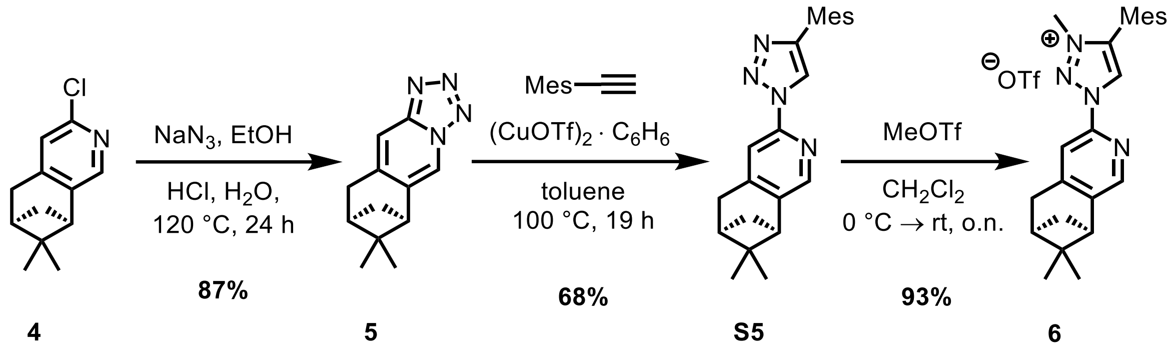

(6*R*,8*R*)-7,7-dimethyl-6,7,8,9-tetrahydro-6,8-methanotetrazolo[1,5-*b*]isoquinoline (5)

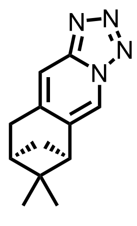

5

Following a modified procedure from Chattopadhyay *et al.*,[10] a pressure tube was charged with chloro-tetrahydroquinoline **4** (520 mg, 2.50 mmol, 1.00 eq) and NaN_3 (325 mg, 5.00 mmol, 2.00 eq) and put under nitrogen atmosphere. Subsequently, EtOH (1.02 mL), H_2O (5.02 mL) and conc. HCl (37%, 0.54 mL) were added to the reaction mixture and the pressure tube was sealed tightly and heated at 120 °C for 24 h (**Caution: The preparation was done very carefully and an explosive shield was used**). After the reaction mixture was cooled down to room temperature, the complete conversion of the starting material was confirmed by TLC. The reaction mixture was diluted with H_2O (50 mL) and neutralized with aq. $NaHCO_3$ solution and extracted with EtOAc (3 x 50.0 mL). The combined organic phases were washed with brine, dried over Na_2SO_4, filtered over Celite and the solvent was removed under reduced pressure. The crude product was purified by column chromatography (silica, *n*-Pentane/EtOAc 5:1 → 2:1) to obtain tetrazole **5** (468 mg, 2.18 mmol, 87%) as a colorless solid.

TLC (*n*-Pentane/EtOAc 2.5:1): R_f=0.21. **MP** 100–101 °C. **^{1}H NMR** (300 MHz, $CDCl_3$): δ = 8.32 (s, 1H), 7.69 (s, 1H), 3.19-3.11 (m, 2H), 2.91 (t, *J* = 5.4 Hz, 1H), 2.75 (dt, *J* = 10.9, 5.9 Hz, 1H), 2.31 (hept, *J* = 2.9 Hz, 1H), 1.39 (s, 3H), 1.26 (d, *J* = 10.2 Hz, 1H), 0.65 (s, 3H) ppm. **^{13}C NMR** (75 MHz,

$CDCl_3$): δ = 148.48, 143.19, 137.29, 119.08, 113.09, 44.98, 39.65, 39.30, 32.86, 31.55, 25.71, 21.51 ppm. **IR** (neat): $\tilde{\nu}$ = 3098 (w), 2933 (m), 2873 (w), 2245 (w), 1652 (w), 1539 (w), 1498 (s), 1467 (w), 1444 (w), 1425 (w), 1387 (w), 1372 (w), 1269 (w), 1224 (w), 1187 (w), 1133 (w), 1094 (m), 1037 (w), 1002 (w), 946 (w), 911 (m), 881 (w), 845 (m), 819 (w), 793 (w), 761 (w), 727 (s), 683 (w), 646 (w), 611 (w), 575 (w), 499 (w), 475 (w), 427 (w) cm^{-1}. **HRMS** ESI; *m/z* calcd. for $C_{12}H_{14}N_4$ $[M+Na]^+$: 237.1111, found: 237.1105. $[\alpha]_D^{23}$ = –31.8° (*c* = 1.0, $CHCl_3$)

(6*R*,8*R*)-3-(4-mesityl-1*H*-1,2,3-triazol-1-yl)-7,7-dimethyl-5,6,7,8-tetrahydro-6,8-methanoisoquinoline (S5)

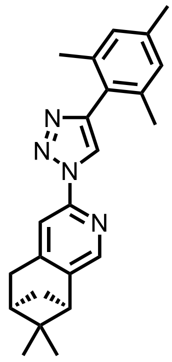

S5

Following a modified procedure from Košmrlj *et al.*,[3] a pressure tube was charged with tetrazole **5** (467 mg, 2.18 mmol, 1.00 eq) and $(CuOTf)_2 \cdot C_6H_6$ (109 mg, 0.21 mmol, 0.10 eq) and put under nitrogen atmosphere. Subsequently, the solids were dissolved in dry toluene (8.0 mL) and mesityl alkyne (0.38 mL, 2.40 mmol, 1.10 eq) was added to the reaction mixture. The pressure tube was sealed tightly and heated at 100 °C for 20 h. Afterwards, the the solvent was removed under reduced pressure and the crude product was purified by column chromatography (silica, *n*-Pentane/EtOAc 15:1 → 10:1) to obtain triazole **S5** (535 mg, 1.49 mmol, 68%) as an off-white solid.

TLC (*n*-Pentane/EtOAc 15:1): R_f=0.23. **MP** 206–207 °C. **^{1}H NMR** (300 MHz, $CDCl_3$): *δ* = 8.44 (s, 1H), 8.07 (s, 1H), 8.02 (s, 1H), 6.92 (s, 2H), 3.15-3.08 (m, 2H), 2.90 (t, *J* = 5.3 Hz, 1H), 2.76 (dt, *J* = 10.5, 5.3 Hz, 1H), 2.40-2.29 (m, 4H), 2.17 (s, 6H), 1.44 (s, 3H), 1.25 (d, *J* = 9.8 Hz, 1H), 0.68 (s, 3H) ppm. **^{13}C NMR** (75 MHz, $CDCl_3$): δ = 148.44, 148.18, 145.92, 144.14, 142.97, 138.28, 137.96, 128.51 (2C), 127.11, 119.92, 113.33, 44.45, 39.96, 39.38, 33.27, 31.91, 26.02, 21.47, 21.22, 20.85 (2C) ppm. **IR** (neat): $\tilde{\nu}$ = 3363 (w), 3159 (w), 2926 (m), 2869 (w), 1607 (m), 1570 (w), 1477 (w), 1453 (s), 1425 (w), 1378 (w), 1346 (w), 1264 (w), 1225 (m), 1127 (w), 1098 (w),

1074 (w), 1032 (s), 1005 (w), 948 (w), 931 (w), 903 (w), 870 (w), 849 (m), 819 (w), 733 (w), 695 (w), 664 (w), 576 (w), 552 (w), 495 (w), 449 (w), 424 (w) cm^{-1}. **HRMS** ESI; *m/z* calcd. for $C_{23}H_{27}N_4$ $[M]^+$: 359.2230, found: 359.2216. $[\alpha]_D^{23}$ = –40.5° (*c* = 1.0, $CHCl_3$)

1-((6*R*,8*R*)-7,7-dimethyl-5,6,7,8-tetrahydro-6,8-methanoisoquinolin-3-yl)-4-mesityl-3-methyl-1*H*-1,2,3-triazol-3-ium (6)

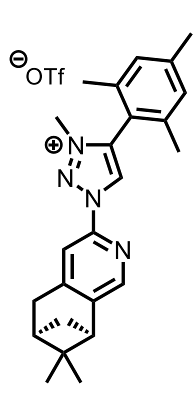

6

Following a modified procedure from Košmrlj *et al.*,[4] triazole **S5** (425 mg, 1.18 mmol, 1.00 eq) was dissolved in dry CH_2Cl_2 (5.50 mL) under nitrogen atmosphere and the reaction mixture cooled down to 0 °C. Then, MeOTf (161 mL, 1.42 mmol, 1.20 eq) was added dropwise and the reaction mixture was stirred overnight, while allowing to warm up to room temperature. The solution was then diluted with CH_2Cl_2 and filtered over a plug of silica nd the impurities were washed off with CH_2Cl_2. Then, the product was eluted with a mixture of CH_2Cl_2/MeOH 5:1. The solvent was removed under reduced pressure and the analytically pure triazolium ligand **6** (572 mg, 1.09 mmol, 93%) was obtained as an off-white solid.

TLC (CH_2Cl_2/MeOH 5:1): R_f=0.48. **MP** 174–175 °C. **^{1}H NMR** (300 MHz, $CDCl_3$): δ = 8.90 (s, 1H), 8.23 (s, 1H), 8.08 (s, 1H), 7.07 (s, 2H), 4.22 (s, 3H), 3.25-3.15 (m, 2H), 2.95 (t, *J* = 5.3 Hz, 1H), 2.77 (dt, *J* = 9.9, 5.3 Hz, 1H), 2.42-2.34 (m, 4H), 2.17 (s, 6H), 1.45 (s, 3H), 1.26 (d, *J* = 9.8 Hz, 1H), 0.67 (s, 3H) ppm. **^{13}C NMR** (75 MHz, $CDCl_3$): δ = 150.55, 147.33, 145.42, 144.67, 142.87, 142.72, 129.57 (2C), 124.98, 117.58, 115.63, 44.71, 39.74, 39.23, 38.47, 33.40, 31.49, 25.93, 21.47, 21.44, 20.26 (2C) ppm. **^{19}F NMR** (282 MHz, $CDCl_3$): δ = –78.34 (s, $^-OSO_2CF_3$) ppm. **IR** (neat): $\tilde{\nu}$ = 3481 (w), 2928 (w), 1610 (w), 1580 (w), 1479 (w), 1441 (w), 1386 (w), 1342 (w), 1254 (s), 1223 (w), 1149 (s), 1098 (w), 1076 (w), 1029 (s), 948 (w), 902 (w), 853 (w), 752 (w), 635 (s), 573 (w), 516 (m), 452 (w), 436 (w) cm^{-1}. **HRMS** ESI; *m/z* calcd. for $C_{24}H_{29}N_4$ $[M – OTf]^+$: 373.2387, found: 373.2378. $[\alpha]_D^{23}$ = –32.0° (*c* = 1.0, $CHCl_3$)

Procedure for the Chiral Pinene-based nNHC-Ligand 7

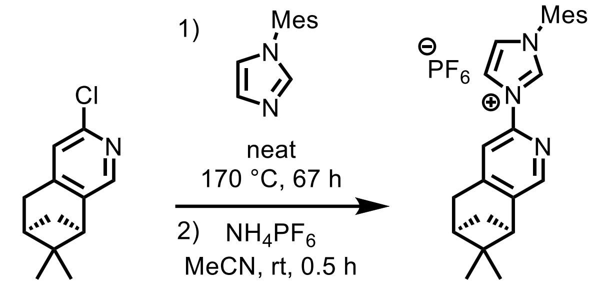

4 **90%** **7**

3-((6*R*,8*R*)-7,7-dimethyl-5,6,7,8-tetrahydro-6,8-methanoisoquinolin-3-yl)-1-mesityl-1*H*-imidazol-3-ium PF_6 (7)

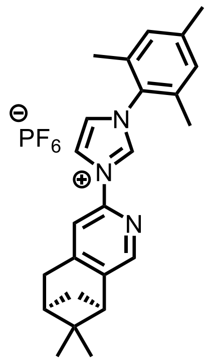

7

Under nitrogen atmosphere a pressure tube was charged with chloro-tetrahydroquinolinol **4** (200 mg, 0.96 mmol, 1.00 eq) and N-mesitylimidazole (215 mg, 1.16 mmol, 1.20 eq). The tube was immersed completely in oil, to ensure an efficient reaction, and heated to 170 °C for 90 h. Then, the residue was dissolved with CH_2Cl_2, filtered over Celite and the solvent was removed under reduced pressure. The crude product was purified by column chromatography (silica, CH_2Cl_2/MeOH 20:1 → 10:1) to obtain the imidazolium chloride ligand (358 mg, 0.90 mmol, 94%) as an off-white solid.

In the second step the chloride anion was exchanged to a hexafluorophosphate. Under air, the chloride ligand (358 mg, 0.90 mmol, 1.00 eq) and excess NH_4PF_6 (740 mg, 4.54 mmol, 5.00 eq) were stirred in MeCN (5.75 mL) for at least 1 h. Then, the solvent was evaporated under reduced pressure and the residue was redissolved in CH_2Cl_2 to precipitate the ammonium salts and filtrated over a short plug of Celite to obtain the analytical pure ligand **7** (429 mg, 0.85 mmol, 94%) as an off-white solid.

TLC (CH_2Cl_2/MeOH 20:1): R_f=0.24 (for **7**-Cl) and R_f=0.75 (for **7**-PF_6). **MP** 179–180 °C. **^{1}H NMR** (500 MHz, $CDCl_3$): δ = 9.22 (t, J = 1.6 Hz, 1H), 8.45 (t, J = 1.8 Hz, 1H), 8.03 (s, 1H), 7.84 (s, 1H), 7.43 (t, J = 1.8 Hz, 1H), 7.05 (s, 2H), 3.20-3.12 (m, 2H), 2.91 (t, J = 5.5 Hz, 1H), 2.73 (dt, J = 10.0, 5.7 Hz, 1H), 2.35 (s, 3H), 2.33 (hept, J = 1.8 Hz, 1H), 2.10 (s, 6H), 1.42 (s, 3H), 1.20 (d, J = 9.9 Hz, 1H), 0.64 (s, 3H) ppm. **^{13}C NMR** (75 MHz, $CDCl_3$): δ = 150.71, 145.60, 144.83, 144.27, 141.77, 134.20, 133.15, 130.59, 130.04 (2C), 125.03, 120.63, 113.91, 44.45, 39.69, 39.13, 33.12, 31.53, 25.89, 21.41, 21.20, 17.29 (2C) ppm. **IR** (neat): $\tilde{\nu}$ = 3156 (w), 2933 (w), 2269 (w), 1609 (w), 1540 (w), 1483 (w), 1418 (w), 1386 (w), 1369 (w), 1342 (w), 1253 (w), 1216 (w), 1153 (w), 1133 (w), 1097 (w), 1057 (w), 1031 (w), 989 (w), 907 (m), 833 (s), 724 (s), 669 (w), 648 (w), 577 (w), 556 (s), 500 (w), 446 (w), 428 (w) cm^{-1}. **HRMS** ESI; *m/z* calcd. for $C_{24}H_{28}N_3$ $[M - PF_6]^+$: 358.2278, found: 358.2267. **$[\alpha]_D^{23}$** = –30.1° (c = 1.0, $CHCl_3$)

3. Iron Complex Synthesis

General Procedure for the Complexation of the triazole ligands

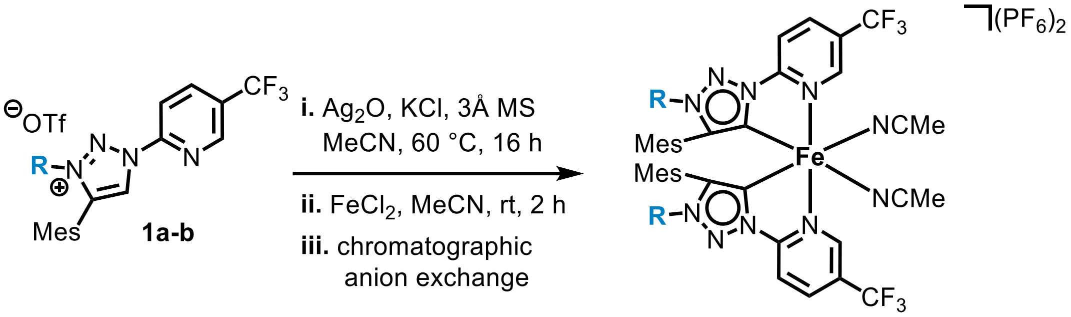

rac-**Fe1**: R = Me; 81% yield
rac-**Fe2**: R = Ph; 66% yield

In a SCHLENK tube 3 Å molecular sieves (1.0 g/mol of **1a-b**) were heated to 250 °C under vacuum for 30 min. After cooling down to room temperature, triazolium ligand **1a-b** (2.00 eq), Ag_2O (7.00 eq) and KCl (10.0 eq) were added and the reaction mixture was put under nitrogen atmosphere. Then, dry and degassed MeCN (0.025 M solution in reference to the ligand) was added and the mixture was stirred under exclusion of light at 60 °C for to generate the silver carbene.

General Procedure [A]: one-pot

After allowing to cool down to room temperature, $FeCl_2$ (0.50 eq) was added and the mixture was stirred further for 2 h at room temperature. The grey suspension turned colorful quickly to a brown/blue like color, to becoming a deep red or purple colored solution.

Note: For *rac*-**Fe1** it could be observed, that the reaction proceeds to some extent also with $FeCl_2{\cdot}(H_2O)_4$ in the presence of excess molecular sieves.

General Procedure [B]: isolation of silver carbene

After allowing to cool down to room temperature, under exclusion of light the reaction mixture was filtered over a pad of Celite to remove the excess of silver oxide and salt residues.

After the solvent was removed under reduced pressure to obtain the crude silver carbene as an off-white solid. Under nitrogen atmosphere $FeCl_2$ (0.50 eq) and MeCN were added and the mixture was stirred for 2 h at room temperature. The reaction mixture turned colorful quickly to a brown/blue like color, to becoming a deep red or purple colored solution.

After General Procedure [**A**] **or** [**B**]

Then, the mixture was diluted with MeCN, filtered over a short plug of Celite, rinsed with MeCN and the solvents were removed under reduced pressure. To remove the remaining inorganic salts the residue was dissolved in a mixture of CH_2Cl_2/MeCN 50:1 and filtered over a short plug of Celite. The crude product was purified by column chromatography (silica, CH_2Cl_2/MeCN 5:1) with a pad of NH_4PF_6 on top, to ensure complete elution of the desired product. Afterwards, the superficial NH_4PF_6 was removed via filtration from the complex by the described method above. The complex **Fe1-2** was obtained as a deeply red or purple solid.

Note: In general, the two-step procedure (General procedure **B**) leads to higher yields, but requires one more work-up step in between. Also, we observed that longer stirring times than 2 h after the addition of the iron salt can drastically decrease the yield of the desired complex.

Synthesis of *rac*-Fe1

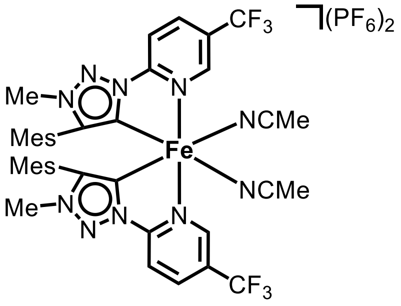

rac-**Fe1**

Following the general procedure [**A**], *rac*-**Fe1** (75.0 mg, 0.07 mmol, 81%) was obtained as a dark red solid from the corresponding ligand **1a** (82.0 mg, 0.16 mmol) and $FeCl_2{\cdot}(H_2O)_4$ (16.4 mg, 0.08 mmol) as the iron source.

^{1}H NMR (500 MHz, CD_3CN): δ = 8.63-8.62 (m, 2H), 8.03 (dd, J = 8.6, 1.8 Hz, 2H), 7.97 (d, J = 8.5 Hz, 2H), 6.81 (s, 2H), 6.71 (s, 2H), 2.24 (s, 3H), 3.81 (s, 6H), 2.23 (s, 3H), 2.03 (s, 6H), 1.27 (s, 3H) ppm. **^{13}C NMR** (125 MHz, CD_3CN): δ = 184.73, 156.95, 151.80 ($J_{C,F}$ = 4.5 Hz), 149.18, 141.77, 139.37, 138.53, 137.67 ($J_{C,F}$ = 3.2 Hz), 129.95, 129.65, 127.31 ($J_{C,F}$ = 34.1 Hz), 123.15 ($J_{C,F}$ = 272.1 Hz), 122.08, 113.74, 37.86, 21.02, 20.08, 19.37 ppm. **^{19}F NMR** (282 MHz, CD_3CN): δ = –63.02 (s, CF_3), –73.34 (d, $J_{P,F}$ = 710.6 Hz, PF_6) ppm. **IR** (neat): $\tilde{\nu}$ = 3671 (w), 3091 (w), 2925 (w), 1617 (w), 1498 (w), 1426 (w), 1399 (w), 1325 (s), 1263 (w), 1174 (w), 1142 (m), 1079 (w), 1037 (w), 1018 (w), 991 (w), 926 (w), 835 (s), 757 (w), 739 (w), 699 (w), 662 (w), 629 (w), 607 (w), 558 (m), 490 (w), 466 (w), 443 (w) cm^{-1}. **HRMS** ESI; *m/z* calcd. for $C_{37}H_{35}F_6Fe_1N_8O_2$ $[M+COO^{-}]^{+}$: 793.2132, found: 793.2103 (formiate coordinating instead of MeCN).

Synthesis of *rac*-Fe2

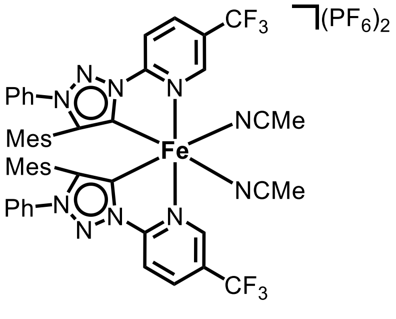

rac-**Fe2**

Following the general procedure [**B**], *rac*-**Fe2** (217 mg, 0.17 mmol, 66%) was obtained as a dark purple solid from the corresponding ligand **1b** (330 mg, 0.59 mmol) and $FeCl_2$ (33.7 mg, 0.27 mmol) as the iron source.

^{1}**H NMR** (500 MHz, CD_3CN): δ = 8.79 (s, 2H), 8.09-8.05 (m, 4H), 7.58 (t, J = 7.5 Hz, 2H), 7.47 (t, J = 7.9 Hz, 4H), 7.32 (d, J = 8.4 Hz, 4H), 6.75 (s, 2H), 6.61 (s, 2H), 2.19 (s, 6H), 2.17 (s, 6H), 1.32 (s, 6H) ppm. ^{13}**C NMR** (125 MHz, CD_3CN): δ = 184.60, 157.03, 151.99 ($J_{C,F}$ = 4.4 Hz), 149.01, 141.81, 139.69, 138.10, 137.83 ($J_{C,F}$ = 3.5 Hz), 135.29, 132.98, 131.03 (4C), 130.14, 129.60, 127.79 ($J_{C,F}$ = 34.3 Hz), 125.19, 123.42 ($J_{C,F}$ = 271.2 Hz), 122.58, 114.27, 20.98, 20.56, 19.90 ppm. ^{19}**F NMR** (282 MHz, CD_3CN): δ = –62.99 (s, CF_3), –72.94 (d, $J_{P,F}$ = 706.4 Hz, PF_6) ppm. **IR** (neat): $\tilde{\nu}$ = 3670 (w), 3087 (w), 2925 (w), 1618 (w), 1593 (w), 1490 (w), 1456 (w), 1430 (w), 1397 (w), 1322 (s), 1277 (w), 1175 (w), 1144 (m), 1127 (w), 1085 (w), 1073 (w), 1040 (w), 992 (w), 924 (w), 833 (s), 772 (w), 755 (w), 740 (w), 725 (w), 691 (w), 641 (w), 558 (m), 523 (w), 447 (w) cm^{-1}. **HRMS** ESI; *m/z* calcd. for $C_{47}H_{39}F_6Fe_1N_8O_2$ [M+COO^-]$^+$: 917.2445, found: 917.2421 (formiate coordinating instead of MeCN).

Exemplary 2D NMR Study of **Fe2**:

Signal assignment was fulfilled by using the standard experiments ^{1}H, ^{13}C, DQF-COSY, HSQC, HMBC and NOESY. ^{1}H and ^{13}C NMR spectra labeled with assignments are given in Figures **S3**-**S4** and the 2D spectra shown in **S5**-**S8**. The NOE interactions observed are given in Table **S1**.

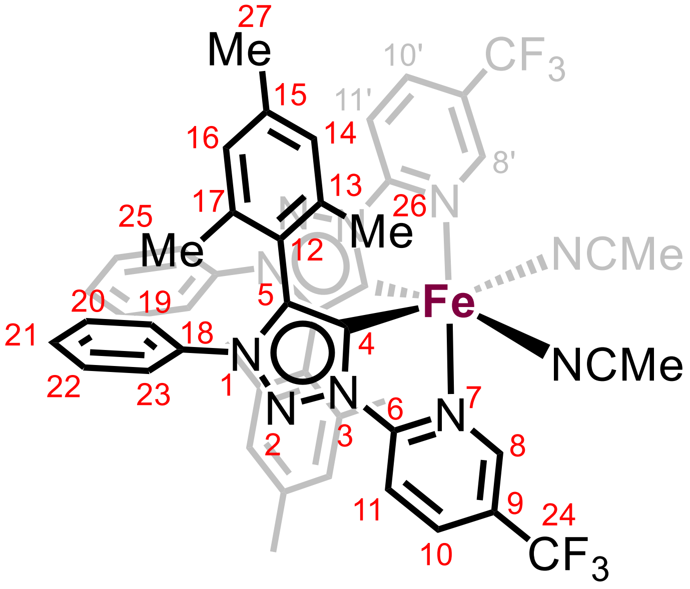

Table S1. Observed NOE interactions for **Fe2**.

NOEs between		
**Mes – Pyridine'**	**Mes – Phenyl**	**Pyridine – Phenyl**
25 – 10', 11'<br>26 – 8'<br>27 – 10', 11'<br>14 – 8', 10', 11'<br>16 – 10', 11'	25 – 19, 23<br>26 – 19, 23	10, 11 – 19, 23

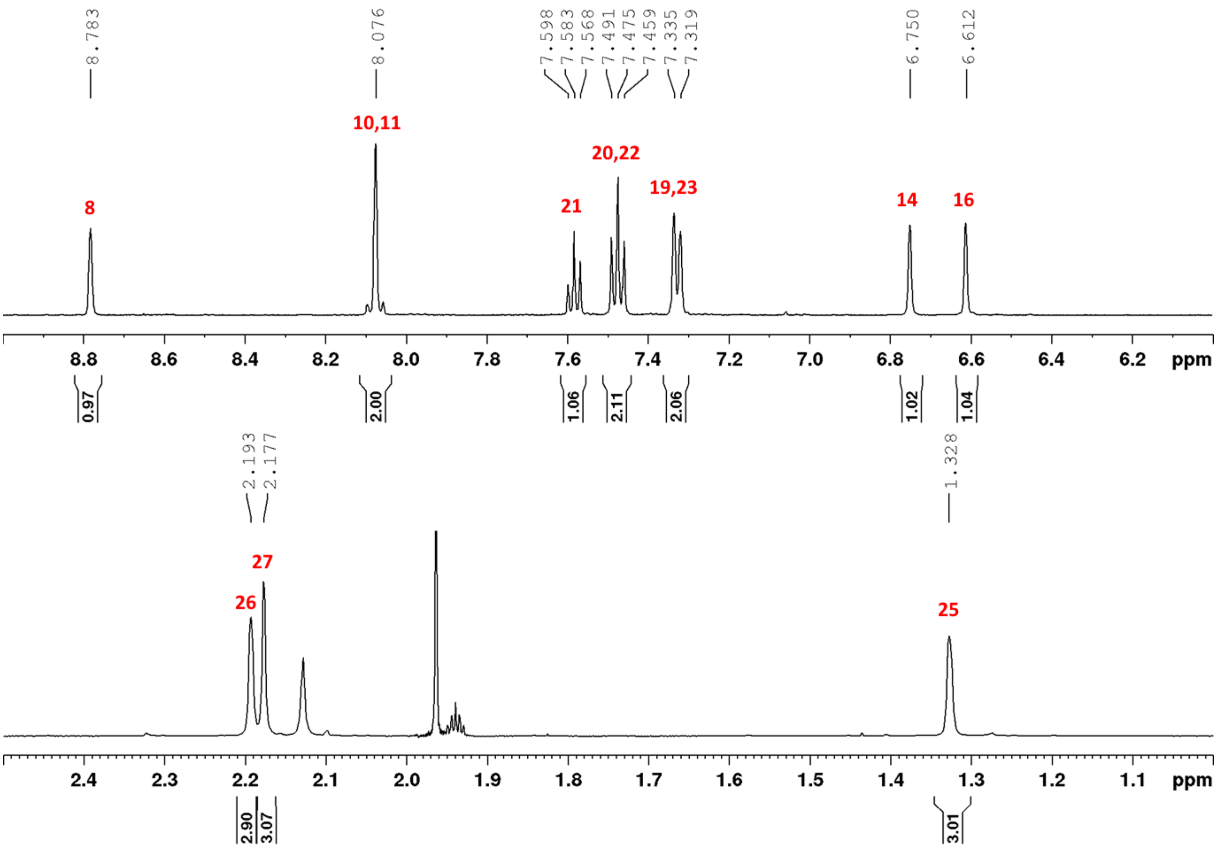

Figure S1. ^{1}H-NMR spectrum of iron complex **Fe2** with assignment (500 MHz, CD_3CN, 300 K).

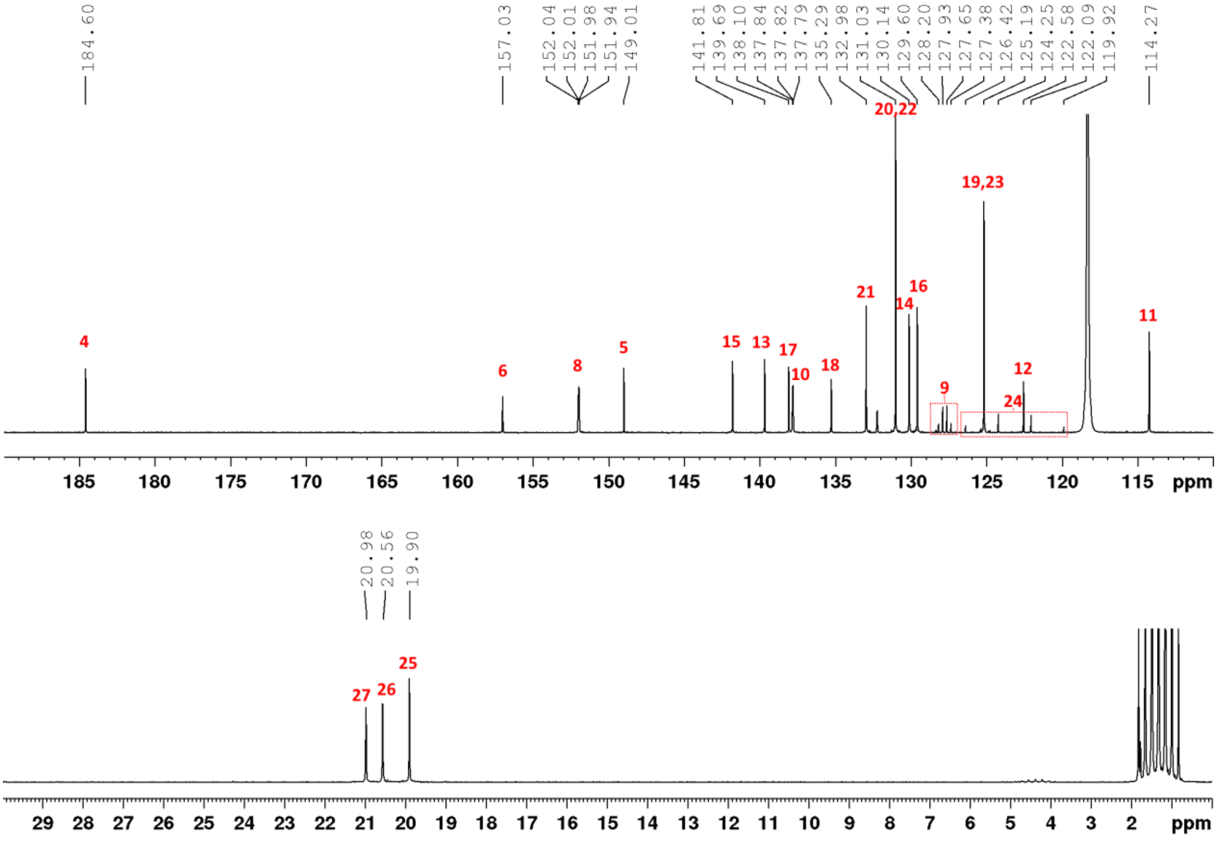

Figure S2. ^{13}C-NMR spectrum of iron complex **Fe2** with assignment (125 MHz, CD_3CN, 300 K).

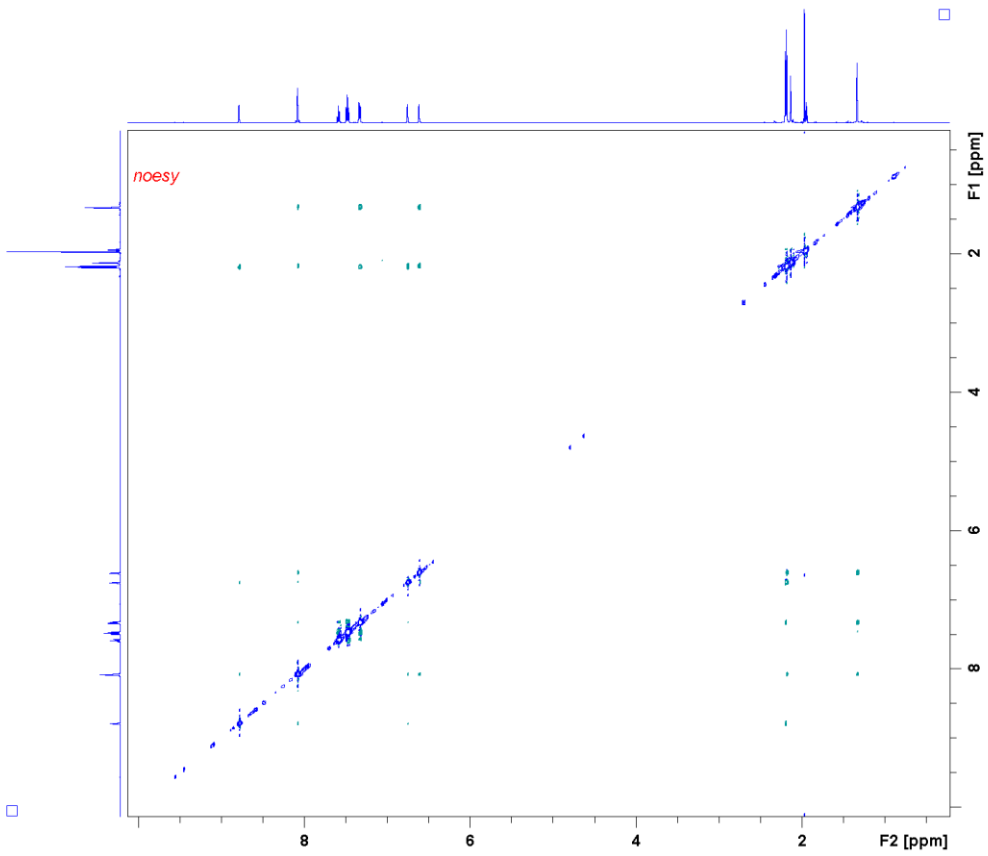

Figure S3. NOESY spectrum of iron complex **Fe2** in CD_3CN at 300 K.

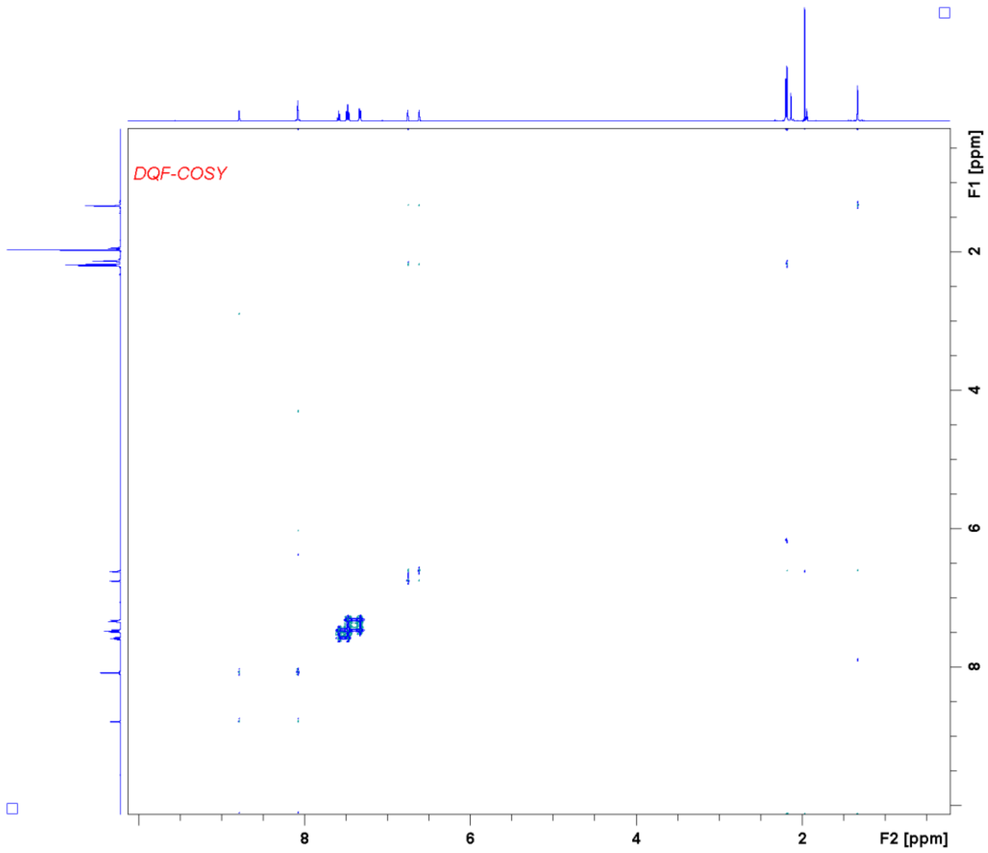

Figure S4. DQF-COSY spectrum of iron complex **Fe2** in CD_3CN at 300 K.

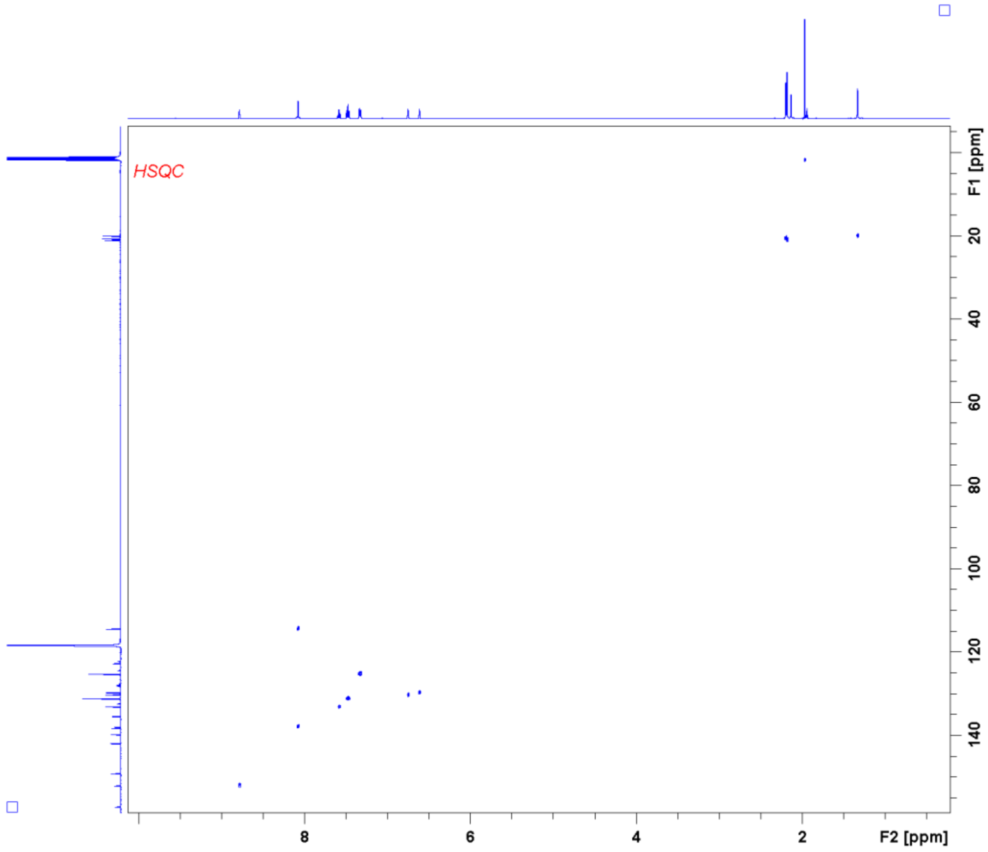

Figure S5. HSQC spectrum of iron complex **Fe2** in CD_3CN at 300 K.

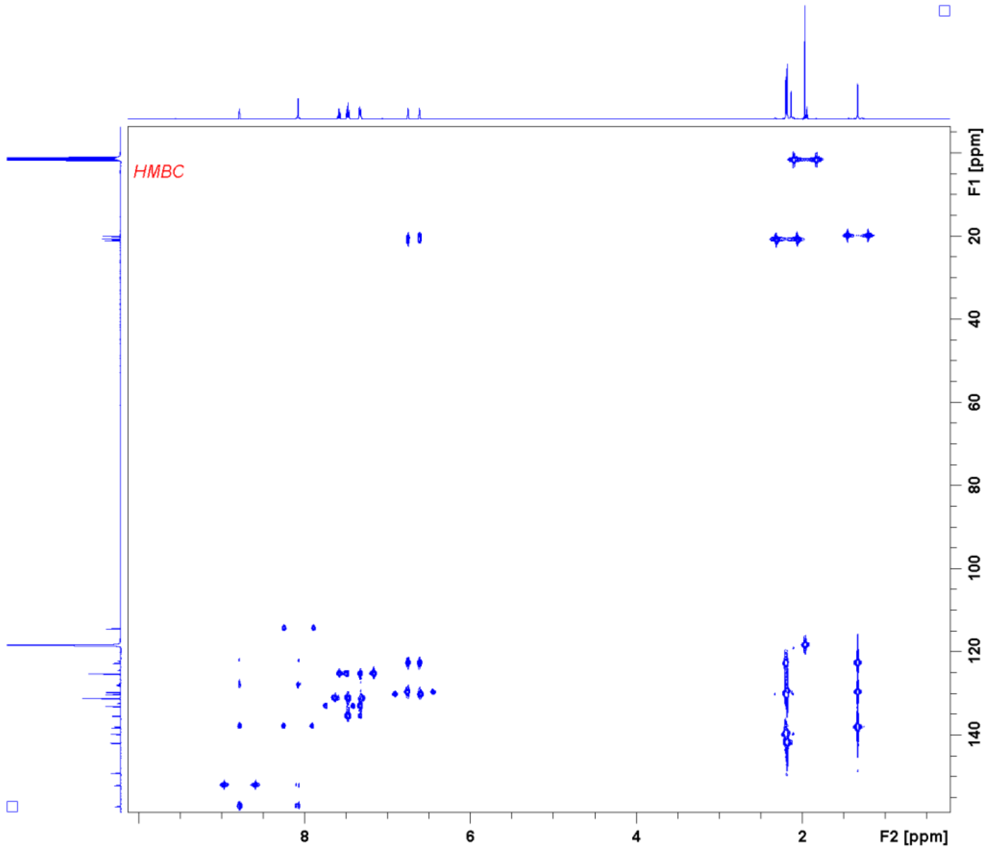

Figure S6. HMBC spectrum of iron complex **Fe2** in CD_3CN at 300 K.

Synthesis of Λ-Fepin1

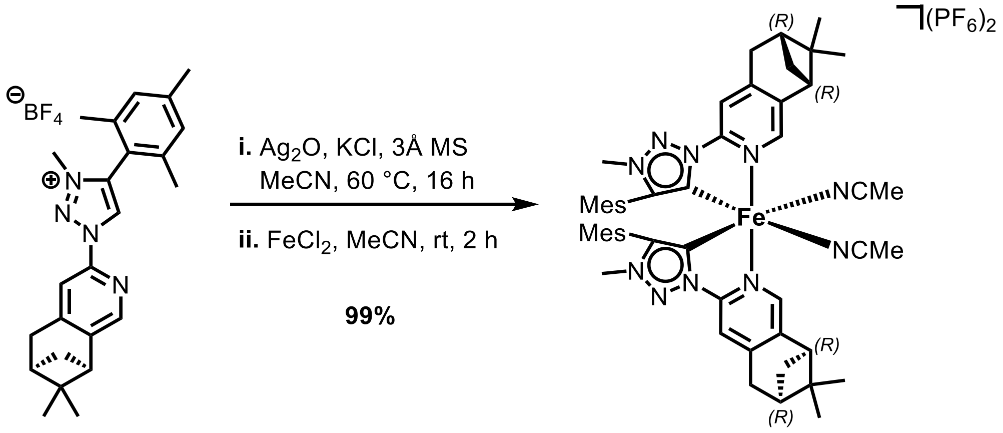

6 Λ-**Fepin1**

In a SCHLENK tube 3 Å molecular sieves (217 mg, 1.0 g/mol of **6**) were heated to 250 °C under vacuum for 30 min. After cooling down to room temperature, triazolium ligand **6-BF4** (100 mg, 0.22 mmol, 2.00 eq), Ag_2O (176 mg, 0.76 mmol, 7.00 eq) and KCl (162 mg, 2.17 mmol, 10.0 eq) were added and the reaction mixture was put under nitrogen atmosphere. Then, dry and degassed MeCN (8.00 mL) was added and the mixture was stirred under exclusion of light at 60 °C for to generate the silver carbene. After allowing to cool down to room temperature, $FeCl_2$ (13.5 mg, 0.11 mmol, 1.00 eq) was added and the mixture was stirred further for 2 h at room temperature. Then, the mixture was diluted with MeCN, filtered over a short plug of Celite, rinsed with MeCN and the solvents were removed under reduced pressure. To remove the remaining inorganic salts the residue was dissolved in a mixture of CH_2Cl_2/MeCN 50:1 and filtered over a short plug of Celite. The crude product was purified by column chromatography (silica, CH_2Cl_2/MeCN 2:1) with a pad of NH_4BF_4 on top, to ensure complete elution of the desired product (*caution: column needs to be fast! Complex is not long stable on silica*). Afterwards, the superficial NH_4BF_4 was removed via filtration from the complex by the described method above. Λ-**Fepin1** (114 mg, 0.11 mmol, 99%) was obtained as a deeply red solid.

Note: In this synthesis, it showed to be beneficial starting from the BF_4-salted ligand, as this ensured a clean conversion to the desired complex and easier purification on the silica column. The counterion of the complex can be after easily exchanged with PF_6 by just stirring the complex with an excess of NH_4PF_6 (at least 5.00 eq) in MeCN for about 1 h. The excess salts can be then removed via the method described above.

^{1}H NMR (500 MHz, CD_3CN): δ = 7.91 (s, 2H), 7.58 (s, 2H), 6.76 (s, 4H), 3.71 (s, 6H), 3.08-2.90 (m, 4H), 2.87-2.82 (m, 2H), 2.72 (dt, J = 10.6, 5.3 Hz, 2H), 2.28 (hept, J = 2.8 Hz, 2H), 2.22 (s, 6H), 2.19-2.12 (m, 2H), 1.98 (s, 6H), 1.50 (s, 6H), 1.40 (s, 6H), 1.30 (s, 6H), 1.08 (d, J = 9.7 Hz, 2H), 0.52 (s, 6H) ppm. **^{13}C NMR** (125 MHz, CD_3CN): δ = 184.61 (2C), 152.81 (2C), 150.04 (2C), 149.34 (2C), 148.75 (2C), 144.52 (2C), 140.87 (2C), 139.48 (2C), 138.63 (2C), 129.71 (2C), 129.18 (2C), 123.01 (2C), 112.17 (2C), 44.89 (2C), 40.38 (2C), 39.72 (2C), 37.25 (2C), 33.73 (2C), 31.12 (2C), 25.95 (2C), 21.54 (2C), 21.28 (2C), 20.14 (2C), 19.44 (4C) ppm. **^{19}F NMR** (282 MHz, CD_3CN): δ = –151.87 (s, BF_4) ppm. **IR** (neat): $\tilde{\nu}$ = 2927 (w), 2251 (w), 1614 (w), 1488 (w), 1424 (w), 1382 (w), 1339 (w), 1309 (w), 1269 (w), 1198 (w), 1161 (w), 1134 (w), 1106 (w), 1062 (w), 1034 (w), 1018 (w), 949 (w), 918 (w), 875 (w), 835 (s), 779 (w), 746 (w), 686 (w), 649 (w), 623 (w), 603 (w), 557 (m), 458 (w) cm^{-1}. **HRMS** ESI; *m/z* calcd. for $C_{48}H_{56}Fe_1N_8$ $[M–(MeCN)_2]^{2+}$: 400.1983, found: 400.1970.

Λ-**Fepin1:** CD (CH_3CN): λ, nm (Δε, $M^{-1}cm^{-1}$) 257 (+16), 281 (+8), 300 (+14), 325 (+1), 380 (+5), 406 (–3), 435 (+13), 480 (–10), 560 (+2).

Synthesis of Λ-Fepin2

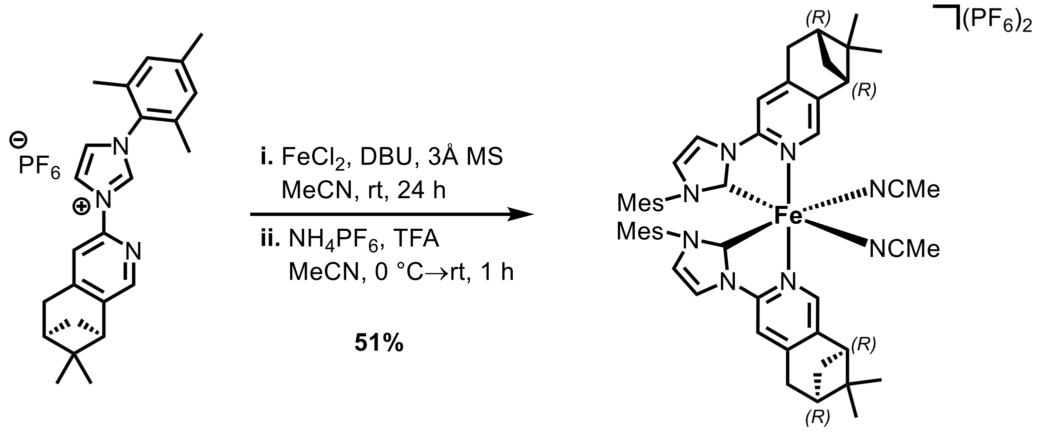

7 Λ-**Fepin2**

In a SCHLENK tube 3 Å molecular sieves (1.0 g/mol of **7**) was freshly activated at 250 °C for 30 min. After cooling down to room temperature, the tube was charged with ligand **7** (200 mg, 0.40 mmol, 2.00 equiv.), $FeCl_2$ (25.2 mg, 0.20 mmol, 1.00 eq) und put under nitrogen atmosphere. Subsequently, the solids dispersed in dry MeCN (16.0 mL) and dry DBU (89.0 µL, 0.60 mmol, 1.50 eq) was added in one portion, resulting in a color change from colorless to dark-orange. The reaction mixture was stirred at room temperature for 24 h. Then, NH_4PF_6 (194 mg, 1.20 mmol, 3.00 eq) was added to the orange dispersion and stirred for at least 0.5 h, resulting in a brighter orange suspension. The mixture was cooled to 0 °C and TFA (92.0 µL, 1.20 mmol, 3.00 eq) was added. Then it was stirred for 0.5 at 0 °C to ensure complete protonation of the remaining DBU, which probably otherwise still coordinates to the iron complex. The crude product was diluted with H_2O (10.0 mL), extracted with CH_2Cl_2 (3 x 30 mL) and washed with H_2O (3 x 10 mL) to ensure complete removal of residual salts. The combined organic phases were dried over Na_2SO_4, filtered through Celite and the solvent was removed under reduces pressure. The crude complex was purified by column chromatography on silica (CH_2Cl_2/MeCN 15:1 → 10:1). For further purification the complex is recrystallized from a minimal amount of MeCN in *i*-PrOH (If the amount of MeCN is too much, no complete

precipitation is possible. Excess of MeCN can be removed with a rotary evaporator). Λ-**Fepin2** was obtained as an orange-golden solid (115 mg, 0.10 mmol, 51%).

Note: the yield of the complexation is highly dependent on the dryness of the reagents and the removal of the coordinating DBU.

^{1}H NMR (500 MHz, CD_3CN): δ = 8.10 (d, J = 2.2 Hz, 2H), 7.64 (s, 2H), 7.28 (s, 2H), 7.06 (d, J = 2.2 Hz, 2H), 6.68 (s, 2H), 6.67 (s, 2H), 3.08-2.95 (m, 4H), 2.81 (t, J = 5.5 Hz, 2H), 2.76 (dt, J = 9.7, 5.7 Hz, 2H), 2.29 (hept, J = 2.8 Hz, 2H), 2.19 (s, 6H), 2.18-2.15 (m, 2H), 1.94 (s, 6H, underneath the CD_3CN-signal), 1.50 (s, 6H), 1.40 (s, 6H), 1.23 (d, J = 9.6 Hz, 2H), 0.49 (s, 6H) ppm. **^{13}C NMR** (125 MHz, CD_3CN): δ = 200.51 (2C), 153.87 (2C), 150.03 (2C), 148.45 (2C), 141.73 (2C), 139.95 (2C), 136.28 (2C), 134.98 (2C), 134.86 (2C), 129.96 (2C), 129.72 (2C), 128.56 (2C), 119.19 (2C), 111.02 (2C), 44.66 (2C), 40.51 (2C), 39.91 (2C), 33.80 (2C), 31.67 (2C), 25.98 (2C), 21.52 (2C), 21.12 (2C), 17.64 (4C) ppm. **^{19}F NMR** (282 MHz, CD_3CN): δ = –62.77 (s, CF_3), –72.85 (d, $J_{P,F}$ = 706.6 Hz, PF_6) ppm. **IR** (neat): $\tilde{\nu}$ = 3140 (w), 2925 (w), 2251 (w), 1651 (w), 1625 (w), 1576 (w), 1494 (m), 1446 (w), 1423 (w), 1372 (w), 1326 (w), 1300 (w), 1260 (w), 1242 (w), 1218 (w), 1165 (w), 1135 (w), 1095 (w), 1035 (w), 967 (w), 933 (w), 897 (w), 873 (w), 827 (s), 738 (w), 706 (w), 690 (w), 621 (w), 588 (w), 556 (s), 465 (w), 441 (w) cm^{-1}. **HRMS** ESI; *m/z* calcd. for $C_{52}H_{60}Fe_1N_8$ $[M]^{2+}$: 426.2140, found: 426.2131.

Note: *^{1}H- and ^{13}C-NMR still show some residual i-PrOH.*

Λ-**Fepin2:** CD (CH_3CN): λ, nm (Δε, $M^{-1}cm^{-1}$) 263 (–5), 278 (+16), 293 (–10), 363 (+31), 416 (–32), 495 (+4).

Illustration of both possible configurational isomers of **Fepin1**:

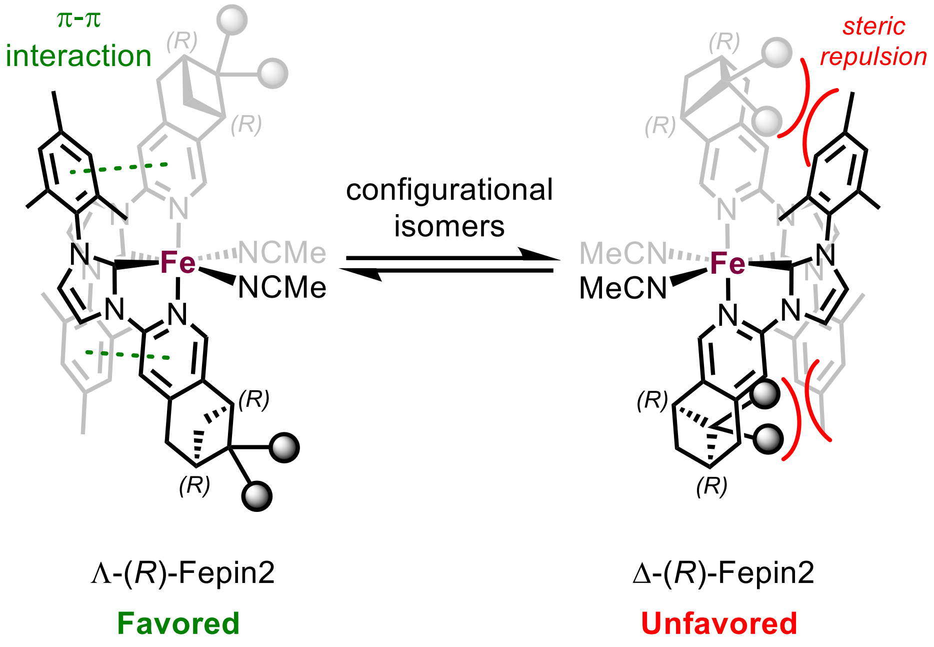

Scheme S1: Displaying both theoretically possible isomers for the (*R*)-pinene derived iron complex **Fepin2**. The methyl groups of the pinene moiety are highlighted for clearer understanding. On the left, the favored isomer is shown, which is stabilized due to π-π interligand interactions. On the right the unfavored isomer is depicted, revealing an interligand steric clash between the methyl groups of the pinene moiety and the mesityl moiety, thus preventing π-stacking.

4. Auxiliary Complex Synthesis

Synthesis of Δ-(*R*)-FeAux1

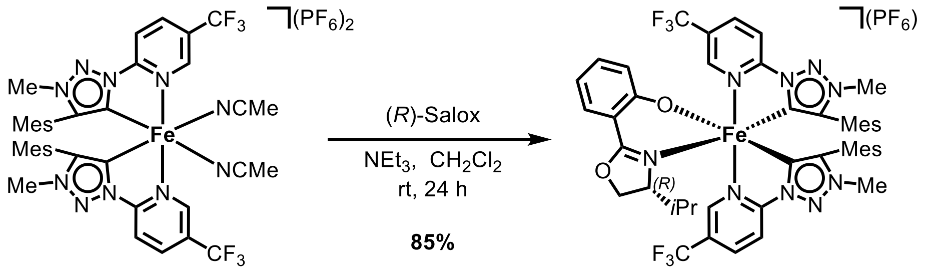

rac-**Fe1** Δ-(*R*)-**FeAux1**

Under nitrogen, a flame dried SCHLENK tube was charged with the *rac*-**Fe1** (33.6 mg, 0.03 mmol, 1.00 eq) and (*R*)-Salox (6.50 mg, 0.03 mmol, 1.05 eq). The solids were dissolved in dry CH_2Cl_2 (0.75 mL, 0.04 M) and NEt_3 (6.20 μL, 0.045 mmol, 1.50 eq) was added. The reaction was stirred at rt for 24 h. Afterwards, the mixture was concentrated under reduced pressure and the crude product was submitted to ^{19}F NMR, showing only the formation of 1 diastereomer Δ-(*R*)-**FeAux1**. The crude product was purified by aluminium oxide (activated, basic, Brockmann I) column chromatography (CH_2Cl_2/Acetone 5:1 → 4:1) and further purified by precipitation with CH_2Cl_2 in *n*-hexane (to remove formed diacetone alcohol) to afford the desired auxiliary complex Δ-(*R*)-**FeAux1** (28.0 mg, 0.026 mmol, 85%) as a deep dark purple solid.

^{1}H NMR (500 MHz, CD_2Cl_2): *δ* = 8.92 (s, 1H), 8.17 (s, 1H), 7.93 (d, *J* = 8.5 Hz, 1H), 7.90 (d, *J* = 8.5 Hz, 1H), 7.74 (dd, *J* = 8.5, 1.9 Hz, 1H), 7.68 (dd, *J* = 8.5, 1.7 Hz, 1H), 7.38 (dd, *J* = 8.0, 1.8 Hz, 1H), 6.93 (ddd, *J* = 8.6, 6.8, 1.8 Hz, 1H), 6.85 (s, 1H), 6.78 (s, 1H), 6.72 (s, 1H), 6.57 (s, 1H), 6.48 (d, *J* = 8.4 Hz, 1H), 6.21 (t, *J* = 7.2 Hz, 1H), 4.38 (dd, *J* = 9.5, 3.4 Hz, 1H), 4.25 (t, *J* = 9.3 Hz, 1H), 3.92 (dt, *J* = 8.8, 3.1 Hz, 1H), 3.83 (s, 3H), 3.80 (s, 3H), 2.25 (s, 3H), 2.20 (s, 3H), 2.19 (s, 3H), 2.12 (s, 3H), 1.31 (s, 3H), 1.03 (s, 3H), 0.53 (d, *J* = 6.6 Hz, 3H), 0.10-0.05 (m, 1H),

0.04 (d, J = 6.0 Hz, 3H) ppm. **^{13}C NMR** (125 MHz, CD_2Cl_2): δ = 192.78, 191.43, 173.30, 166.47, 157.85, 157.71, 150.90 ($J_{C,F}$ = 5.2 Hz), 150.79 ($J_{C,F}$ = 4.8 Hz), 149.62, 147.79, 141.24, 141.07, 139.70, 137.78, 137.37, 137.33, 133.72, 133.56 ($J_{C,F}$ = 3.2 Hz), 133.09 ($J_{C,F}$ = 3.5 Hz), 129.72, 129.55, 129.49, 129.24, 128.84, 125.78 ($J_{C,F}$ = 34.0 Hz), 125.32 ($J_{C,F}$ = 35.3 Hz), 123.88, 122.76 ($J_{C,F}$ = 272.8 Hz), 122.47 ($J_{C,F}$ = 271.5 Hz), 122.07, 121.84, 112.82, 112.72, 111.56, 109.80, 74.80, 66.72, 37.18, 36.78, 30.55, 21.04, 20.91, 20.45, 20.06, 19.46, 18.93, 18.48, 13.29 ppm. **^{19}F NMR** (282 MHz, CD_2Cl_2): δ = –63.02 (s, CF_3), –63.45 (s, CF_3), –73.39 (d, $J_{P,F}$ = 710.3 Hz, PF_6) ppm. **IR** (neat): $\tilde{\nu}$ = 2965 (w), 1612 (m), 1583 (w), 1538 (w), 1488 (w), 1469 (w), 1448 (w), 1422 (w), 1384 (w), 1353 (w), 1321 (s), 1265 (w), 1247 (w), 1231 (w), 1172 (w), 1139 (m), 1077 (m), 1064 (w), 1029 (w), 1017 (w), 979 (w), 917 (w), 839 (s), 754 (w), 697 (w), 656 (w), 628 (w), 606 (w), 558 (m), 489 (w), 466 (w), 446 (w), 421 (w) cm^{-1}. **HRMS** APCI; *m/z* calcd. for $C_{48}H_{48}F_6Fe_1N_9O_2$ $[M]^+$: 952.3180, found: 952.3167.

Δ-(*R*)-**FeAux1:** CD (CH_2Cl_2): λ, nm (Δε, $M^{-1}cm^{-1}$) 259 (–8), 272 (–30), 328 (–9), 350 (–13), 390 (–3), 422 (–9), 470 (+4), 535 (–9).

<u>Here an excerpt of the crude ^{19}F NMR</u>:

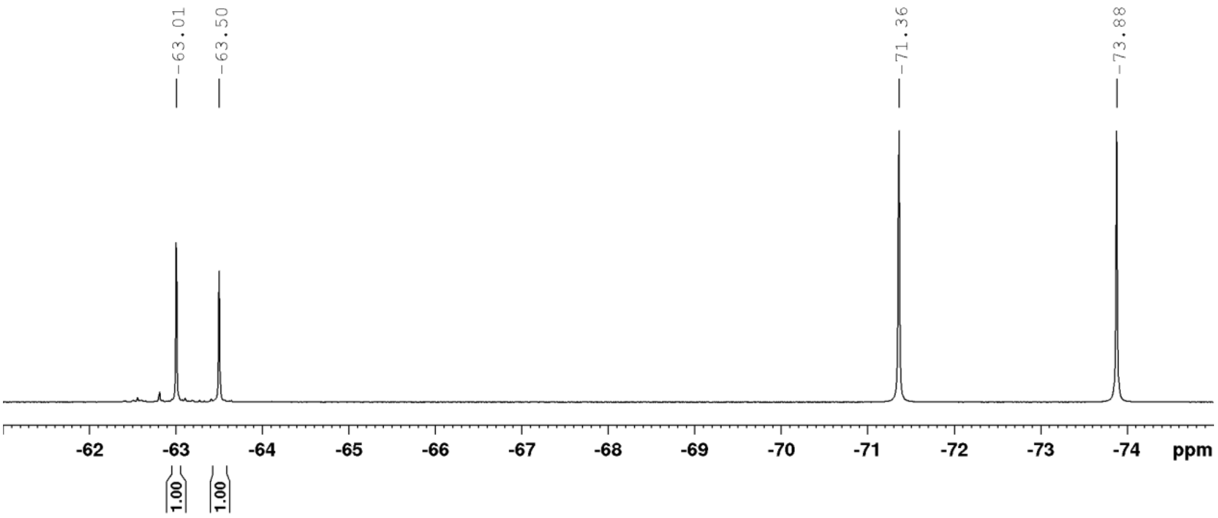

Figure S7. Crude ^{19}F-NMR spectrum of auxiliary complex synthesis (282 MHz, CD_2Cl_2, 300 K).

Synthesis of Δ-(*R*)-FeAux2

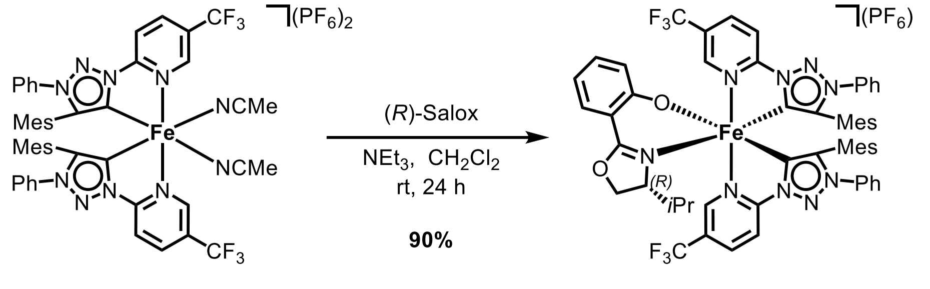

Under nitrogen, a flame dried SCHLENK tube was charged with the *rac*-**Fe2** (15.0 mg, 0.012 mmol, 1.00 eq) and (*R*)-Salox (2.72 mg, 0.013 mmol, 1.1 eq). The solids were dissolved in dry CH_2Cl_2 (0.50 mL, 0.025 M) and NEt_3 (2.34 μL, 0.017 mmol, 1.40 eq) was added. The reaction was stirred at rt for 24 h. Afterwards, the mixture was concentrated under reduced pressure and the crude product was submitted to ^{19}F NMR, showing only the formation of 1 diastereomer Δ-(*R*)-**FeAux2**. The crude product was purified by aluminium oxide (activated, basic, Brockmann I) column chromatography (CH_2Cl_2/Acetone 5:1 → 4:1) and further purified by precipitation with CH_2Cl_2 in *n*-hexane (to remove formed diacetone alcohol) to afford the desired auxiliary complex Δ-(*R*)-**FeAux2** (13.2 mg, 0.011 mmol, 90%) as a deep dark purple solid.

^{1}H NMR (500 MHz, CD_2Cl_2): *δ* = 8.98 (s, 1H), 8.25 (s, 1H), 8.04 (dd, *J* = 8.4 Hz, 3.7 Hz, 2H), 7.83 (dd, *J* = 8.6 Hz,, 1.7 Hz, 1H), 7.74 (dd, *J* = 8.5, 1.8 Hz, 1H), 7.55-7.50 (m, 2H), 7.45-7.40 (m, 5H), 7.28-7.25 (m, 4H), 6.97 (ddd, *J* = 8.6, 6.8 Hz, 1.8 Hz, 1H), 6.75 (s, 1H), 6.68 (s, 1H), 6.64 (s, 1H), 6.57 (s, 1H), 6.55 (s, 1H), 6.50 (s, 1H), 6.26 (ddd, *J* = 8.0, 6.9 Hz, 1.1 Hz, 1H), 4.43 (dd, *J* = 9.5, 3.3 Hz, 1H), 4.29 (t, *J* = 9.3 Hz, 1H), 3.99 (dt, *J* = 8.8, 3.1 Hz, 1H), 2.21 (s, 6H), 2.16 (s, 3H), 2.11 (s, 3H), 1.44 (s, 3H), 1.16 (s, 3H), 0.58 (d, *J* = 6.7 Hz, 3H), 0.18-0.11 (m, 1H), 0.08 (d, *J* = 6.5 Hz, 3H) ppm. **^{13}C NMR** (125 MHz, CD_2Cl_2): δ = 192.82, 190.95, 173.20, 166.70, 158.01,

157.92, 151.08 ($J_{C,F}$ = 5.0 Hz), 149.20, 147.45, 141.22, 141.02, 139.85, 137.55, 137.36, 137.08, 135.00, 134.97, 133.88, 133.75 ($J_{C,F}$ = 3.2 Hz), 133.43 ($J_{C,F}$ = 3.2 Hz), 132.05, 131.87, 130.35, 130.22, 129.81, 129.65, 129.54, 129.26, 128.87, 126.16 ($J_{C,F}$ = 34.3 Hz), 125.55 ($J_{C,F}$ = 34.3 Hz), 124.01, 123.95, 123.89, 122.84, 122.71 ($J_{C,F}$ = 272.6 Hz), 122.68, 122.49 ($J_{C,F}$ = 273.2 Hz), 113.34, 112.97, 112.19, 109.82, 74.90, 66.84, 30.73, 21.03, 20.92, 20.42, 19.92, 19.08, 19.01, 13.36 ppm. **^{19}F NMR** (282 MHz, CD_2Cl_2): δ = –63.04 (s, C*F*$_3$), –63.46 (s, C*F*$_3$), –73.48 (d, $J_{P,F}$ = 710.3 Hz, P*F*$_6$) ppm. **IR** (neat): $\tilde{\nu}$ = 3077 (w), 2965 (w), 2924 (w), 1611 (m), 1583 (w), 1538 (w), 1485 (m), 1469 (w), 1447 (w), 1425 (w), 1386 (w), 1353 (w), 1318 (s), 1258 (w), 1231 (w), 1172 (w), 1139 (m), 1083 (w), 1067 (w), 1027 (w), 991 (w), 977 (w), 917 (w), 839 (s), 769 (w), 755 (w), 738 (w), 690 (w), 665 (w), 640 (w), 557 (m), 522 (w) cm^{-1}. **HRMS** APCI; *m/z* calcd. for $C_{58}H_{52}F_6Fe_1N_9O_2$ [M]$^+$: 1076.3494, found: 1076.3480.

Δ-(*R*)-**FeAux2:** CD (CH_2Cl_2): λ, nm (Δε, M^{-1}cm^{-1}) 257 (+7), 274 (–19), 282 (–16), 305 (–30), 338 (–16), 392 (–4), 425 (–10), 480 (+6), 548 (–8).

Here an excerpt of the crude ^{19}F NMR:

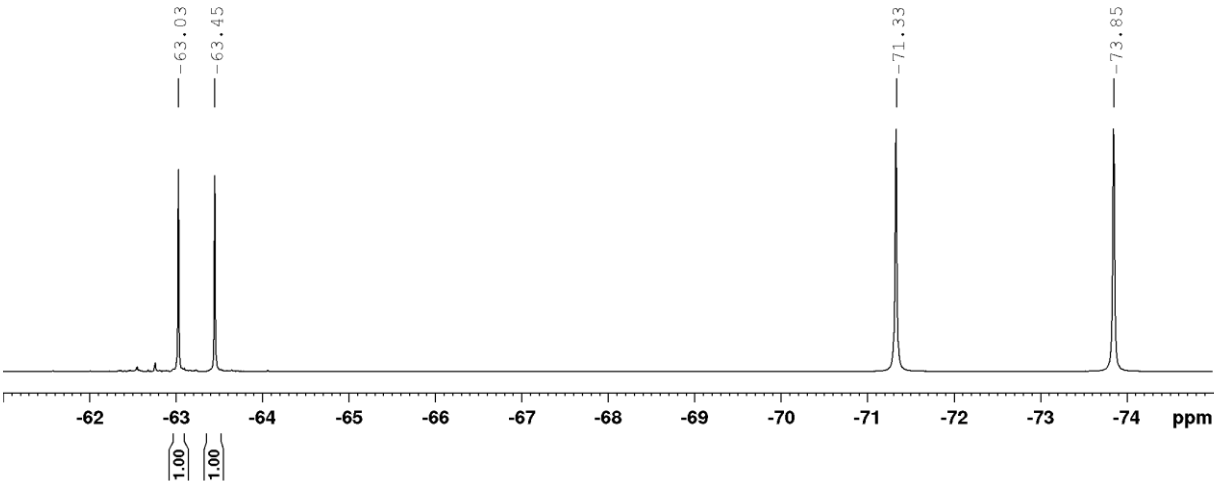

Figure S8. Crude ^{19}F-NMR spectrum of auxiliary complex synthesis (282 MHz, CD_2Cl_2, 300 K).

5. Auxiliary Cleavage

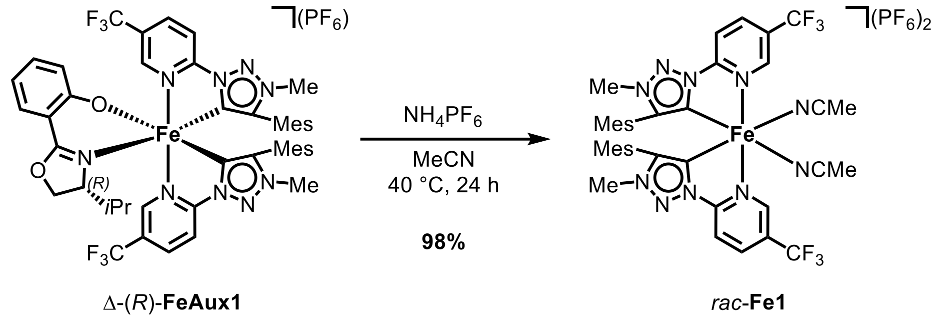

A SCHLENK flask was charged with Δ-(*R*)-**FeAux1** (6.50 mg, 0.006 mmol, 1.00 eq) and NH_4PF_6 (9.70 mg, 0.06 mmol, 10.0 eq) set under nitrogen. Then the solids were dissolved in MeCN (0.30 mL) and stirred for 24 h at 40 °C. Afterwards, the solvent was removed under reduced pressure and the residue was transferred precipitated in Et_2O to remove the free auxiliary. The complex (6.50 mg, 0.006 mmol, 98%) was obtained as a dark red solid but showed no optical activity in the CD measurement.

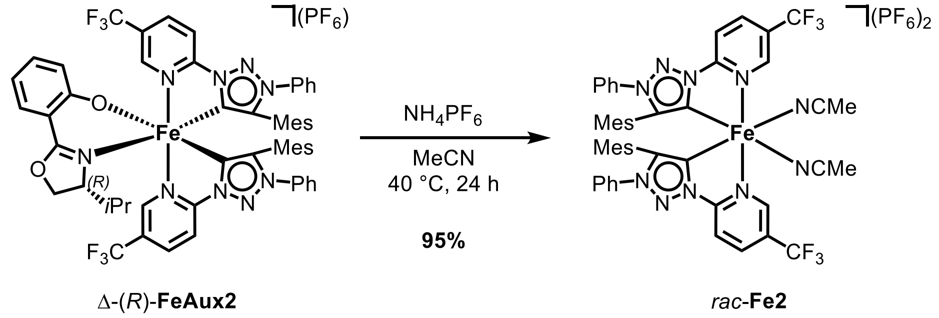

A SCHLENK flask was charged with Δ-(*R*)-**FeAux2** (9.00 mg, 0.007 mmol, 1.00 eq) and NH_4PF_6 (12.3 mg, 0.07 mmol, 10.0 eq) set under nitrogen. Then the solids were dissolved in MeCN (0.30 mL) and stirred for 24 h at 40 °C. Afterwards, the solvent was removed under reduced pressure and the residue was transferred precipitated in Et_2O to remove the free auxiliary. The complex (8.90 mg, 0.007 mmol, 95%) was obtained as a dark purple solid but showed no optical activity in the CD measurement.

6. Investigation of the Electronic Properties of MIC Ligands

With these results in hand we investigated the electronic properties of the MIC ligands **1a**-**b** via Ganters ^{77}Se NMR method.[12] As expected from the stronger σ-donating MIC ligands, the π-accepting properties dropped significantly compared to the previously investigated nNHC ligands. This is due to the lack of one nitrogen next to the carbene, which does not only have a –I-effect but also stabilized the empty p_π orbital of the carbene due to mesomeric interactions. The mesoionic character also contributes to the increased donation of the MICs. So, the MICs exhibit more of a σ-donor bond character to the metal center, while the nNHC, especially the benzimidazole NHCs show not only a σ-donating but also a π-accepting character, which contributes heavily to the configurational stability of the iron metal center.[11c] In Figure S9 the ^{77}Se values for in our group previously synthesized ligands[11c] and this works ligands are depicted (in orange) and also compared to chosen literature examples[13] (in black).

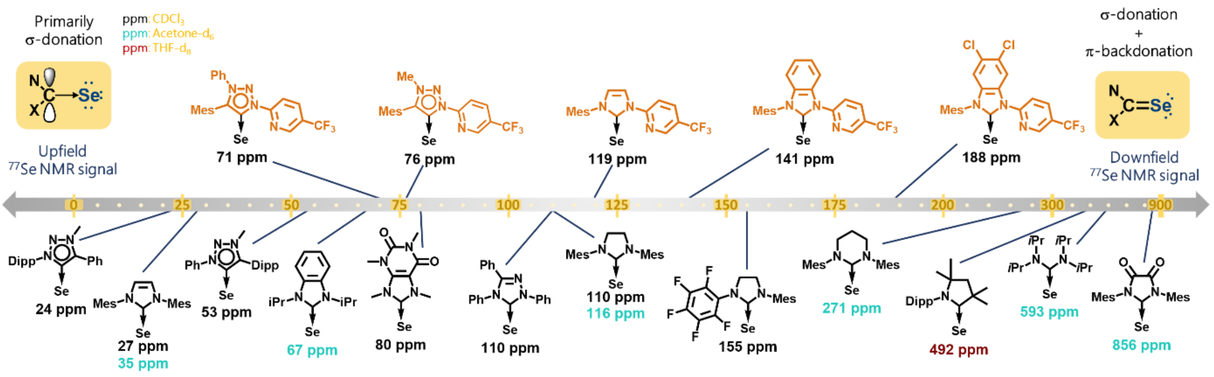

Figure S9. Characterization of the π-acceptor properties of ligands **1a**-**b** via ^{77}Se-NMR and their comparison with literature known compounds.[11c,13]

By exchanging the imidazole core with a MeTriazole the ^{77}Se signal upfield shifted from 119 ppm to 76 ppm, a difference of 43 ppm and even 65 ppm compared to the benzimidazole core. Exchanging the Methyl with a phenyl moiety leads to an even less π-accepting character for PhTriazole with 71 ppm. The ^{77}Se values for the triazole ligands **1a**-**b** confirm the drastic

decrease in their π-accepting properties, explaining the loss of configurational stability for the corresponding iron MIC complexes.

Since its first introduction over 10 years ago, the measurement of the π-accepting properties of NHCs Ganters ^{77}Se NMR method moved more and more into the spotlight due to the convenient preparation of selenoureas and its easily accessible measurement via NMR. For the determination of the σ-donating properties of NHCs Szostaks recently introduced method of using the ^{1}J(C–H) coupling constant of the carbene carbon in the starting azolium salt, joins the ranks of easily feasible methods to characterize the electronic properties of NHC ligands and is in good agreement with theoretical calculations and trends previously described in literature.[14] As expected, the ^{1}J(C–H) coupling constant in the ^{1}H NMR of the azolium salts display, that for the triazolium salts lower coupling constants ranging from 210.33 to 212.37 Hz are observed, thus showing an significant increase in σ-donor properties compared to the (benz-)imidazolium salts, which range from 223.22 to 226.79 Hz (Figure S10).

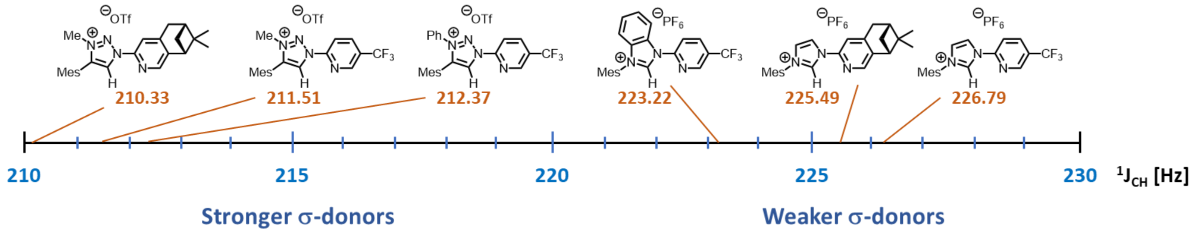

Figure S10. Characterization of the σ-donor properties of NHC ligands via Szostaks recently introduced ^{1}H NMR method using the ^{1}J(C–H) coupling constant of the carbene carbon.[14]

All ^{1}H NMR were measured at 300 K in $CDCl_3$ with 300 MHz and 128 scans.

In the following an excerpt of the ^{1}H NMR displaying the ^{1}J(C–H) coupling of each azolium salt is shown:

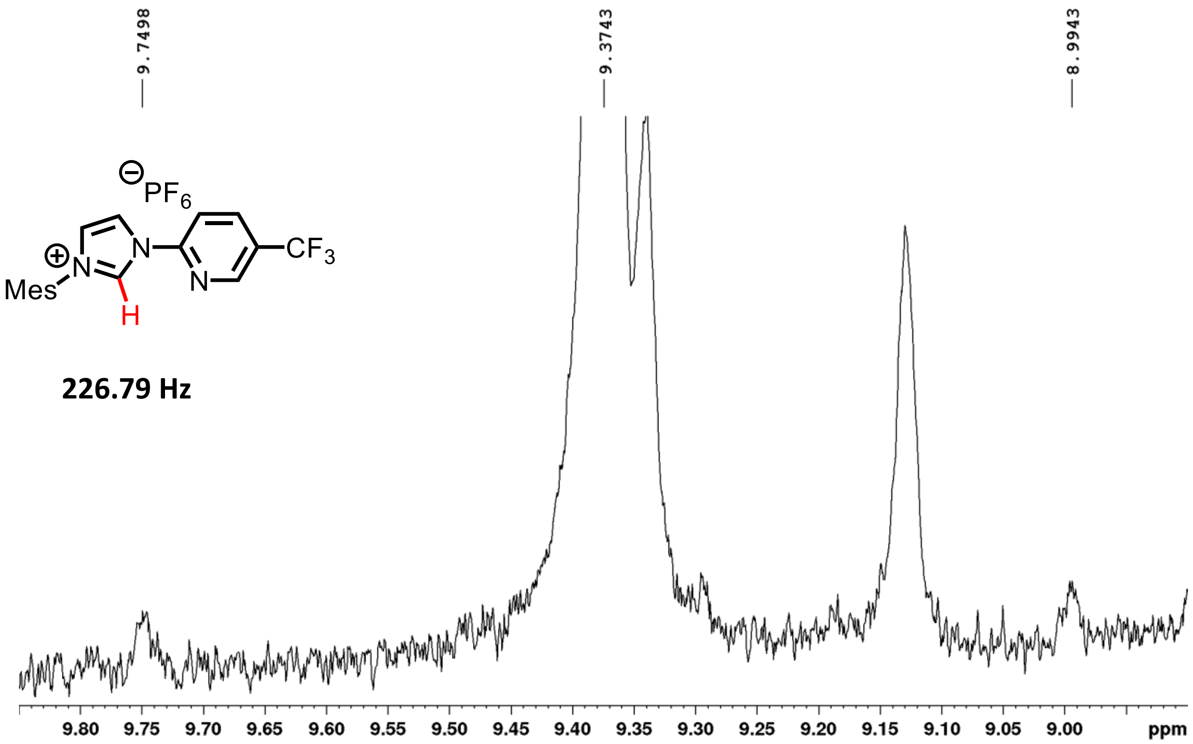
9.7498
9.3743
8.9943
PF6
CF3
N
Mes
H
226.79 Hz
9.80 9.75 9.70 9.65 9.60 9.55 9.50 9.45 9.40 9.35 9.30 9.25 9.20 9.15 9.10 9.05 9.00
ppm

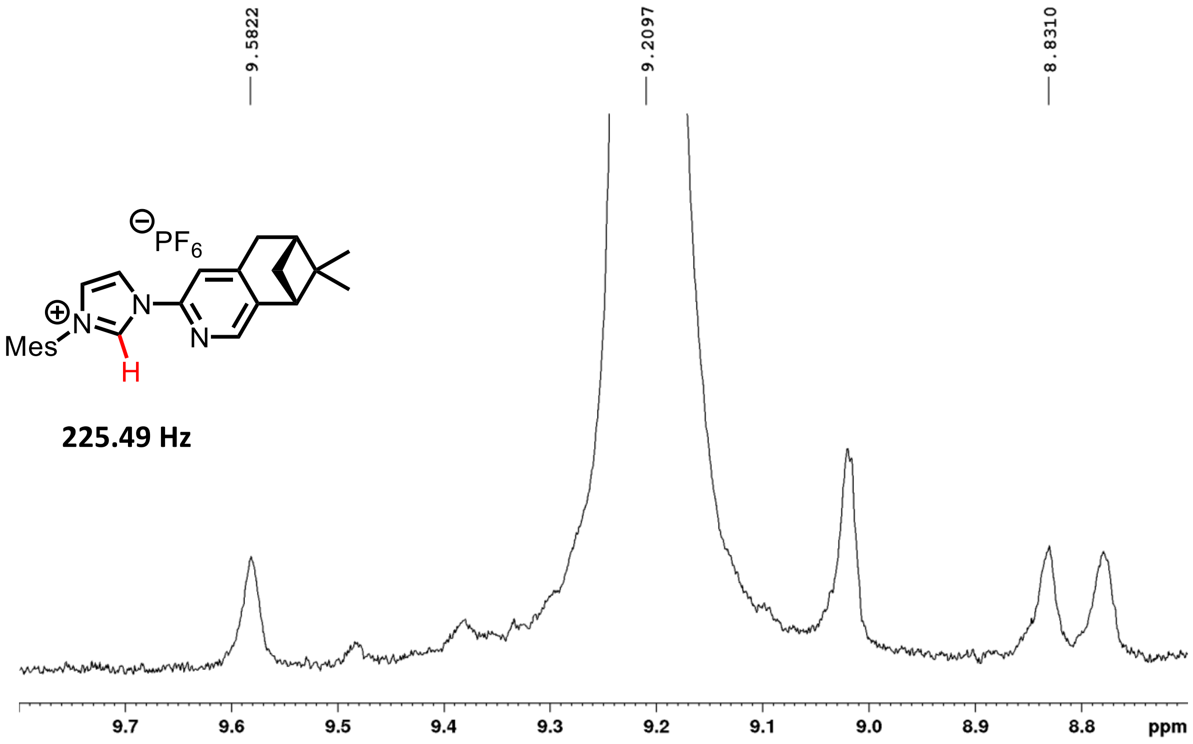
9.5822
9.2097
8.8310
PF6
N
Mes
H
225.49 Hz
9.7 9.6 9.5 9.4 9.3 9.2 9.1 9.0 8.9 8.8
ppm

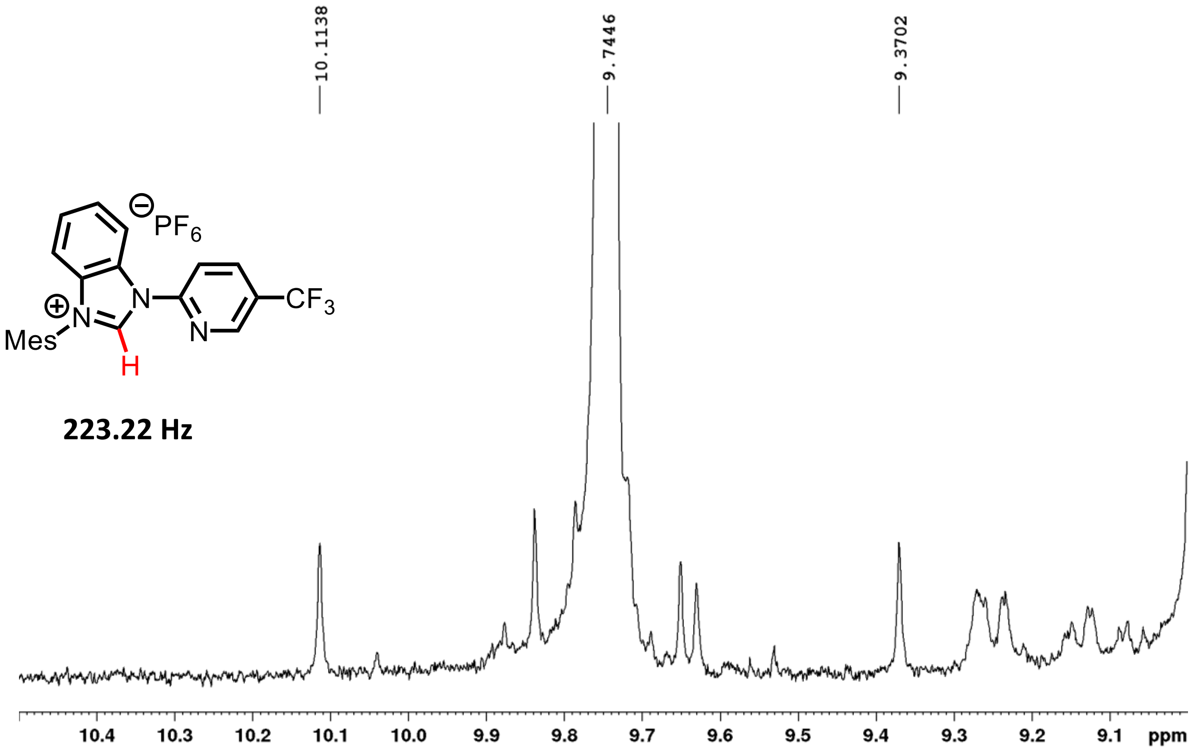

223.22 Hz

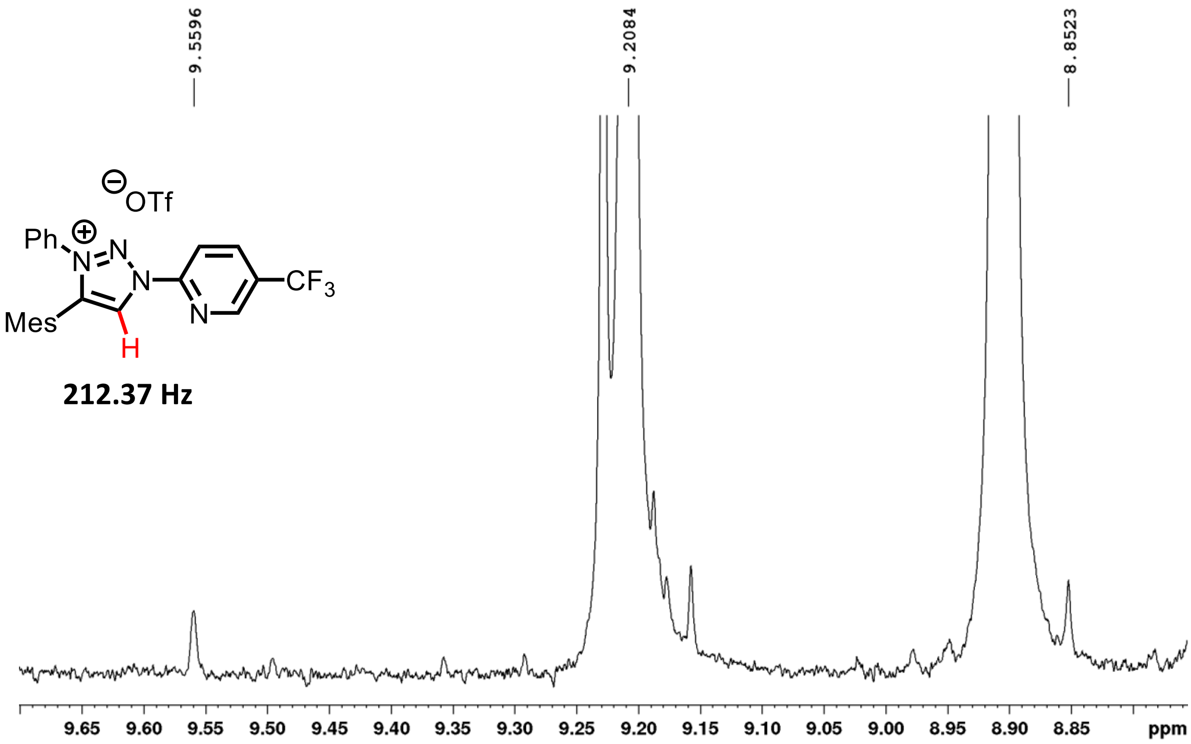

212.37 Hz

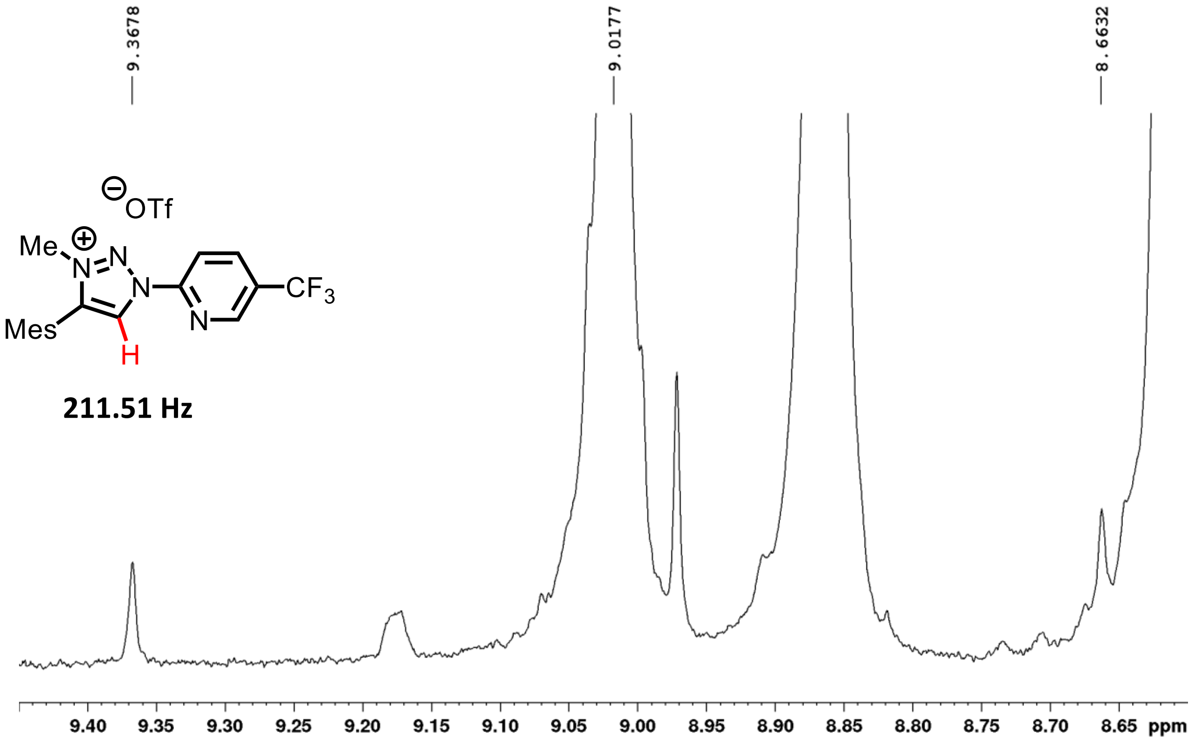
9.3678
9.0177
8.6632
OTf
Me
N
N
N
Mes
H
CF_3
211.51 Hz
9.40
9.35
9.30
9.25
9.20
9.15
9.10
9.05
9.00
8.95
8.90
8.85
8.80
8.75
8.70
8.65
ppm

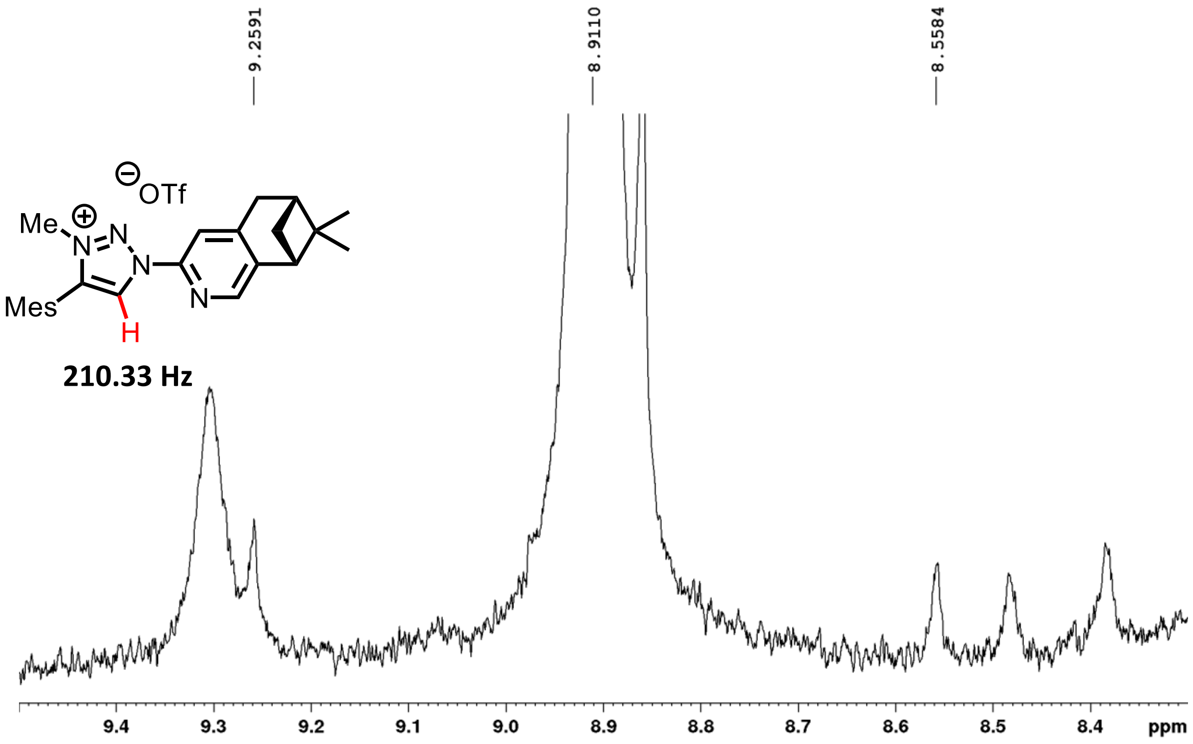
9.2591
8.9110
8.5584
OTf
Me
N
N
N
Mes
H
210.33 Hz
9.4
9.3
9.2
9.1
9.0
8.9
8.8
8.7
8.6
8.5
8.4
ppm

General Procedure for the Synthesis of Selenium-NHC products

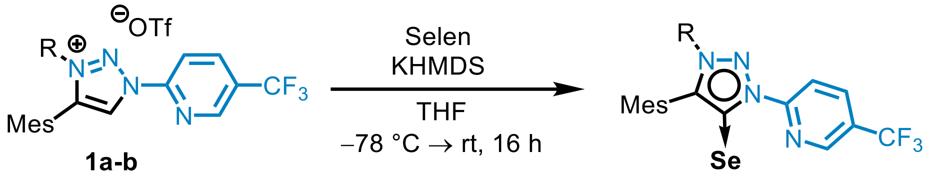

Following a literature procedure[13c], a SCHLENK tube was charged with triazolium **1a**-**b** (1.00 equiv.) and grey Selenium (3.00 equiv.) and then the flask was purged with nitrogen. Then, distilled and degassed THF (0.05 M) was added and the mixture was cooled down to –78 °C. Then NaHMDS (1 M in THF, 1.00 equiv.) was added to the reaction mixture at –78 °C and stirred for 20 h overnight, allowing it to warm up to room temperature. After addition a color change to orange/red or yellow was observed. Afterwards, the reaction mixture was first filtered over a short plug of silica with CH_2Cl_2 and after concentrating the residue filtered again over a short plug of Celite with CH_2Cl_2 to afford the desired Selenium products **Se1**-**2**. The selenium compounds **Se1**-**2** were analyzed without further purification.

1-Mesityl-3-(5-(trifluoromethyl)pyridin-2-yl)-1,3-dihydro-2H-imidazole-2-selenone (Se1)

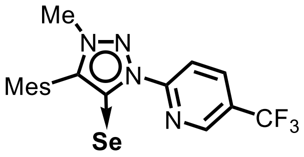

Se1

Following the general procedure, Selenium-triazole **Se1** (9.0 mg, 0.02 mmol, 53%) was obtained as an orange solid from the corresponding triazolium **1a** (20.0 mg, 0.04 mmol).

MP 78–79 °C. **^{1}H NMR** (300 MHz, $CDCl_3$): δ = 9.42 (d, J = 8.3 Hz, 1H), 8.95 (s, 1H), 8.23 (d, J = 8.5 Hz, 1H), 7.04 (s, 2H), 3.94 (s, 3H), 2.35 (s, 3H), 2.15 (s, 6H) ppm. **^{13}C NMR** (75 MHz, $CDCl_3$): δ = 151.46, 146.47 ($J_{C,F}$ = 4.1 Hz), 144.00, 141.43, 138.63, 138.10, 135.85 ($J_{C,F}$ = 3.1 Hz), 133.48, 129.28 (2C), 128.69, 128.86 ($J_{C,F}$ = 30.9 Hz), 122.99 ($J_{C,F}$ = 272.7 Hz), 120.85, 29.83, 21.49, 19.99 (2C) ppm. **^{19}F NMR** (282 MHz, $CDCl_3$): δ = –62.37 (s, CF_3) ppm. **^{77}Se NMR** (95 MHz,

$CDCl_3$): δ = 76.37 (s, C=*Se*) ppm. **IR** (neat): $\tilde{\nu}$ = 2923 (m), 2854 (w), 1726 (w), 1601 (w), 1483 (w), 1463 (w), 1387 (w), 1324 (s), 1267 (w), 1168 (w), 1128 (s), 1076 (m), 1017 (m), 938 (w), 850 (m), 766 (w), 733 (w), 700 (w), 629 (w), 600 (w), 573 (w), 545 (w), 471 (w), 442 (w). cm^{-1}. **HRMS** ESI; *m/z* calcd. for $C_{18}H_{17}F_3N_4Se_1H_1$ $[M]^+$: 427.0644, found: 427.0649.

1-Mesityl-3-(5-(trifluoromethyl)pyridin-2-yl)-1,3-dihydro-2H-imidazole-2-selenone (Se2)

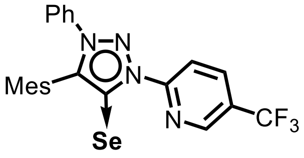

Se2

Following the general procedure, Selenium-triazole **Se2** (38.5 mg, 0.08 mmol, quant.) was obtained as a red solid from the corresponding triazolium **1b** (42.3 mg, 0.08 mmol).

MP 87–88 °C. **^{1}H NMR** (300 MHz, $CDCl_3$): δ = 9.39 (d, *J* = 8.5 Hz, 1H), 8.99 (s, 1H), 8.26 (dd, *J* = 8.4, 2.1 Hz, 1H), 7.56-7.39 (m, 5H), 6.94 (s, 2H), 2.31 (s, 3H), 2.15(s, 6H) ppm. **^{13}C NMR** (125 MHz, $CDCl_3$): δ = 151.56, 150.25, 146.65 ($J_{C,F}$ = 4.0 Hz), 142.96, 140.99, 138.45, 135.66 ($J_{C,F}$ = 3.3 Hz), 135.36, 131.38, 129.77 (2C), 129.16 (2C), 128.11 ($J_{C,F}$ = 33.4 Hz), 123.70 (2C), 123.07, 122.96 ($J_{C,F}$ = 272.8 Hz), 122.37, 121.28, 21.46, 20.27 (2C) ppm. **^{19}F NMR** (282 MHz, $CDCl_3$): δ = –62.36 (s, CF_3) ppm. **^{77}Se NMR** (95 MHz, $CDCl_3$): δ = 70.94 (s, C=*Se*) ppm. **IR** (neat): $\tilde{\nu}$ = 3034 (w), 2921 (w), 2855 (w), 1598 (m), 1517 (w), 1483 (w), 1455 (w), 1390 (w), 1323 (s), 1296 (w), 1264 (w), 1166 (w), 1133 (m), 1091 (w), 1074 (s), 1011 (m), 985 (w), 937 (w), 915 (w), 839 (m), 798 (w), 773 (w), 761 (w), 732 (s), 688 (m), 637 (w), 608 (w), 575 (w), 558 (w), 542 (w), 525 (w), 513 (w), 466 (w), 446 (w) cm^{-1}. **HRMS** ESI; *m/z* calcd. for $C_{23}H_{19}F_3N_4Se_1Na_1$ $[M+Na]^+$: 511.0620, found: 511.0620.

7. Steric Maps of Iron MIC Complexes

The limit of stereoselectivity in the reaction can be explained simply by the structure of the complexes. In Figure S11 the steric surrounding at the catalytic pockets of the stereogenic-at-metal **Fepin2** and the chiral-at-metal complexes **Fe1** and **Fe2** have been visualized utilizing the SambVca tool developed by Cavallo et al.,[15] which gives us a way to understand the difference in stereoinduction between these complexes. The C_2-symmetric complexes are split into octants to provide an intrinsic meaningful descriptor. In general, we have two occupied and two empty octants, which are mirrored for the complex enantiomers. Typically, we expect optimal stereoinduction, when the occupied octants are as full as possible and the empty octants as empty as possible.[16] If we look at the complexes **Fe3** and **Fe4**, containing only achiral ligands, we can see, that just simply changing the 5-membered-NHC core from imidazolyl to triazolyl has no significant effect on the steric surroundings.[17] For **Fe3** we got a $\Delta\%V_{bur}$ of 32.5% and for **Fe4** a $\Delta\%V_{bur}$ of 41.5%. The difference can be explained through the different orientation of the Fluorine groups of the CF_3-moiety and slightly different biting angles of the ligand. But in comparison, the steric map of the pinene based complex **Fepin2** shows that the methyl groups of the pinene moiety reach into the empty octant of the catalytic pocket, resulting in a diminished $\Delta\%V_{bur}$ of 18.2%. Thus, leading to a decreased distinction between the empty (white) and full (grey) octants, which can be conveyed to the distinction between the formation of stereoisomers of various reaction pathways.

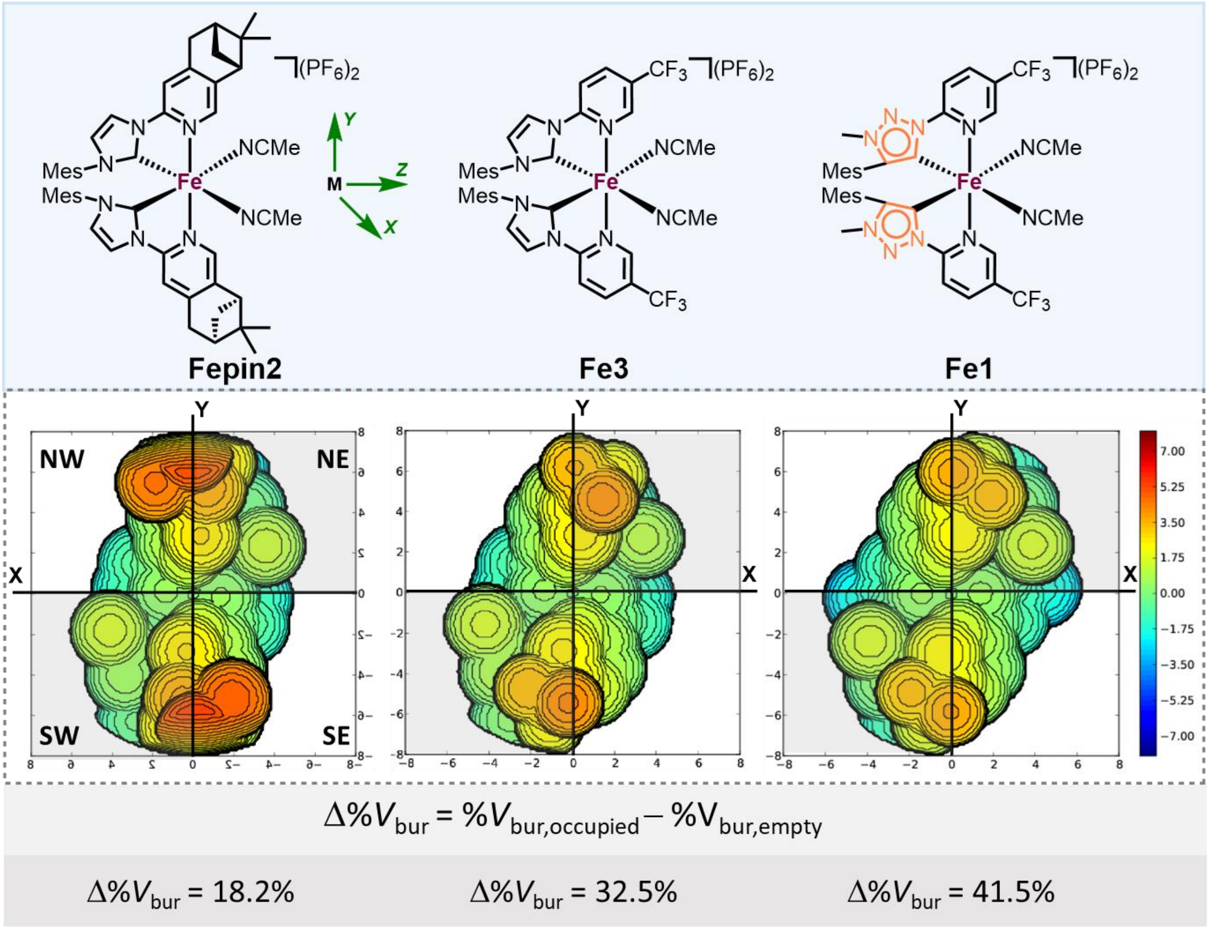

Figure S11. Steric maps based on the SCXRD structures of iron complexes **Fepin2** (left), **Fe3** (middle), and **Fe1** (right) to visualize the catalytic pocket. Calculated using SambVca.[15] The steric maps are viewed down the z-axis; the orientation of the complexes is indicated for **Fepin1** and applies to the three systems. The isocontour scheme, in Å, is shown at the bottom. The grey and white zones indicate the more- and less-hindered zones in the catalytic pocket, respectively. Comparison of the steric maps allows differences to be identified in the shapes of the catalytic pockets of the three complexes.

8. Iron-Catalyzed Ring-Closing C(sp³)-H Amidation

General Procedure

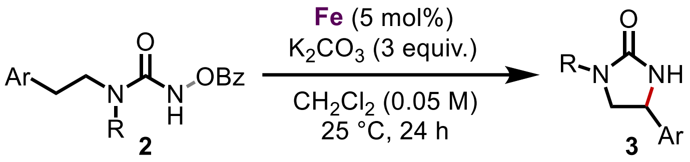

After a slightly modified procedure from MEGGERS *et al.*,[18] A SCHLENK tube was charged with substrate **2** (0.05 mmol), K_2CO_3 (20.7 mg, 0.15 mmol, 3.0 equiv.) and *rac*-**Fe1-4** or **Λ-Fepin1-2** (0.002 mmol, 5 mol%), and then the flask was purged with nitrogen. Then, distilled CH_2Cl_2 (1.0 mL, 0.05 M) was added, the flask was sealed and stirred at the indicated temperature for 24 h. Afterwards, the reaction mixture was quenched with sat. aq. NH_4Cl and extracted with EtOAc (3 x). The crude product was purified via silica gel column chromatography (*n*-pentane/EtOAc 1:2) to afford the desired cyclic urea **3** as a colorless solid.

The conversion and yield were determined by ^{1}H NMR spectroscopy of the crude product with *1,1,2,2*-tetrachloroethane as an internal standard. The enantiomeric ratio was established by HPLC analysis on a chiral stationary phase.

The spectroscopic data are in agreement with the literature.[18]

HPLC conditions:

(*S*)-**3a**: Daicel Chiralpak IG column, 250 x 4.6 mm, absorbance at 220 nm, mobile phase *n*-hexane/isopropanol = 90:10, isocratic flow, flow rate 1.0 mL/min, 30 °C, t_r (major) = 30.18 min, t_r (minor) = 33.02 min.

(*S*)-**3e**: Daicel Chiralpak IA column, 250 x 4.6 mm, absorbance at 220 nm, mobile phase *n*-hexane/isopropanol = 90:10, isocratic flow, flow rate 1.0 mL/min, 30 °C, t_r (major) = 13.19 min, t_r (minor) = 10.96 min.

(*S*)-**3f**: Daicel Chiralpak IG column, 250 x 4.6 mm, absorbance at 220 nm, mobile phase *n*-hexane/isopropanol = 90:10, isocratic flow, flow rate 0.8 mL/min, 30 °C, t_r (major) = 35.74 min, t_r (minor) = 38.28 min.

(*S*)-**3g**: Daicel Chiralpak OD-H column, 250 x 4.6 mm, absorbance at 220 nm, mobile phase *n*-hexane/isopropanol = 90:10, isocratic flow, flow rate 1.0 mL/min, 20 °C, t_r (major) = 21.39 min, t_r (minor) = 17.80 min.

(*S*)-**3h**: Daicel Chiralpak IA column, 250 x 4.6 mm, absorbance at 220 nm, mobile phase *n*-hexane/isopropanol = 90:10, isocratic flow, flow rate 1.0 mL/min, 25 °C, t_r (major) = 13.05 min, t_r (minor) = 10.91 min.

(*S*)-**3i**: Daicel Chiralpak OD-H column, 250 x 4.6 mm, absorbance at 220 nm, mobile phase *n*-hexane/isopropanol = 90:10, isocratic flow, flow rate 1.0 mL/min, 20 °C, t_r (major) = 21.08 min, t_r (minor) = 18.76 min.

9. Stability and Activity of Fepin Complexes

We evaluated the configurational stability of Λ-**Fepin1** during the reaction under standard conditions with a catalyst loading of 5 mol%. Product formation and enantioselectivity were monitored at various time points throughout the reaction (Figure S12). Consistent with typical homogeneous catalytic processes, we observed an initial exponential increase in product formation, reaching approximately 66% conversion within 7 hours, followed by a gradual and slower progression until complete conversion was achieved after 2 days. Notably, the enantiomeric excess (ee) was lower at the beginning of the reaction (64% ee at 1 hour) but increased to 84% upon full conversion.

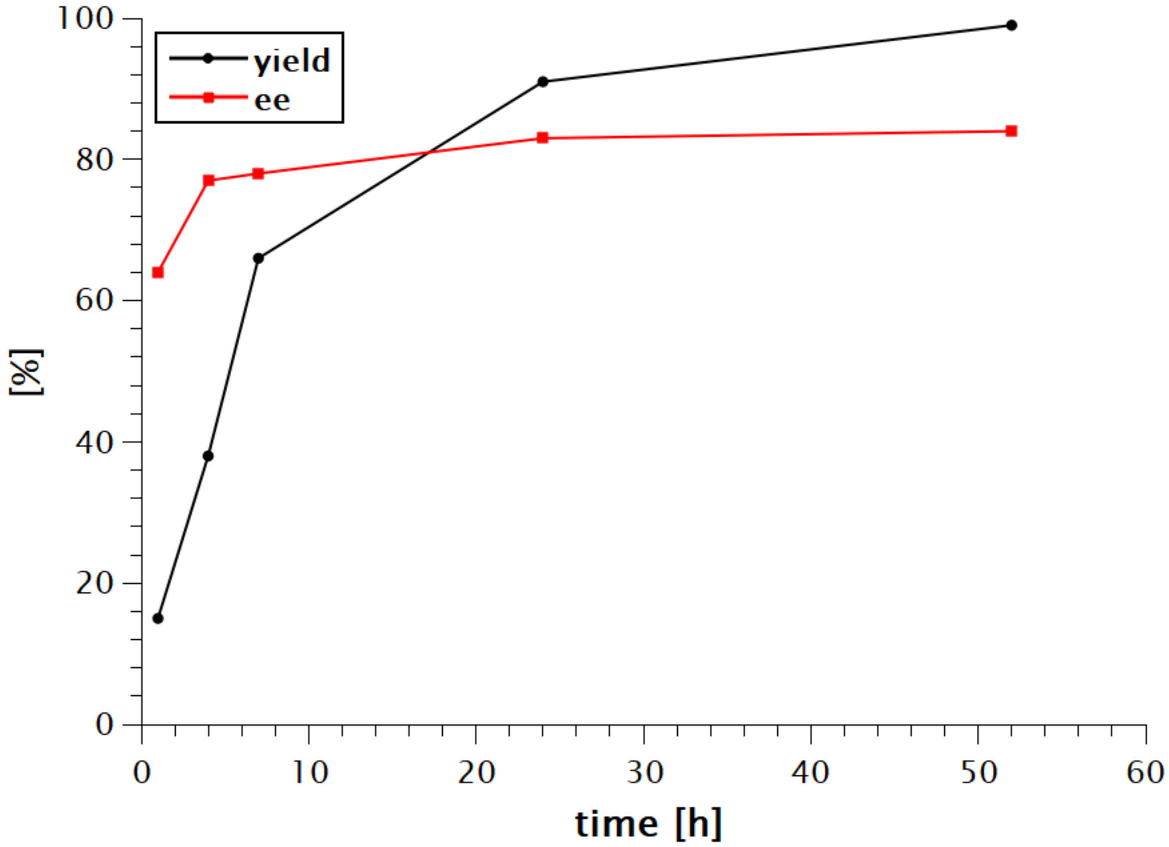

	1 h	4 h	7 h	24 h	52 h
**yield**	15%	38%	66%	91%	99%
**er**	64%	77%	78%	83%	84%

Figure S12. Kinetic ^{1}H NMR experiment. Conditions: Λ-**Fepin1** (5 mol%), urea **2i** (0.05 mmol), K_2CO_3 (0.15 mmol) and 1,3,5-trimethoxybenzene (0.016 mmol) as internal standard were dissolved in distilled CH_2Cl_2 (1.00 mL) and stirred at –10 °C.; [b] Yields and conversion were determined via ^{1}H NMR analysis with 1,3,5-trimethoxybenzene as standard.

A plausible explanation for this observation is the formation of a reactive bis-nitrene catalyst species, similar to a report by Che and co-workers.[19] The reaction conducted in the presence of air showed no conversion after 24 hours, which strongly suggests that the involvement of an iron-oxo species is unlikely. When the catalyst loading was reduced to 2 mol%, the reaction rate significantly decreased, yielding 40% product with 84% enantiomeric excess (ee) after 48 hours, and 60% yield with 85% ee after 7 days. These results indicate that the catalyst remains active over several days, continuously converting the starting material into the product while maintaining a high enantiomeric excess. No evidence of configurational instability was observed throughout the reaction.

A stability study in CD_2Cl_2 of both Λ-**Fepin1** (Figure S13) and Λ-**Fepin2** (Figure S14) revealed slow decomposition of the complexes into the free ligand. While Λ-**Fepin1** fully decomposed within a few hours, approximately 75% of Λ-**Fepin2** remained intact after 48 hours in solution. Upon the addition of approximately 4 equivalents of substrate to Λ-**Fepin2**, a rapid and pronounced color change was observed. ^{1}H NMR analysis indicated the consumption of the complex and the formation of new paramagnetic species (Figure S15), with only trace amounts of the free ligand detected, suggesting that the complex did not undergo significant decomposition.

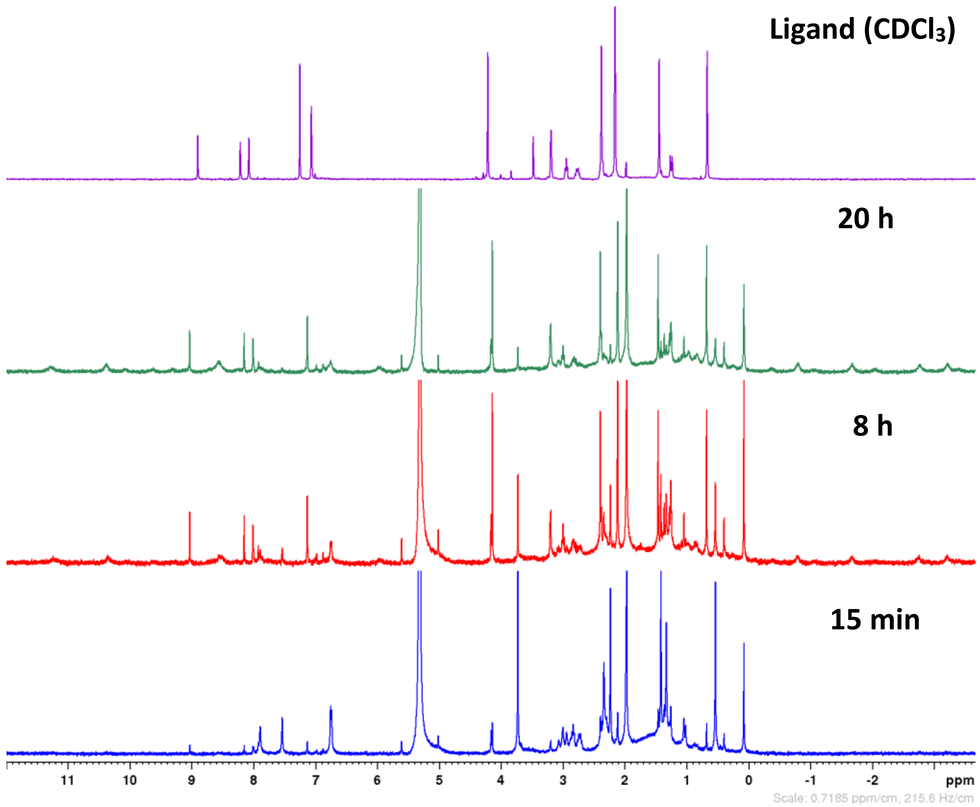

Figure S13. ^{1}H NMR of Λ-**Fepin1** in CD_2Cl_2 (300 MHz, 300 K).

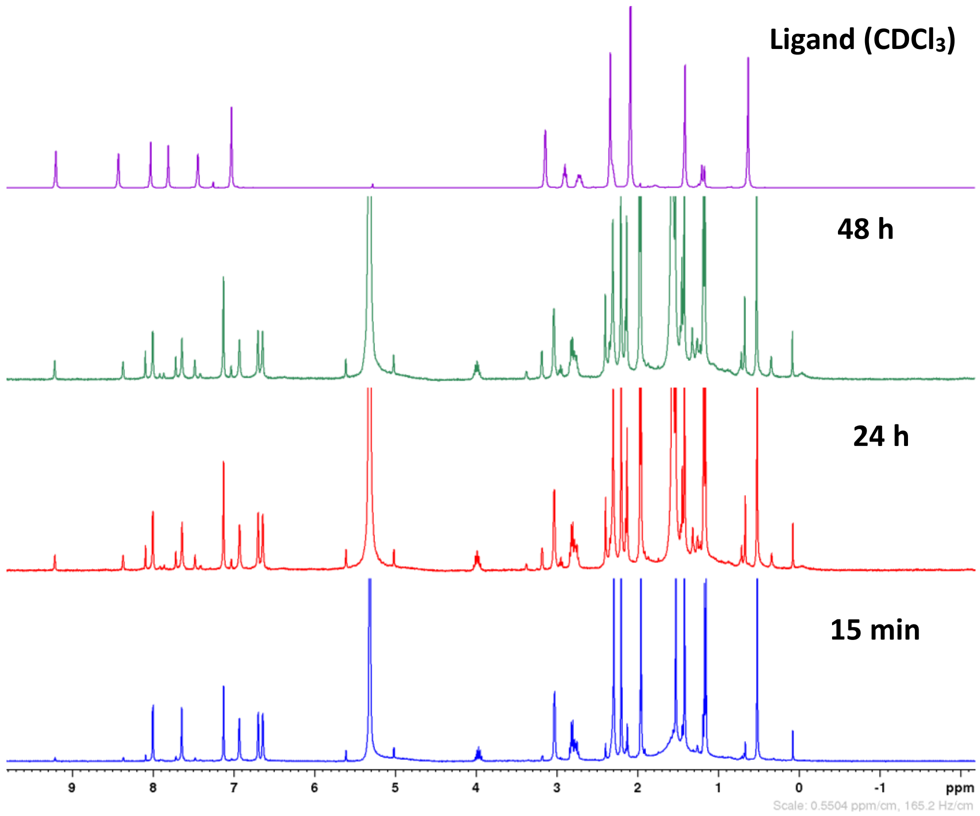

Figure S14. ^{1}H NMR of Λ-**Fepin2** in CD_2Cl_2 (300 MHz, 300 K).

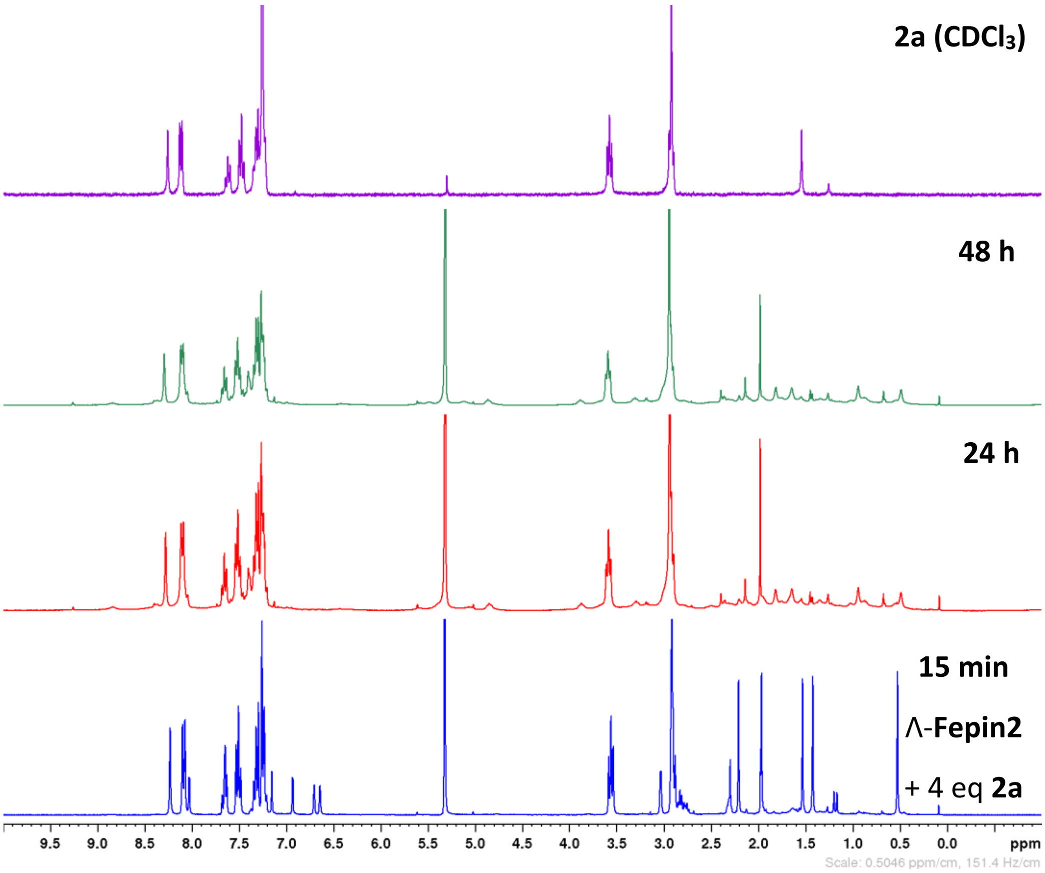

Figure S15. ^{1}H NMR of Λ-**Fepin2** in CD_2Cl_2 with 4 equivalents of substrate **2a** with a concentration of 0.050 M based on **2a** (300 MHz, 300 K).

10. NMR Spectra

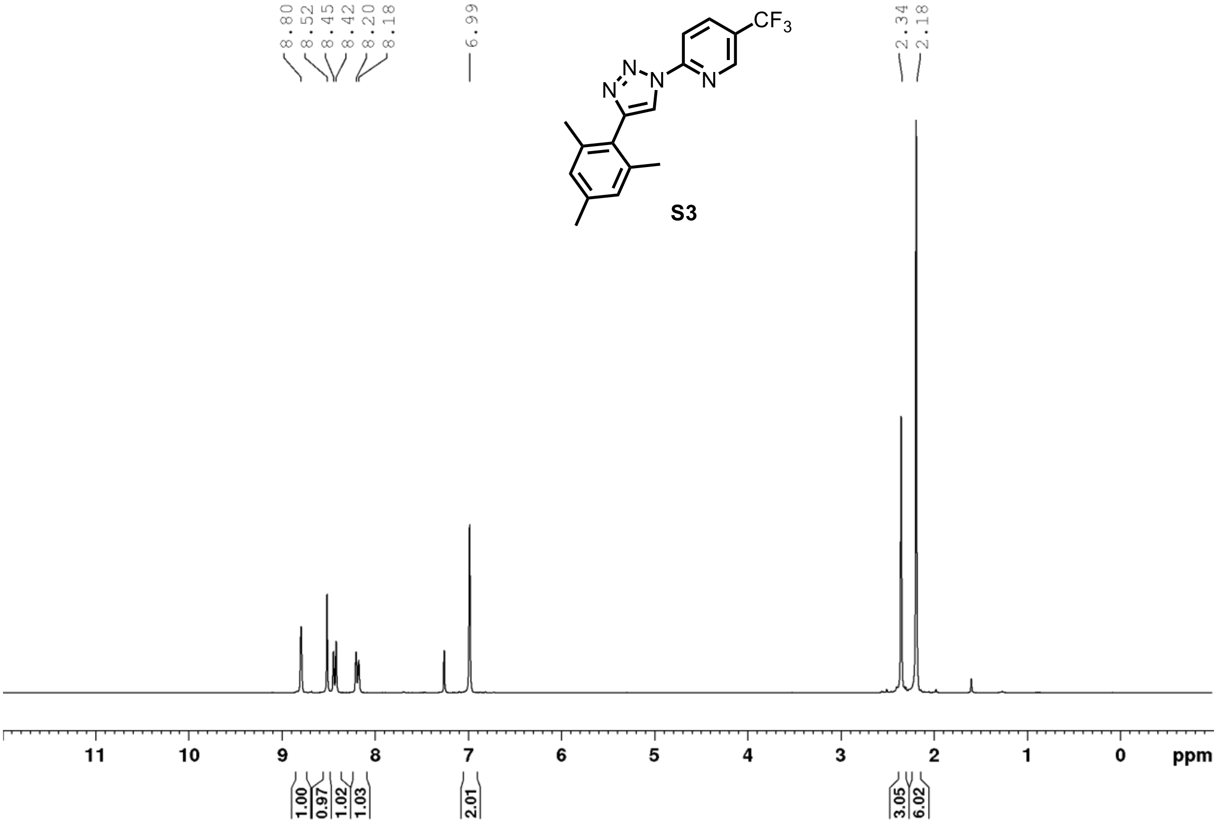

Figure S16. ^{1}H-NMR spectrum of **S3** (300 MHz, $CDCl_3$, 300 K).

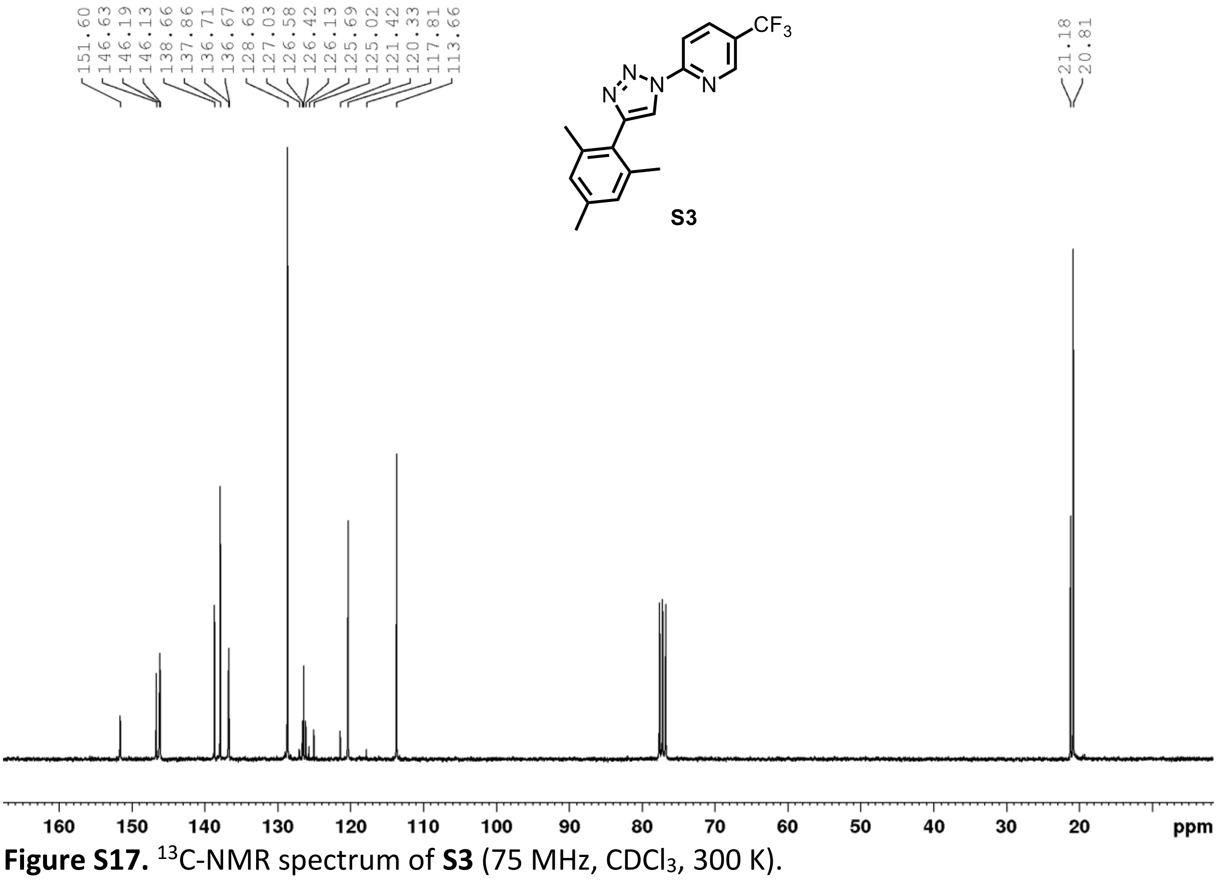

Figure S17. ^{13}C-NMR spectrum of **S3** (75 MHz, $CDCl_3$, 300 K).

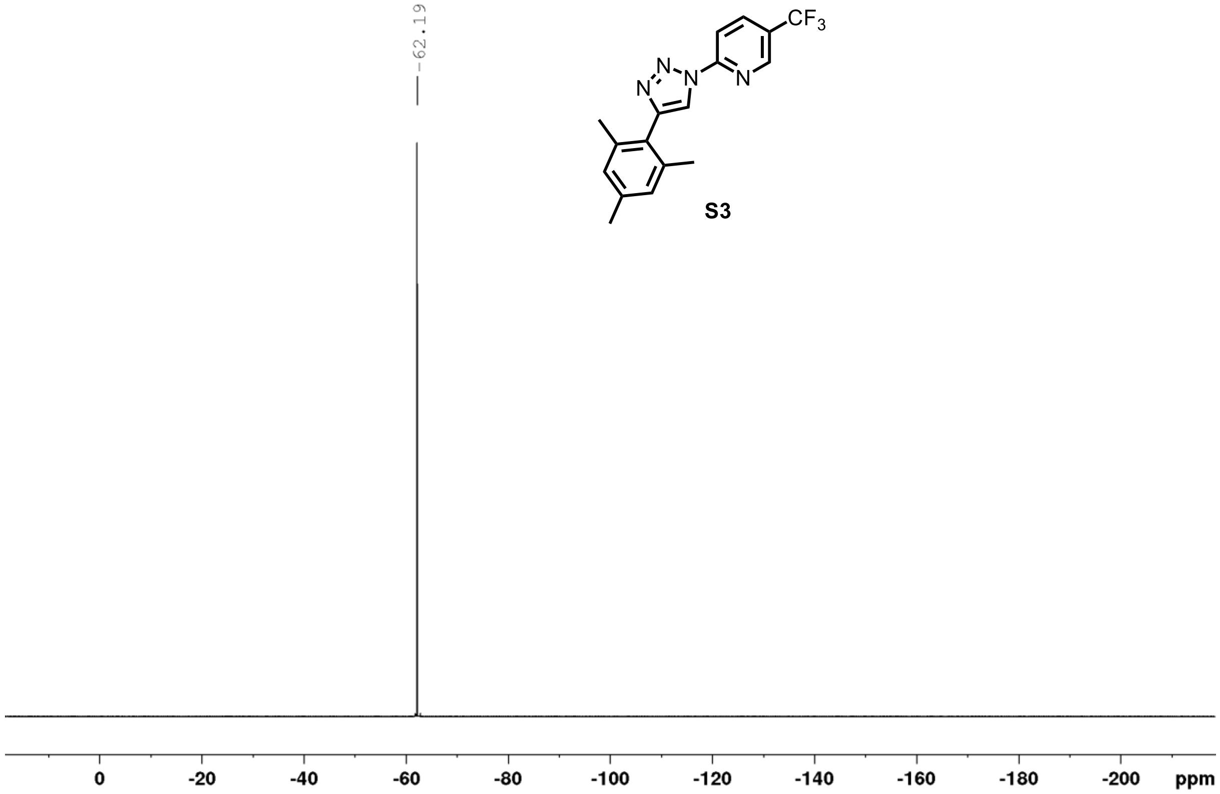

Figure S18. ^{19}F-NMR spectrum of **S3** (282 MHz, $CDCl_3$, 300 K).

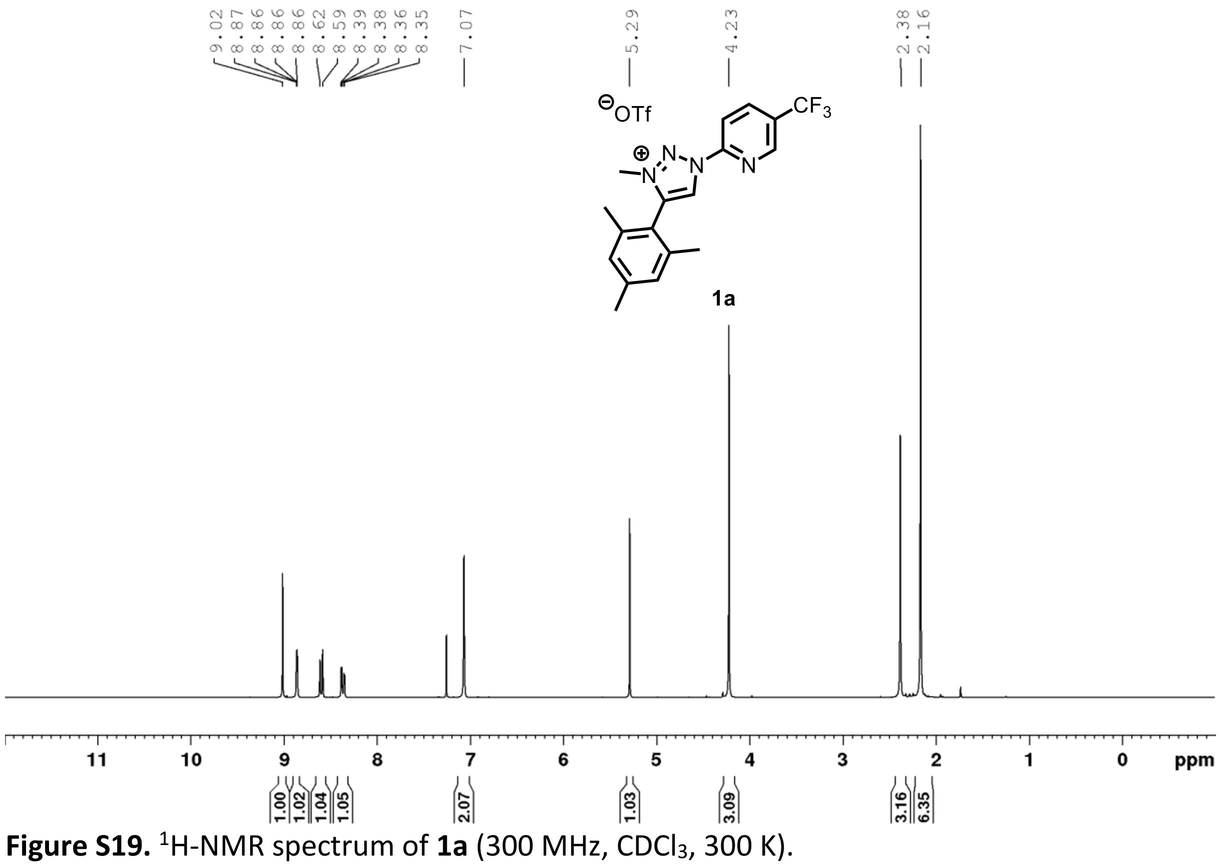

Figure S19. ^{1}H-NMR spectrum of **1a** (300 MHz, $CDCl_3$, 300 K).

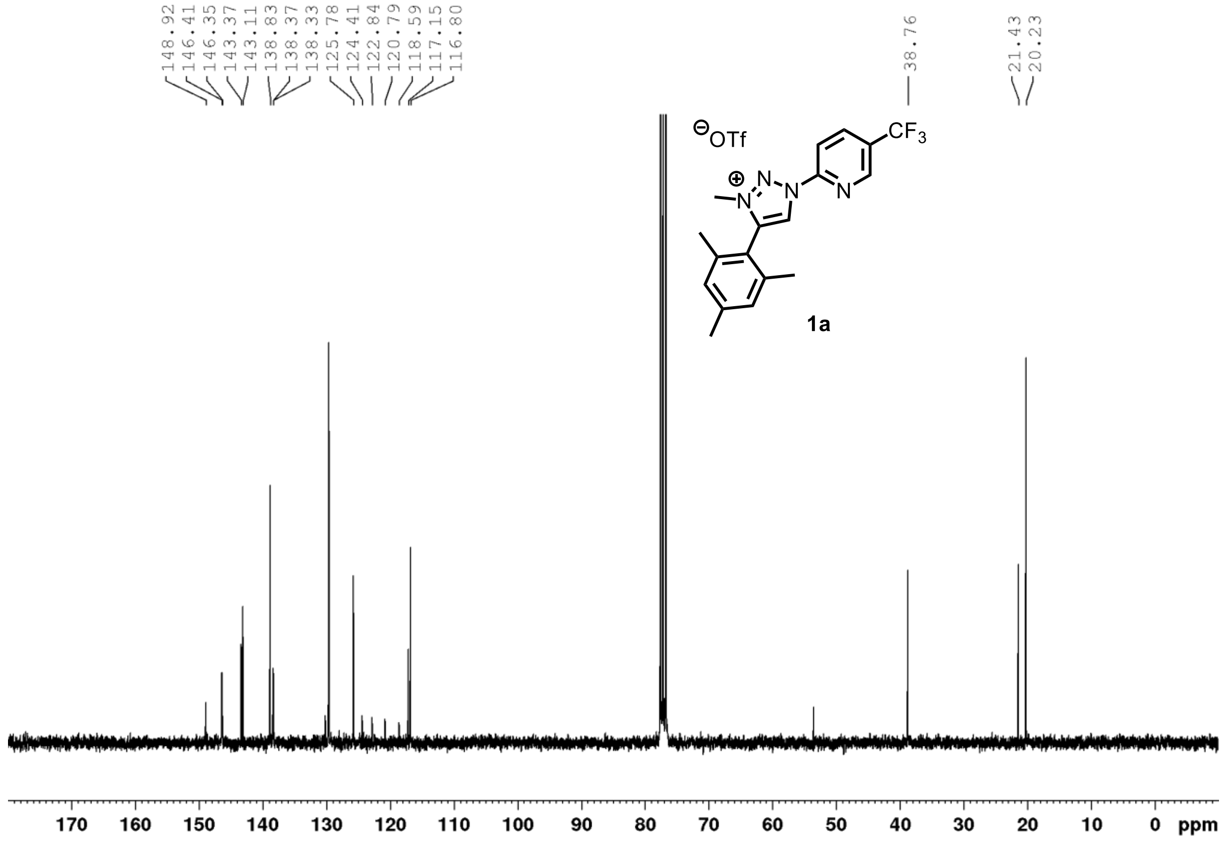

Figure S20. ^{13}C-NMR spectrum of **1a** (75 MHz, $CDCl_3$, 300 K).

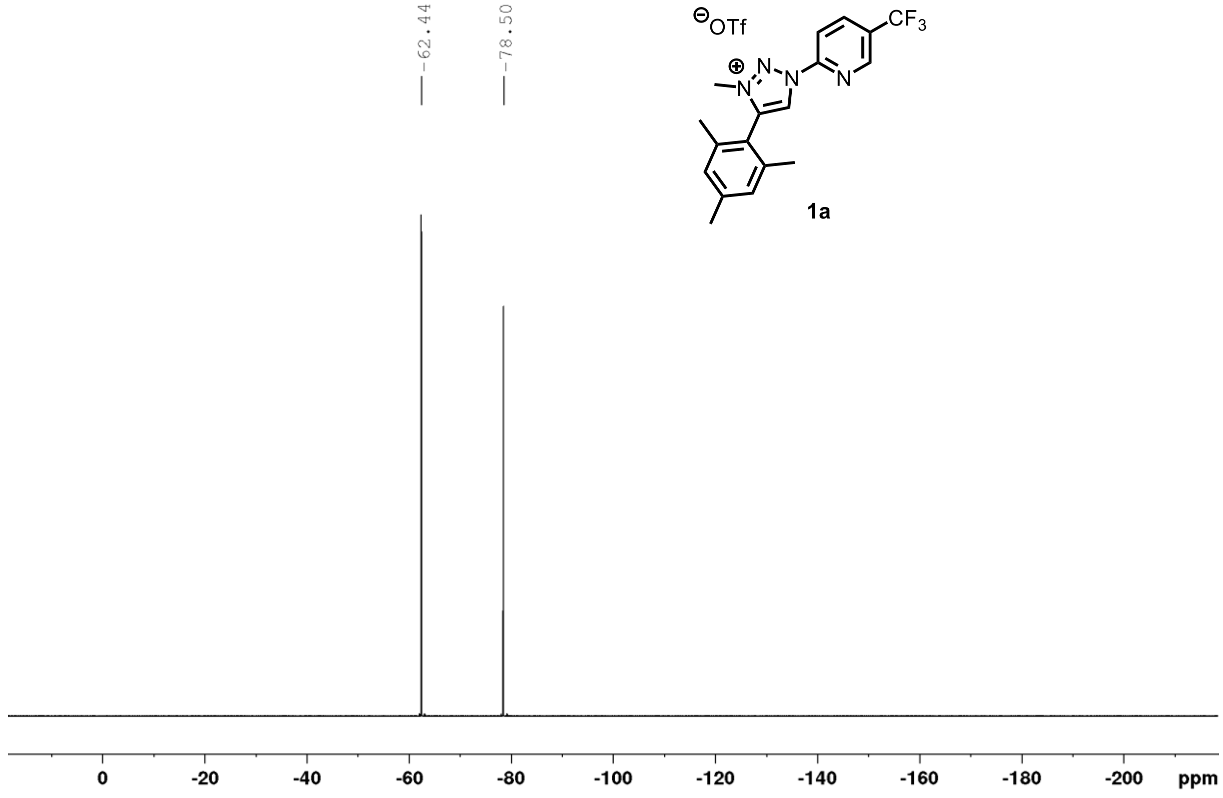

Figure S21. ^{19}F-NMR spectrum of **1a** (282 MHz, $CDCl_3$, 300 K).

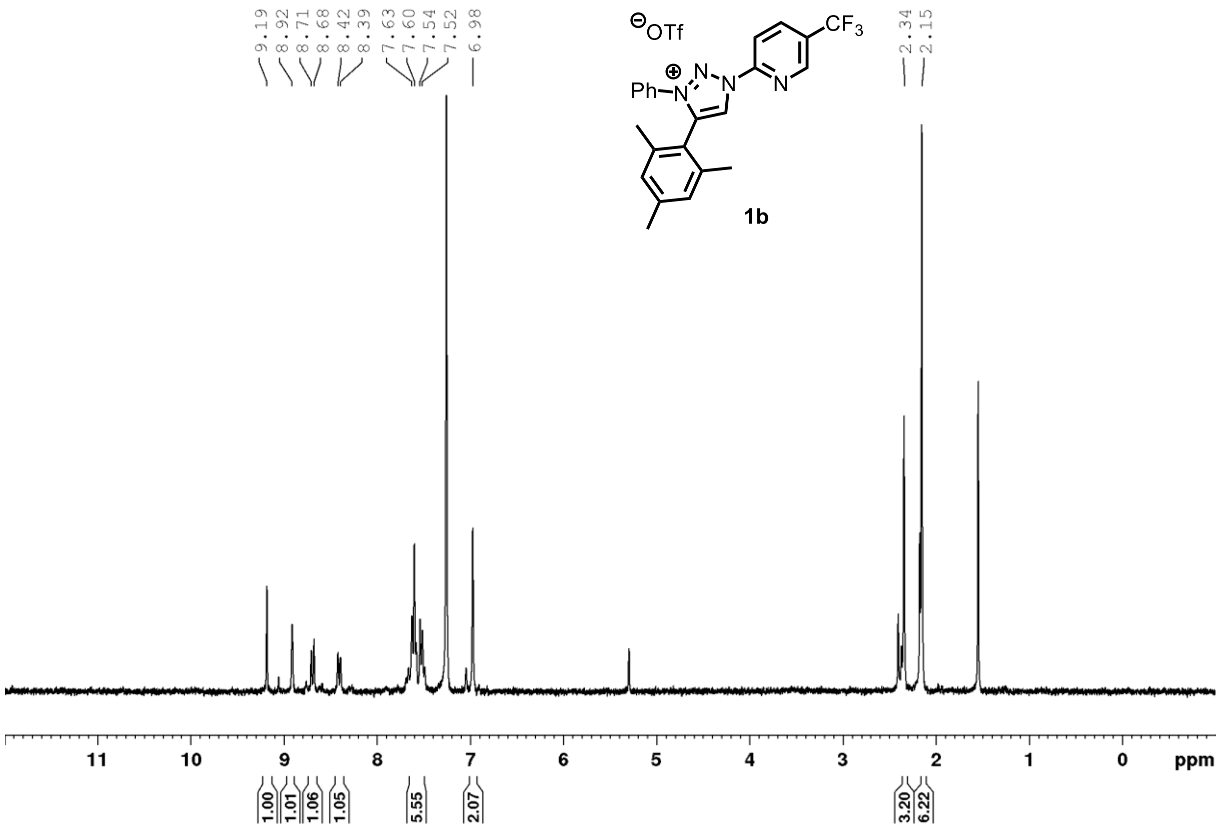

Figure S22. ^{1}H-NMR spectrum of **1b** (300 MHz, $CDCl_3$, 300 K).

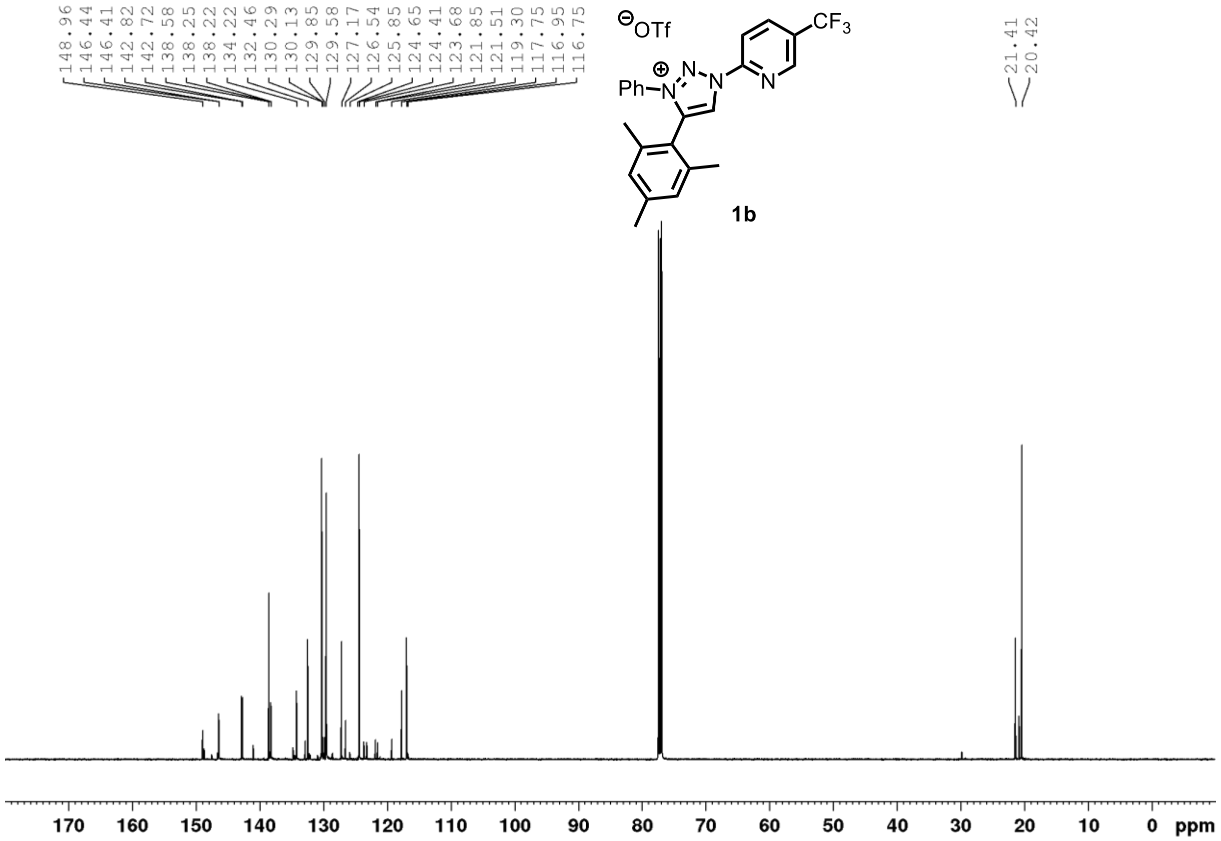

Figure S23. ^{13}C-NMR spectrum of **1b** (125 MHz, $CDCl_3$, 300 K).

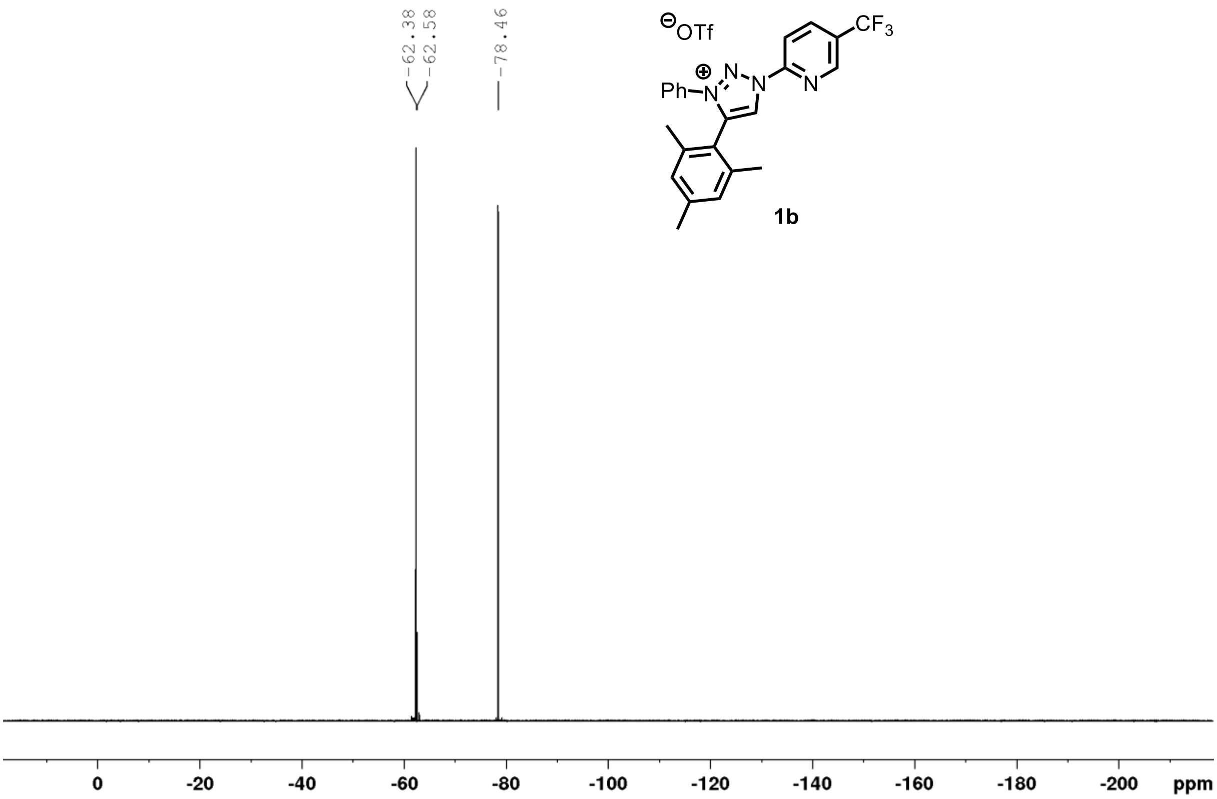

Figure S24. ^{19}F-NMR spectrum of **1b** (282 MHz, $CDCl_3$, 300 K).

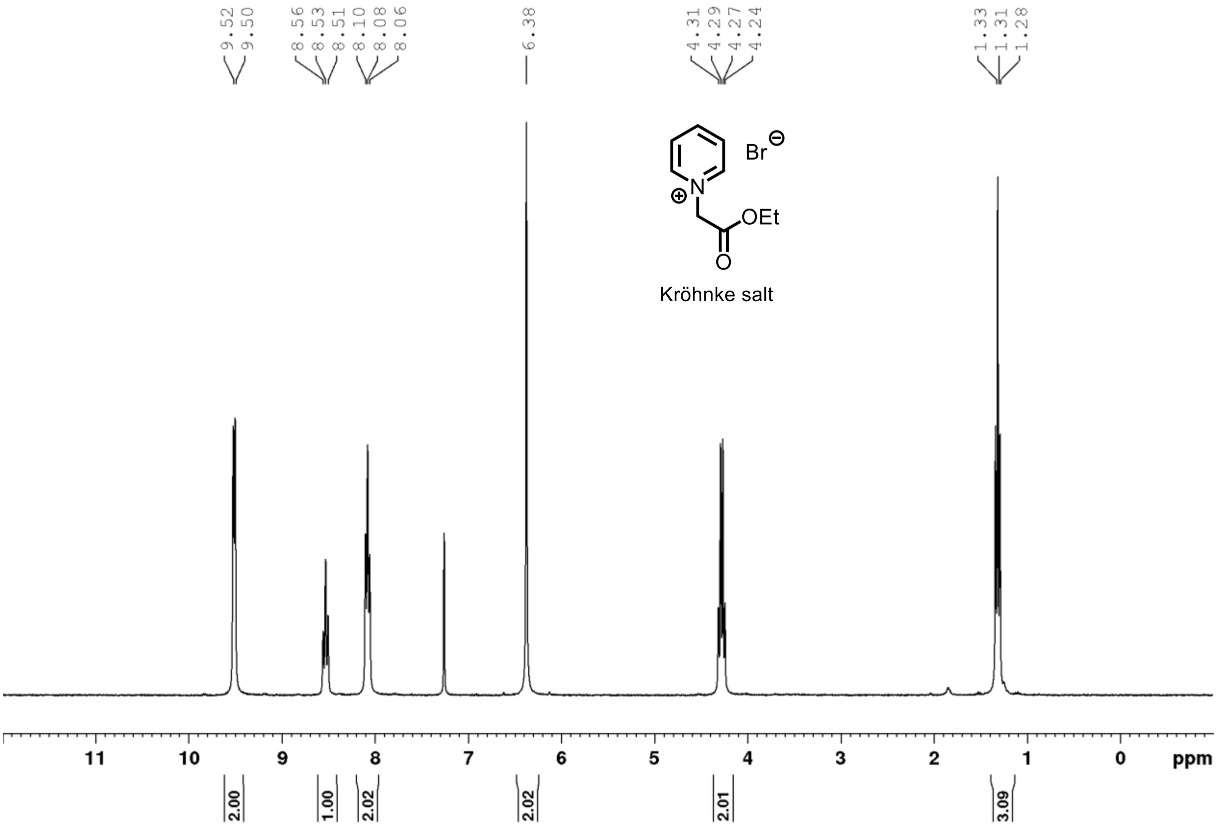

Figure S25. ^{1}H-NMR spectrum of **Kröhnke salt** (300 MHz, $CDCl_3$, 300 K).

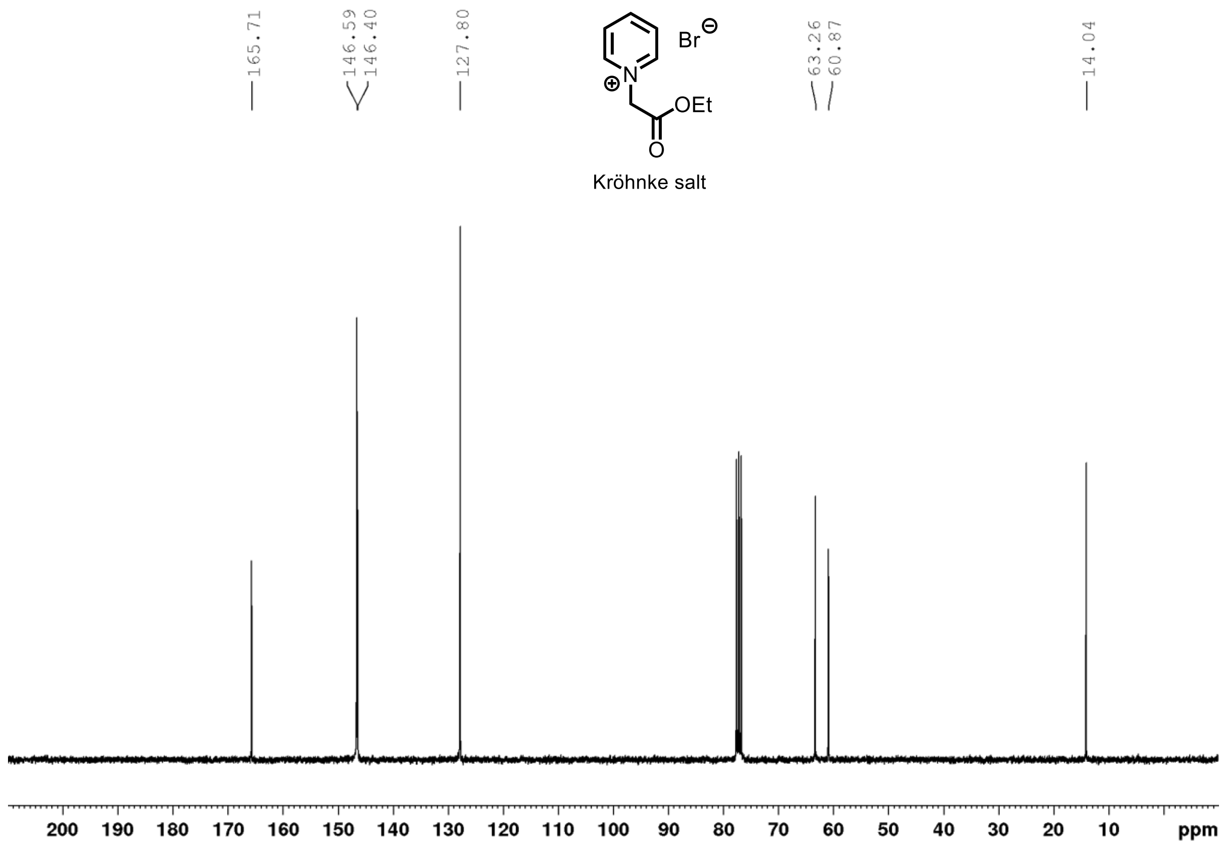

Figure S26. ^{13}C-NMR spectrum of **Kröhnke salt** (75 MHz, $CDCl_3$, 300 K).

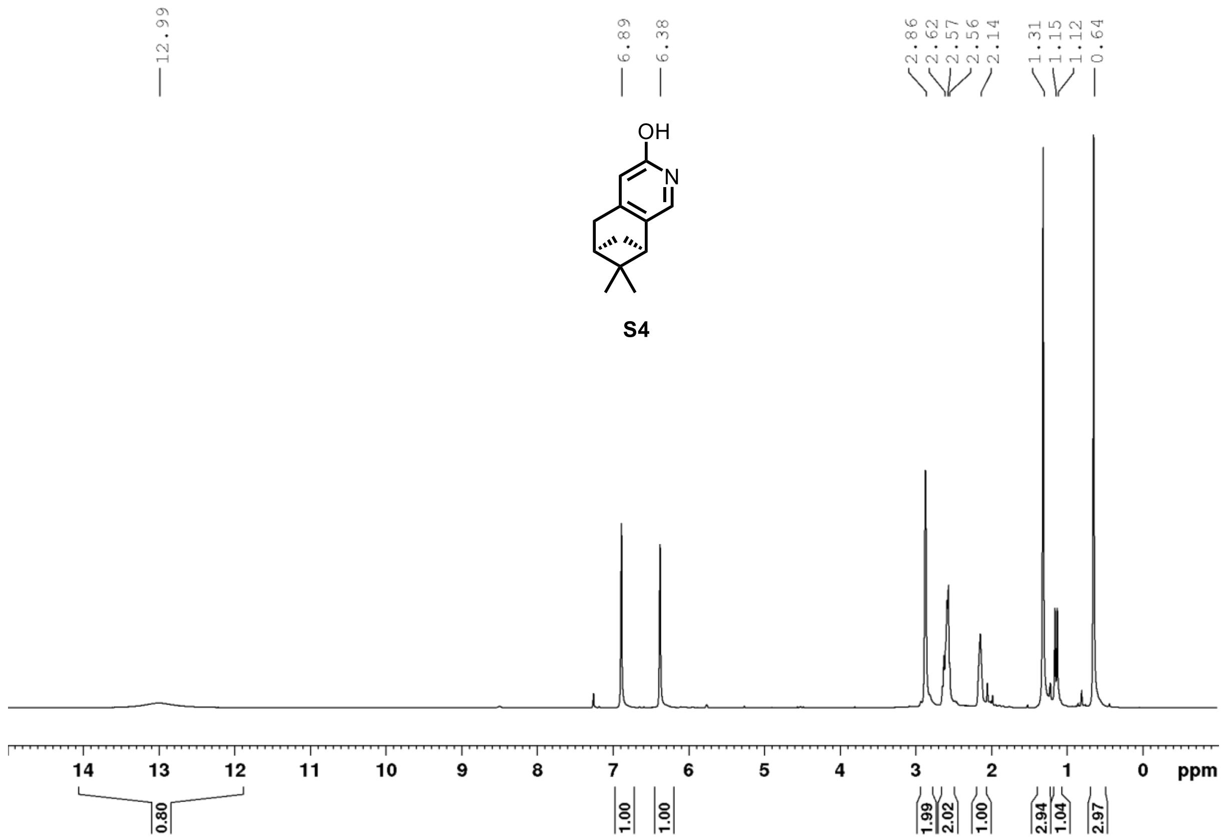

Figure S27. ^{1}H-NMR spectrum of **S4** (300 MHz, $CDCl_3$, 300 K).

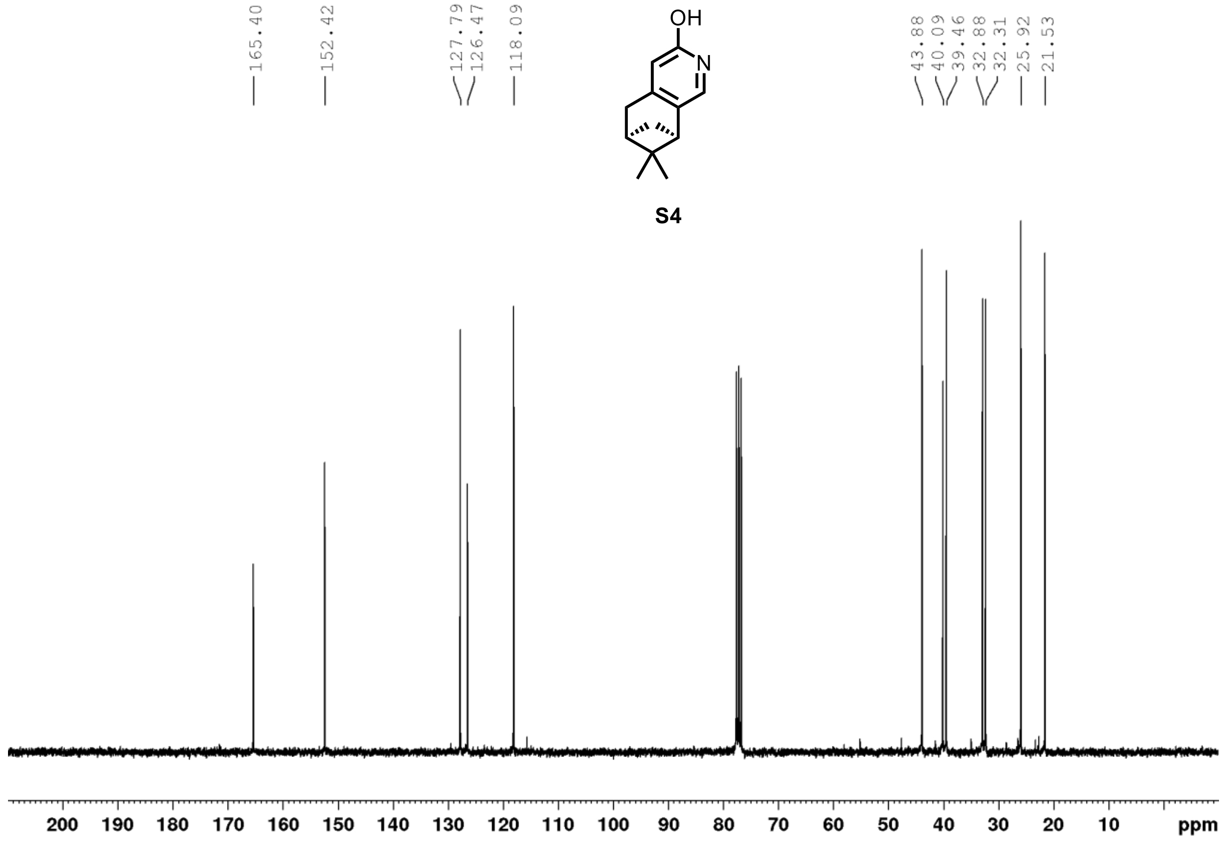

Figure S28. ^{13}C-NMR spectrum of **S4** (75 MHz, $CDCl_3$, 300 K).

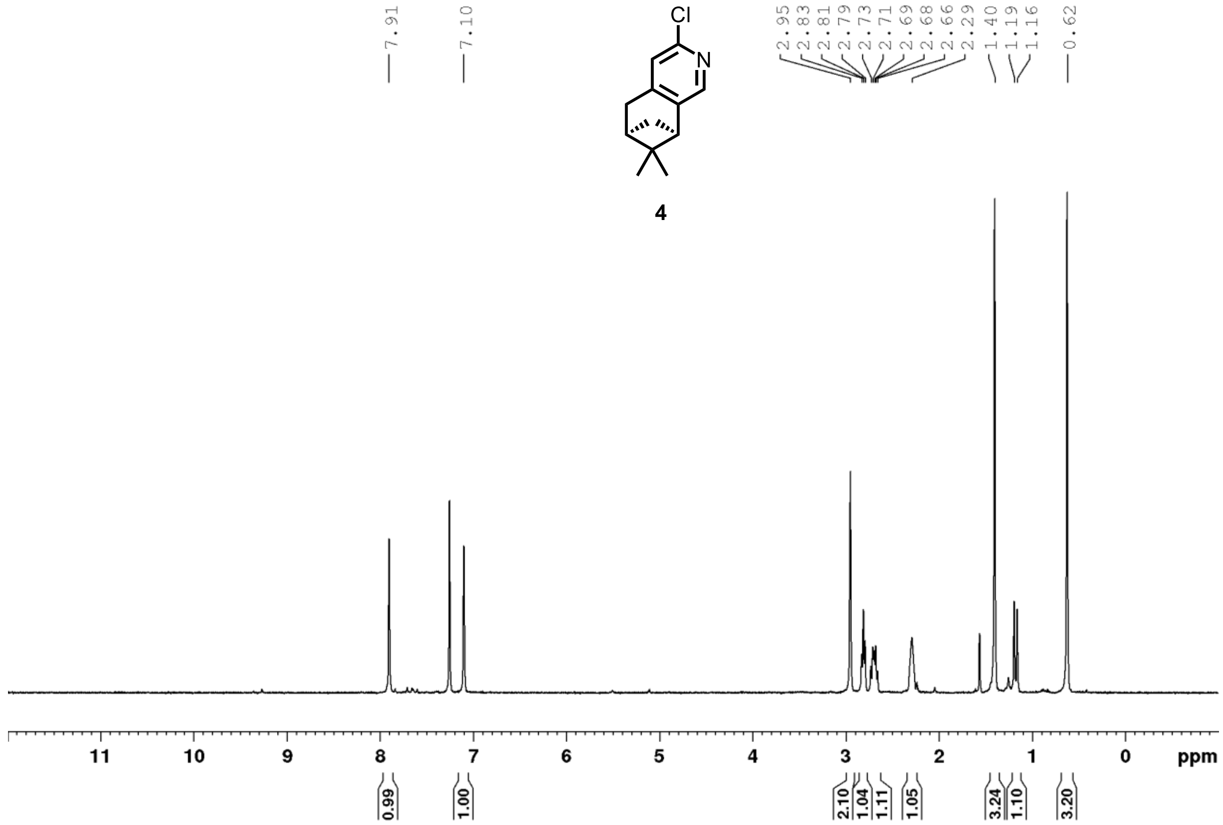

Figure S29. ^{1}H-NMR spectrum of **4** (300 MHz, $CDCl_3$, 300 K).

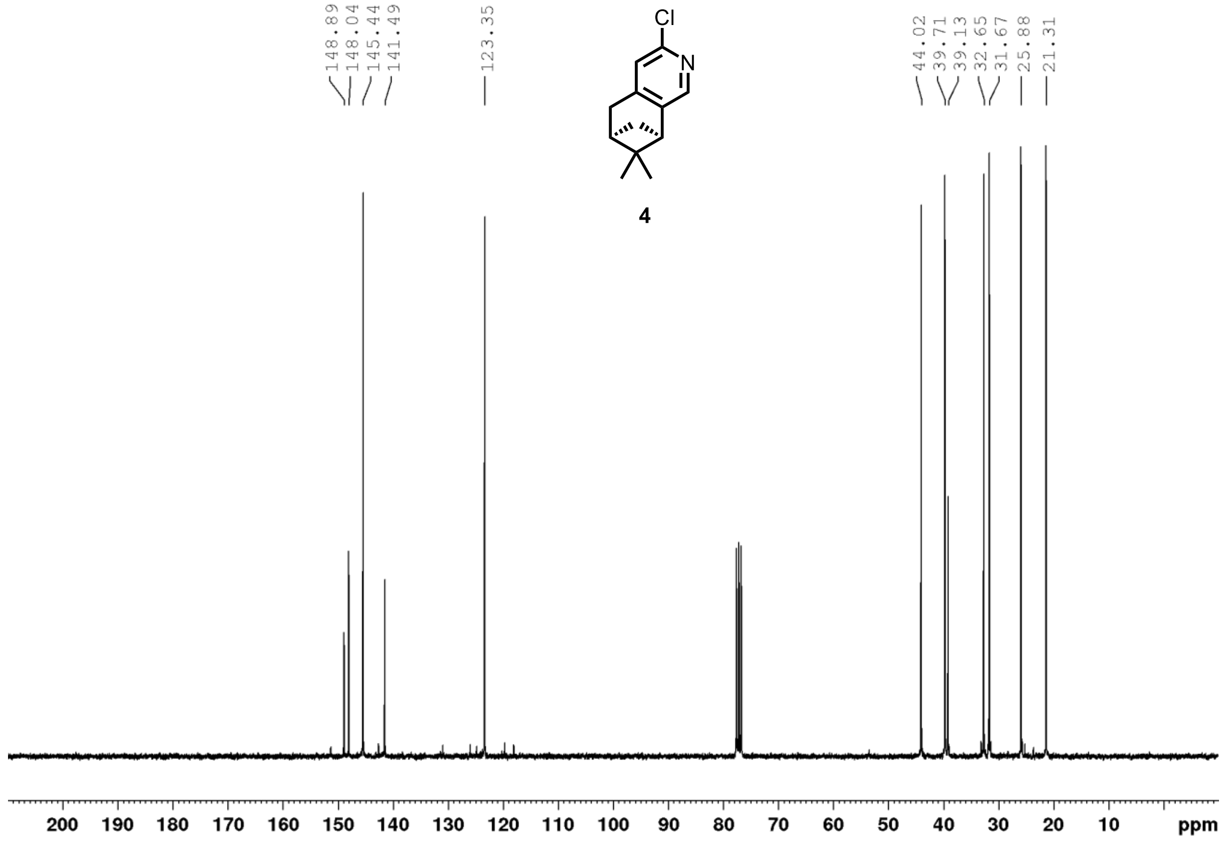

Figure S30. ^{13}C-NMR spectrum of **4** (75 MHz, $CDCl_3$, 300 K).

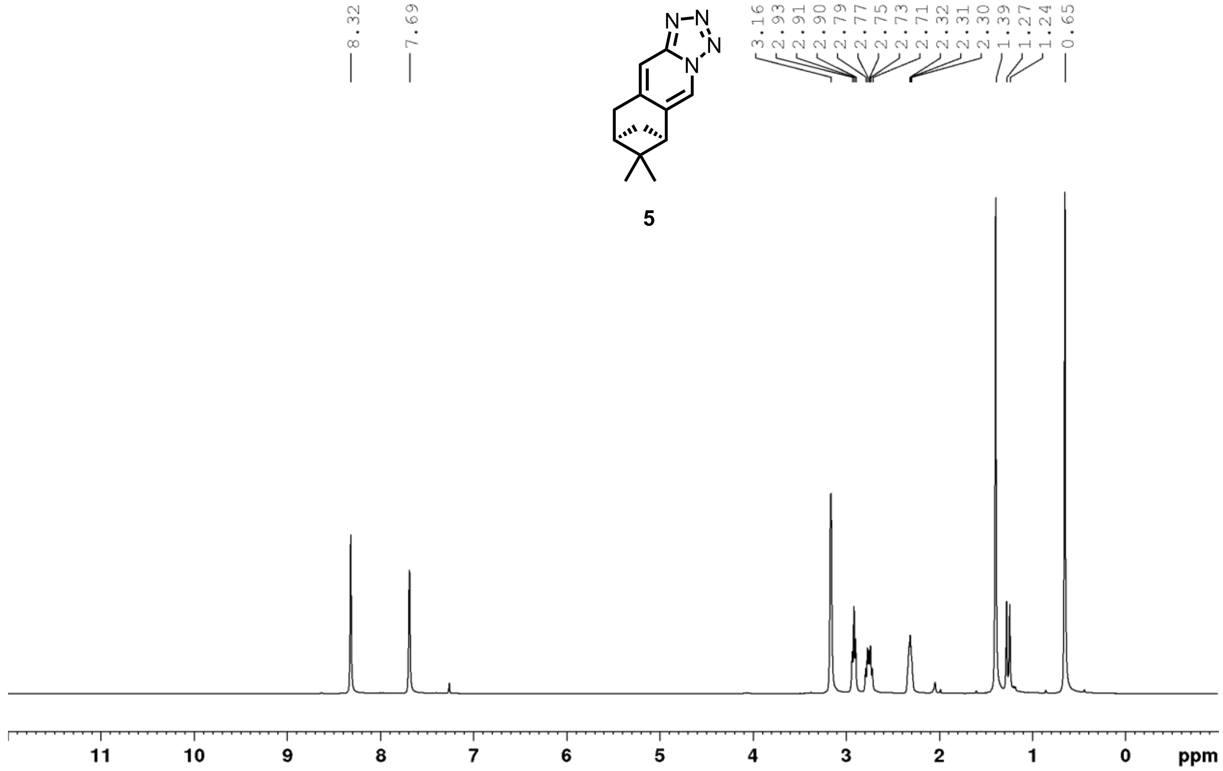

Figure S31. ^{1}H-NMR spectrum of **5** (300 MHz, $CDCl_3$, 300 K).

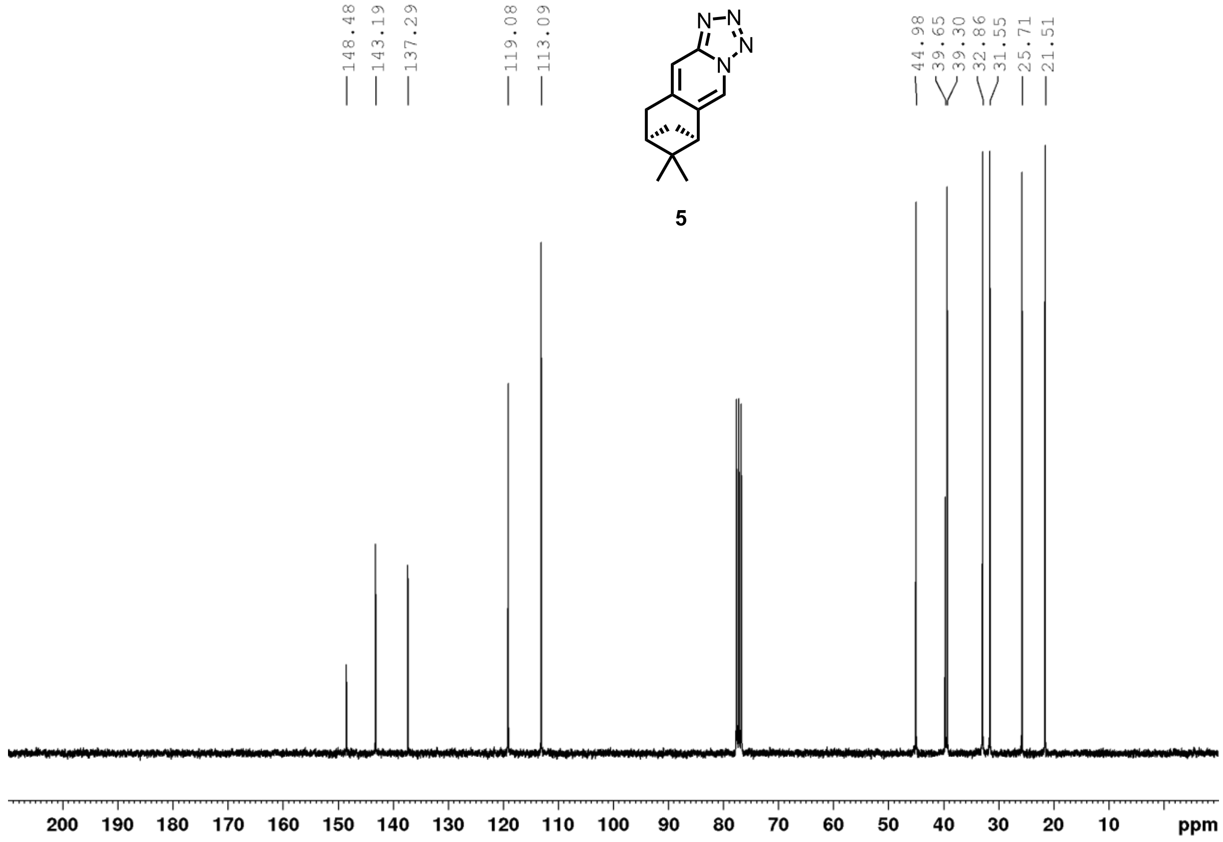

Figure S32. ^{13}C-NMR spectrum of **5** (75 MHz, $CDCl_3$, 300 K).

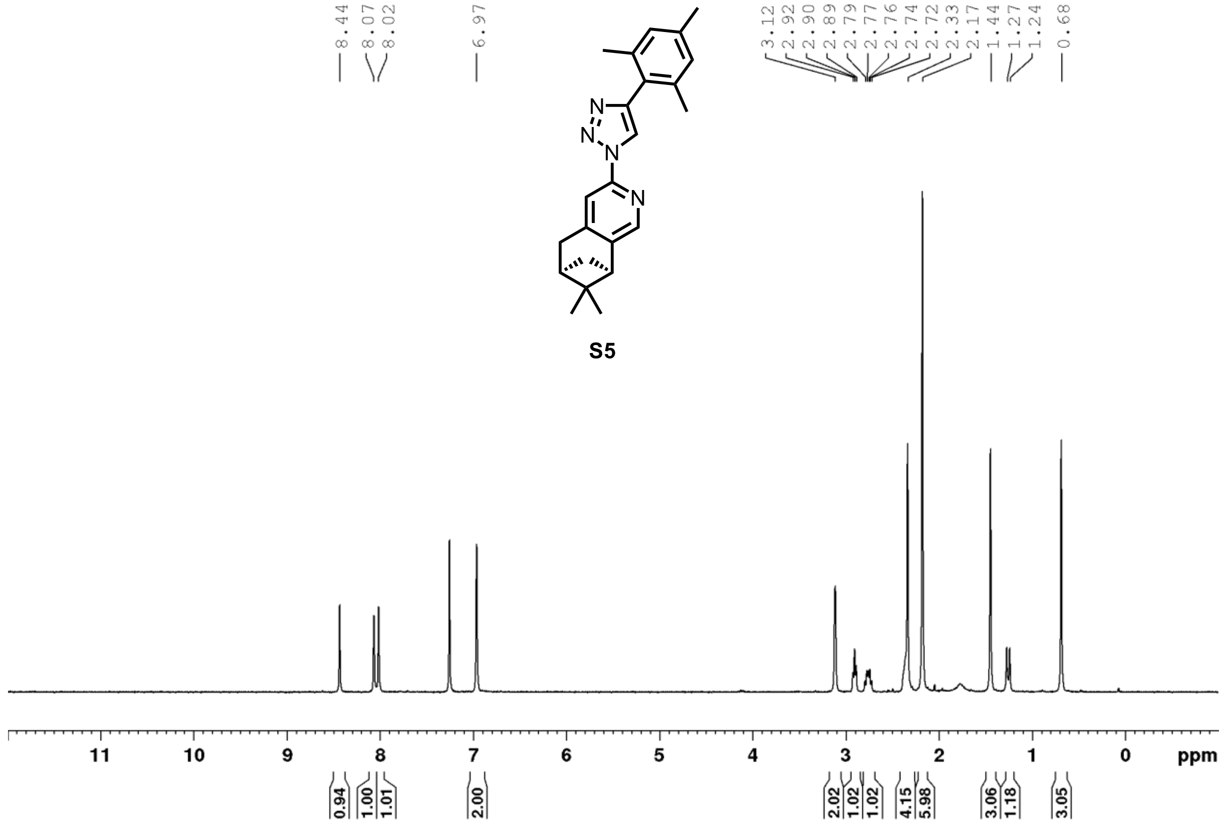

Figure S33. ^{1}H-NMR spectrum of **S5** (300 MHz, $CDCl_3$, 300 K).

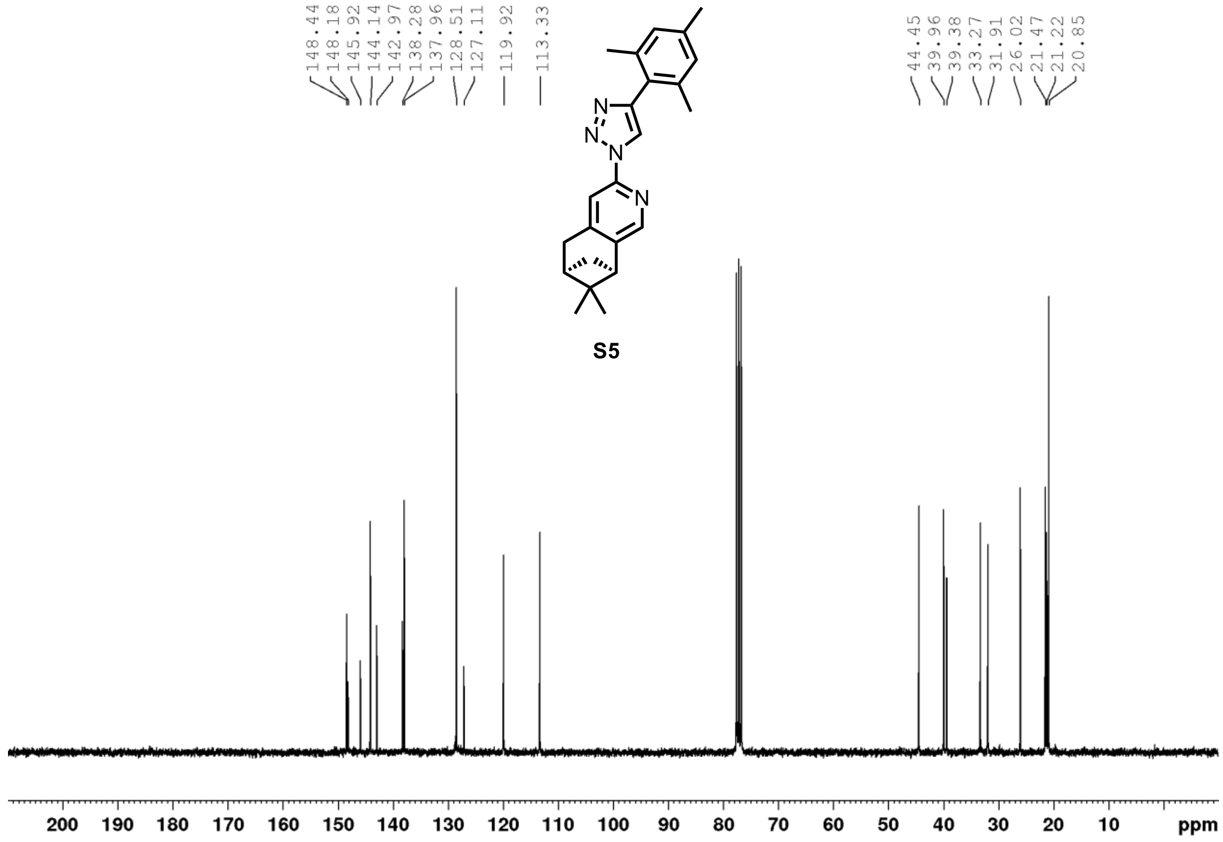

Figure S34. ^{13}C-NMR spectrum of **S5** (75 MHz, $CDCl_3$, 300 K).

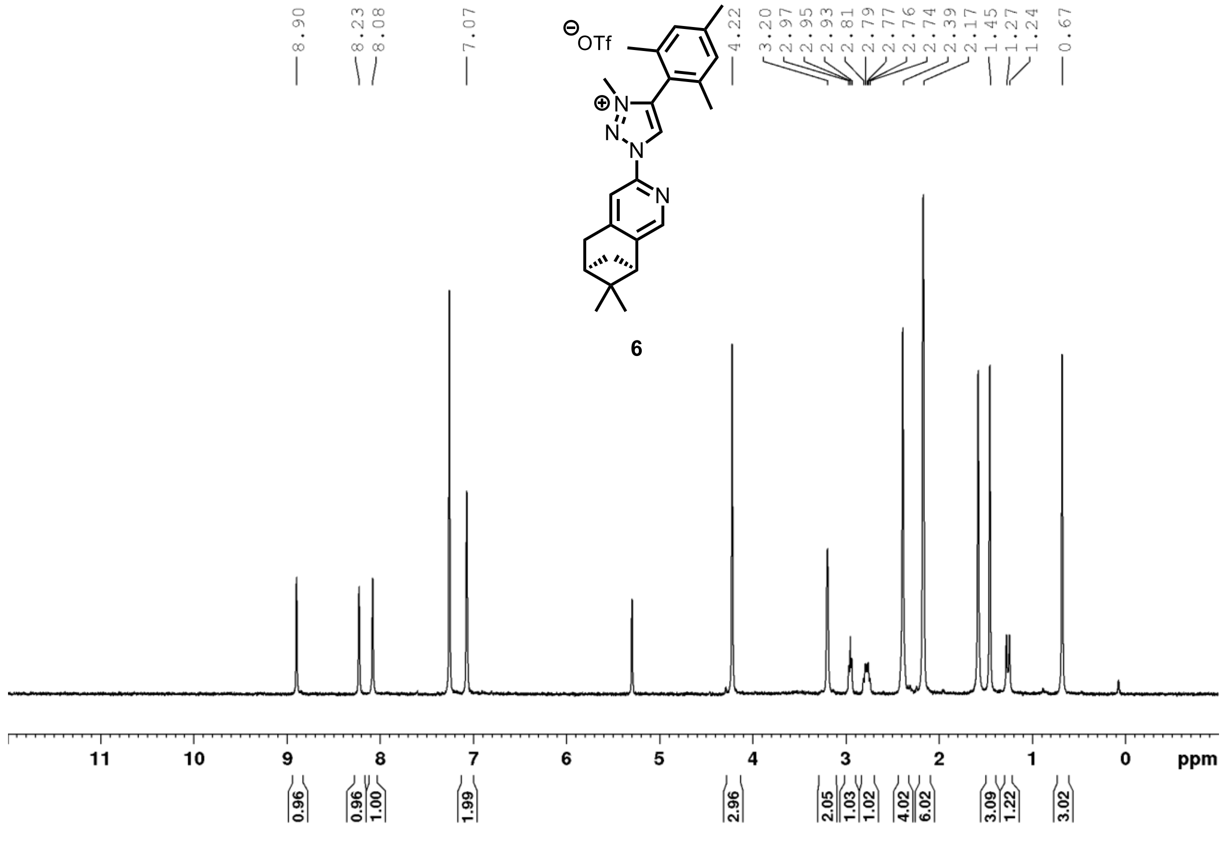

Figure S35. ^{1}H-NMR spectrum of **6** (300 MHz, $CDCl_3$, 300 K).

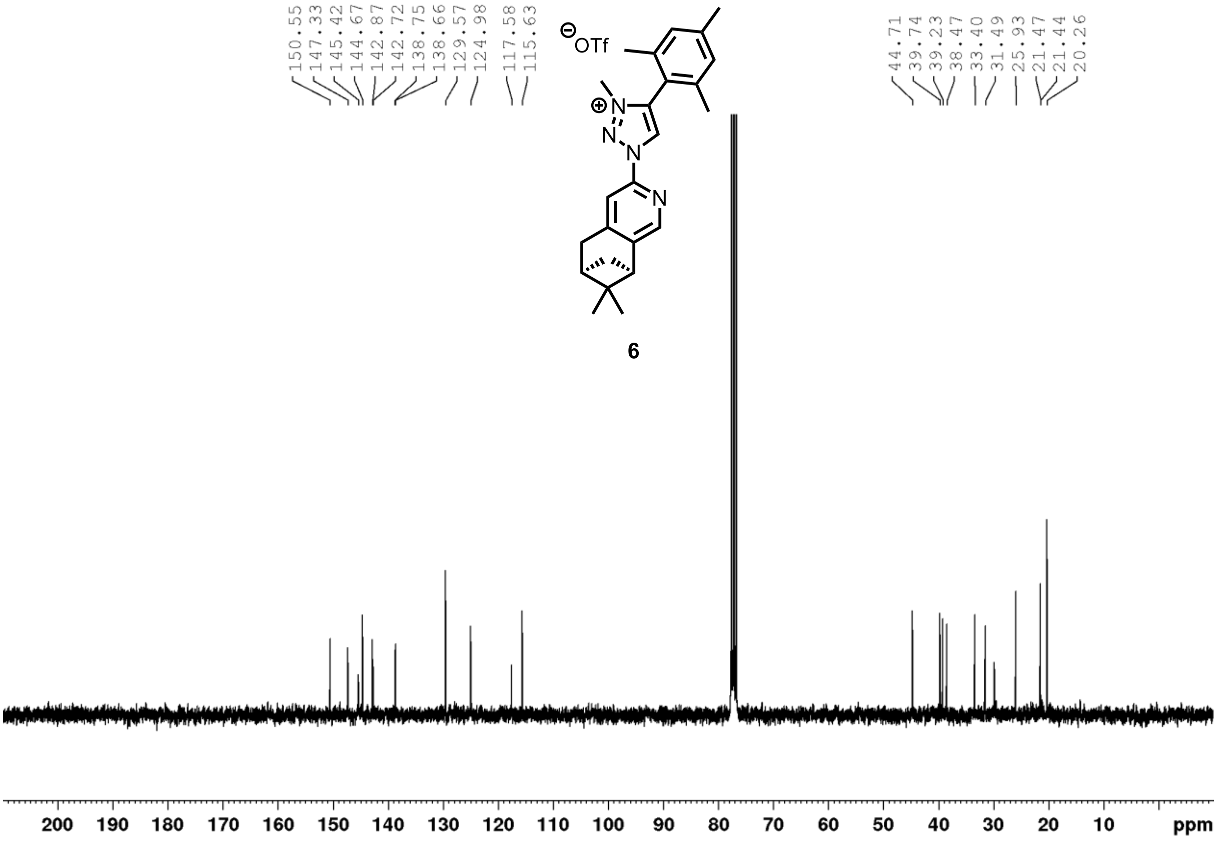

Figure S36. ^{13}C-NMR spectrum of **6** (75 MHz, $CDCl_3$, 300 K).

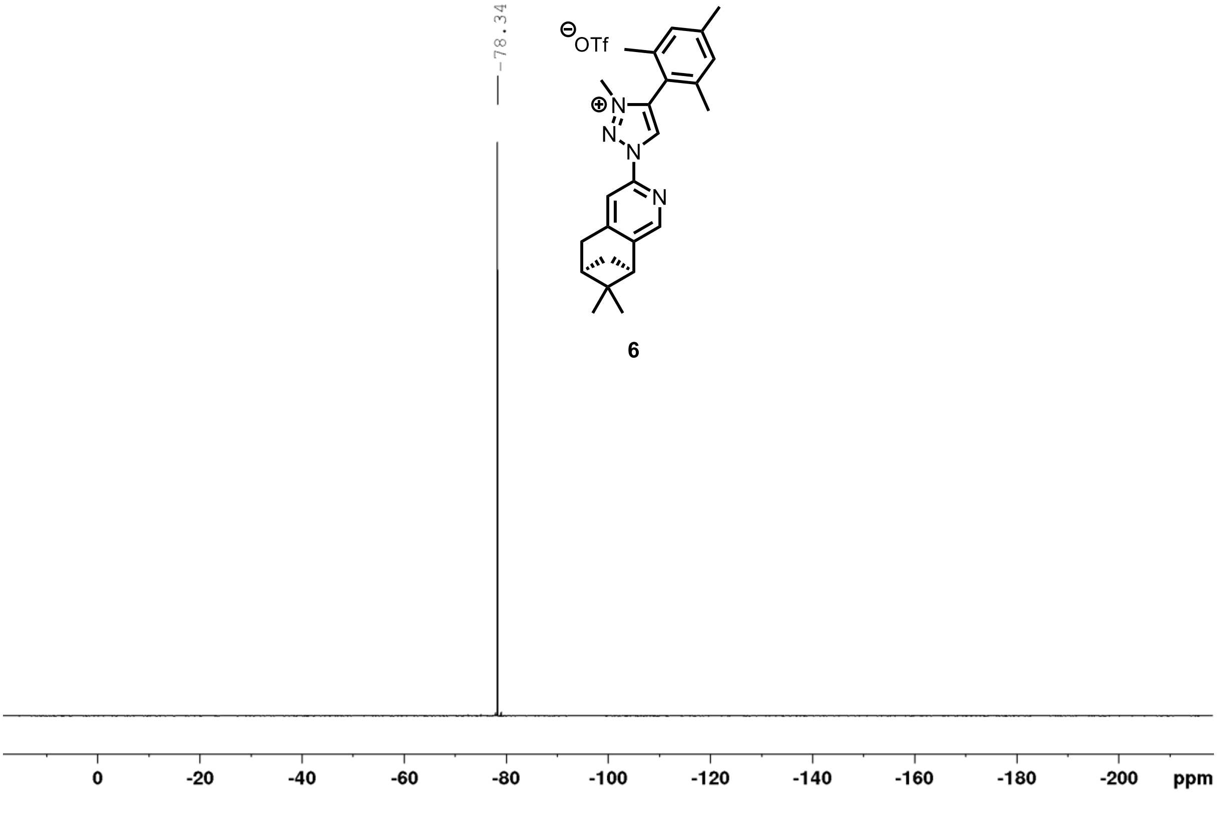

Figure S37. ^{19}F-NMR spectrum of **6** (282 MHz, $CDCl_3$, 300 K).

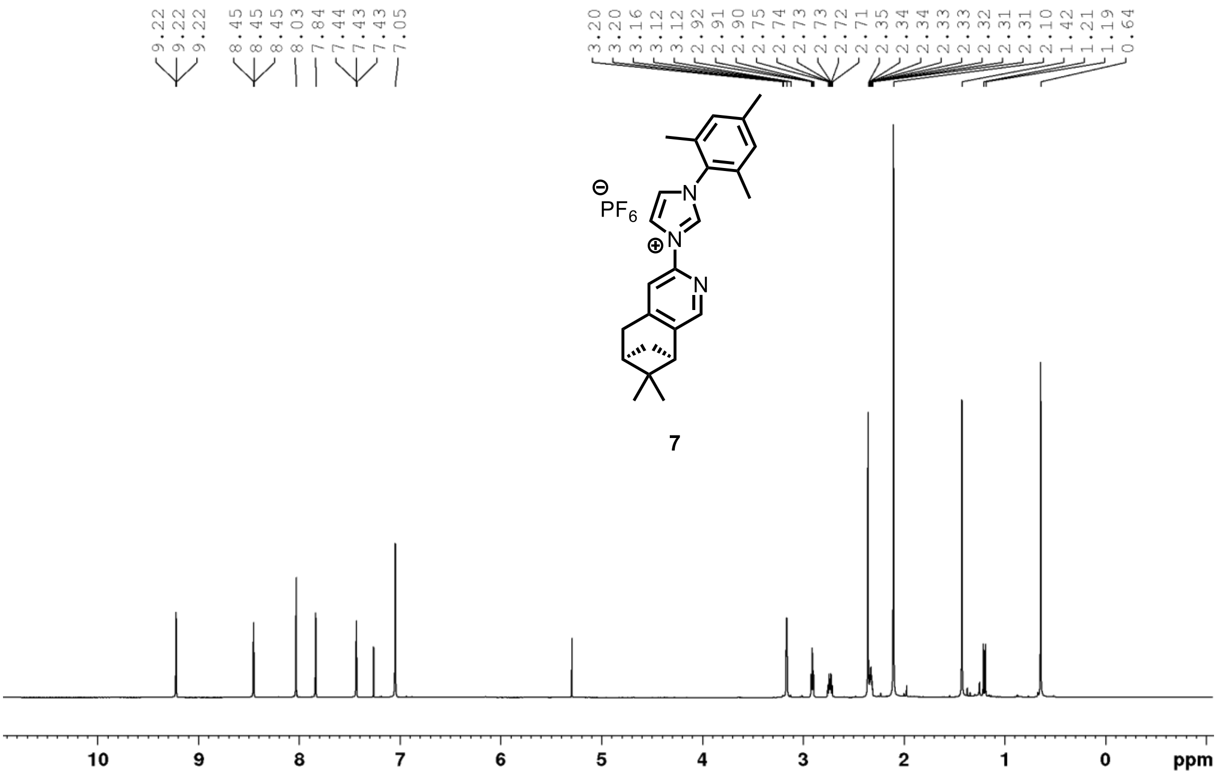

Figure S38. ^{1}H-NMR spectrum of **7** (500 MHz, $CDCl_3$, 300 K).

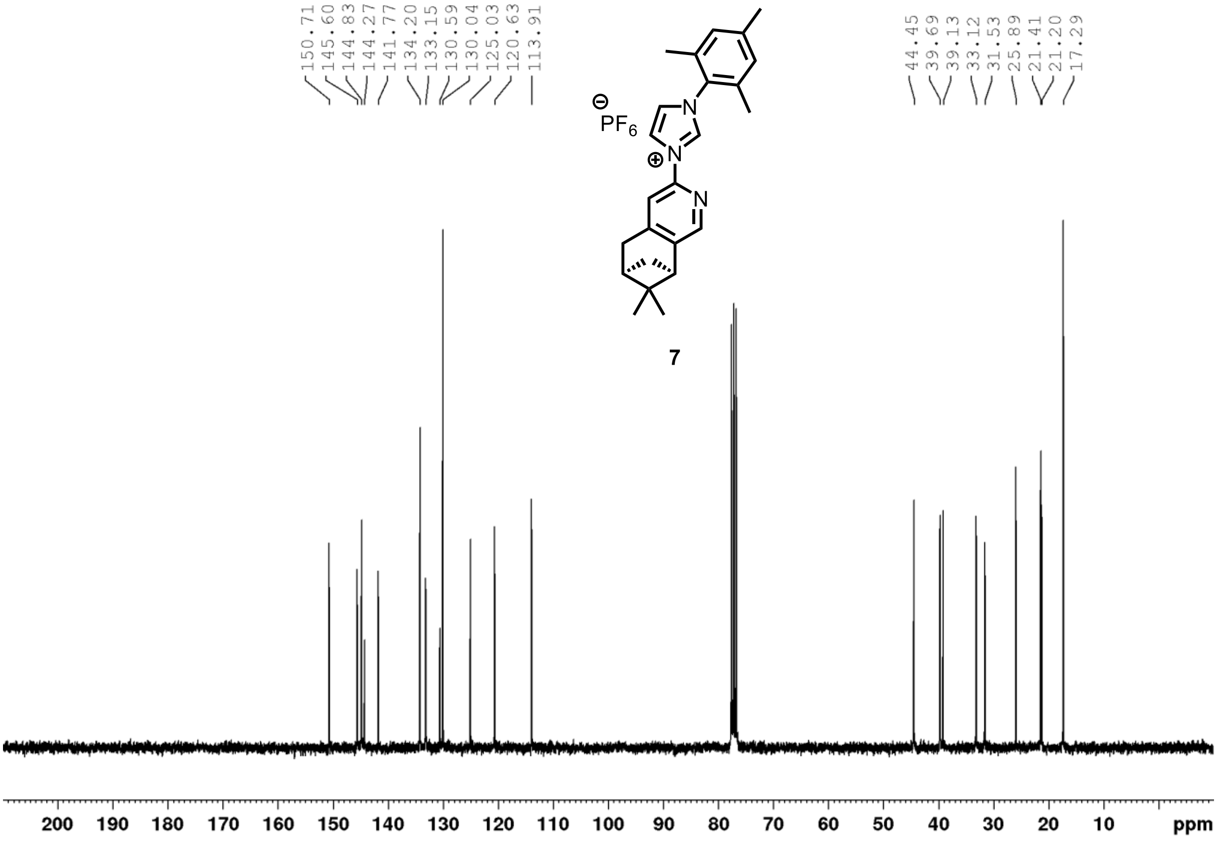

Figure S39. ^{13}C-NMR spectrum of **7** (75 MHz, $CDCl_3$, 300 K).

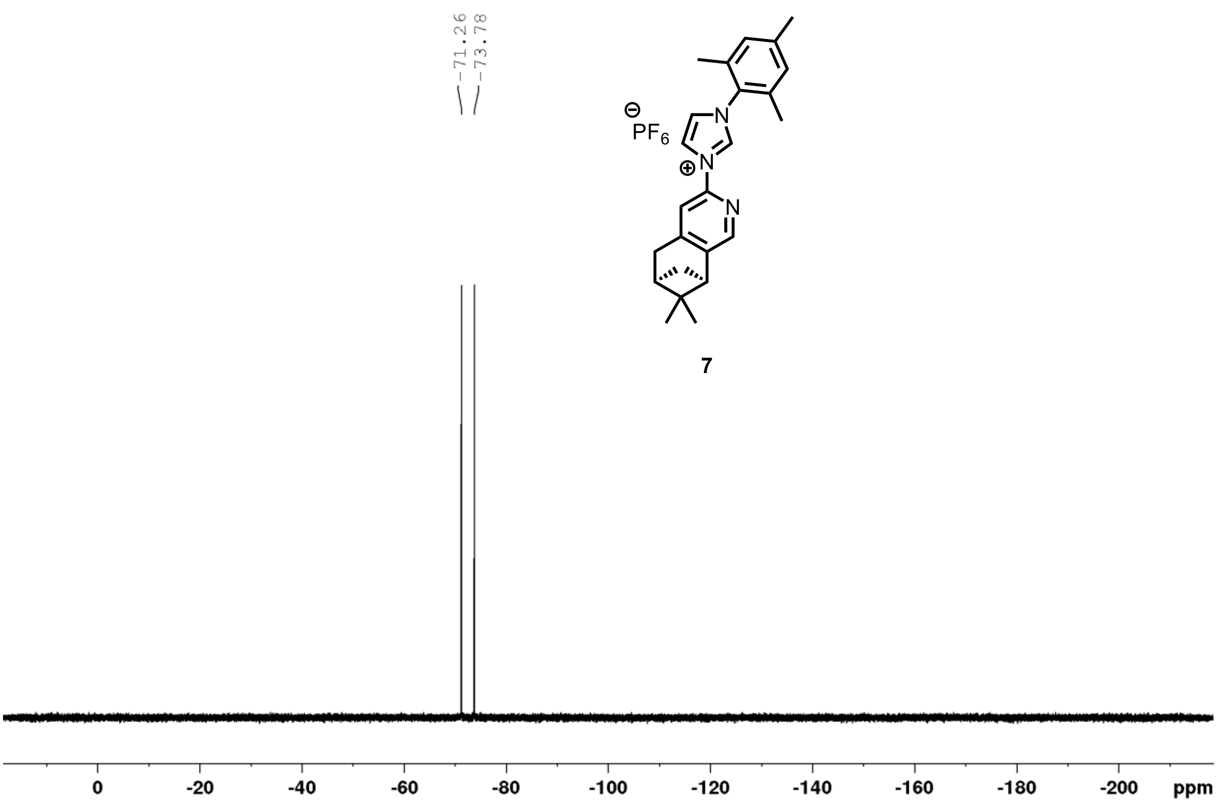

Figure S40. ^{19}F-NMR spectrum of **7** (282 MHz, $CDCl_3$, 300 K).

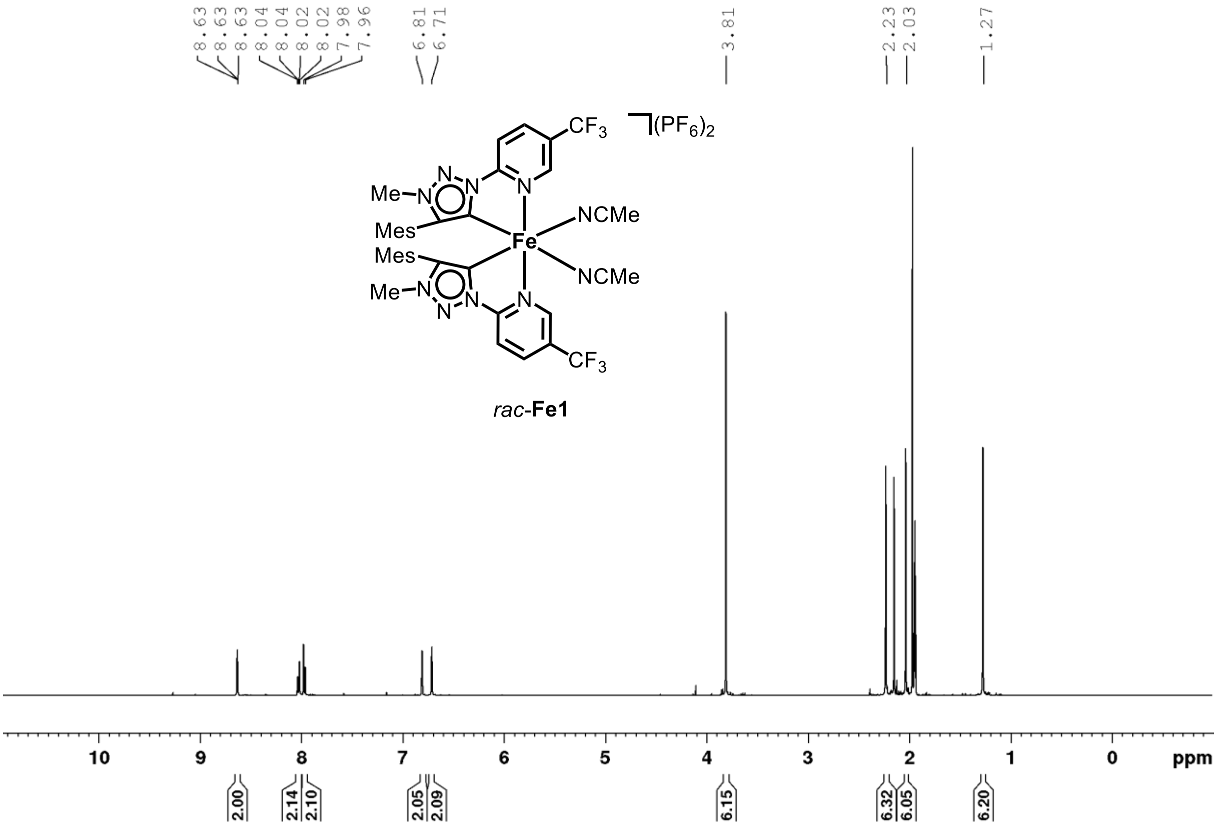

Figure S41. ^{1}H-NMR spectrum of ***rac*-Fe1** (500 MHz, CD_3CN, 300 K).

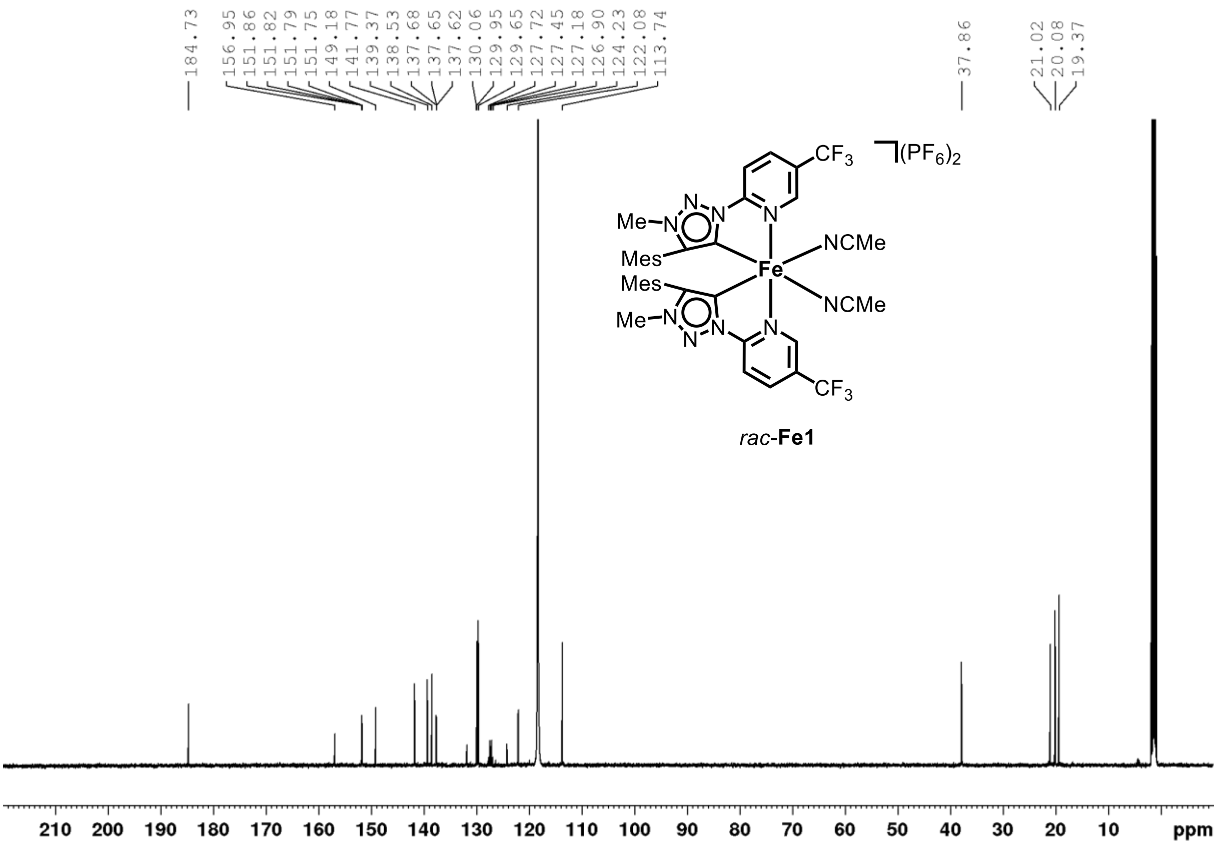

Figure S42. ^{13}C-NMR spectrum of ***rac*-Fe1** (125 MHz, CD_3CN, 300 K).

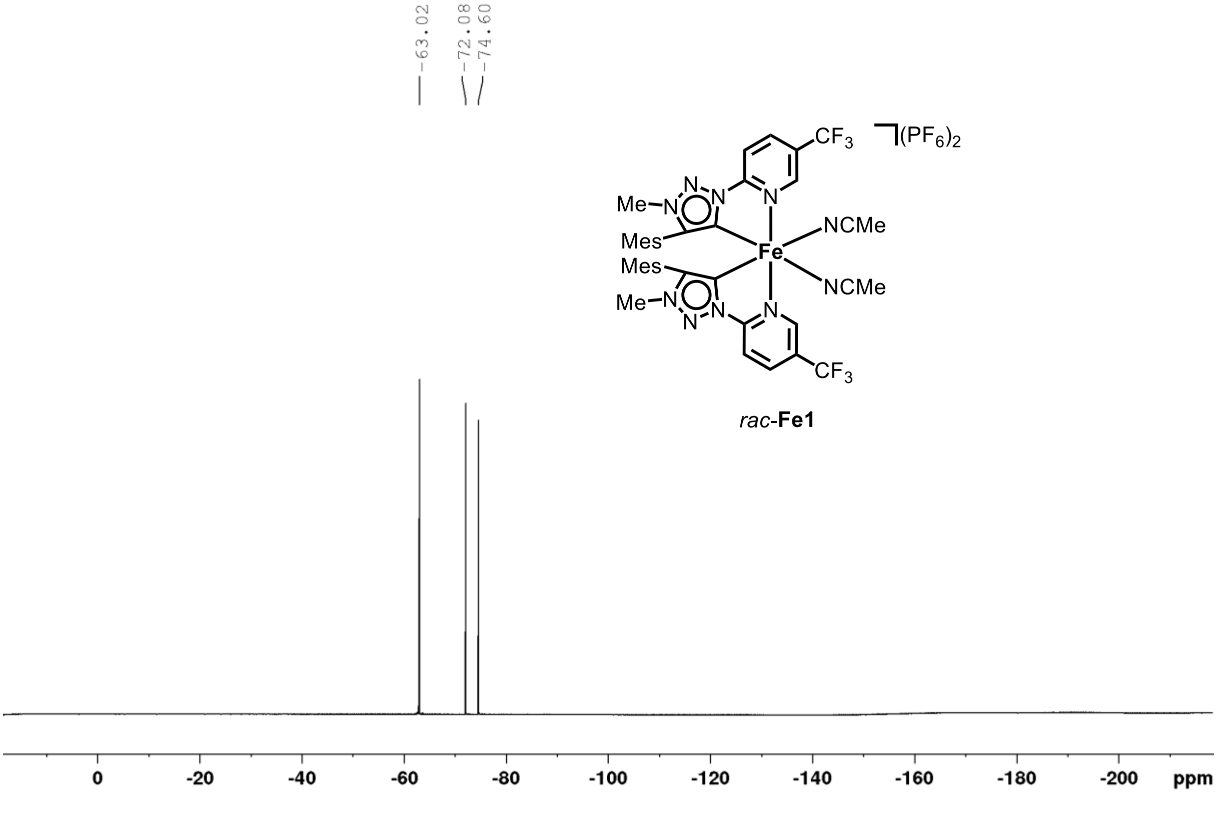

Figure S43. ^{19}F-NMR spectrum of ***rac*-Fe1** (282 MHz, CD_3CN, 300 K).

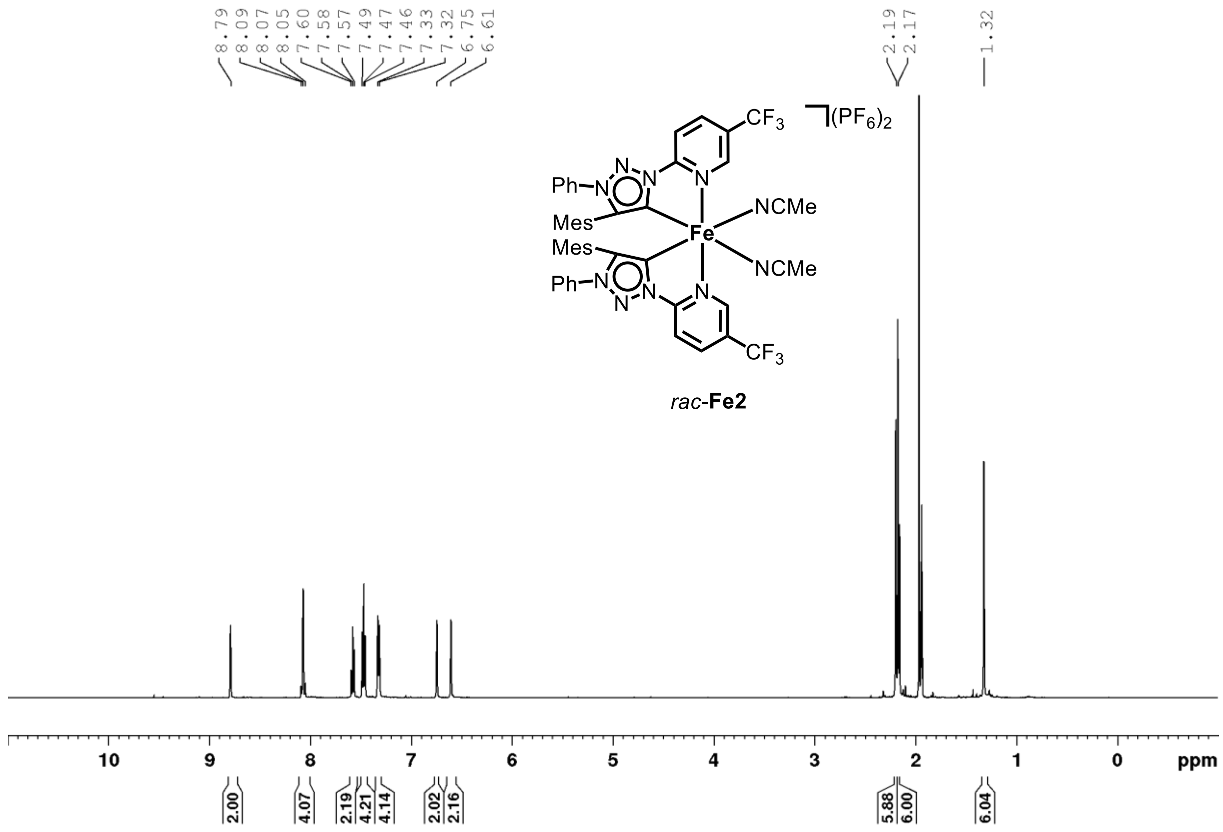

Figure S44. ^{1}H-NMR spectrum of ***rac*-Fe2** (500 MHz, CD_3CN, 300 K).

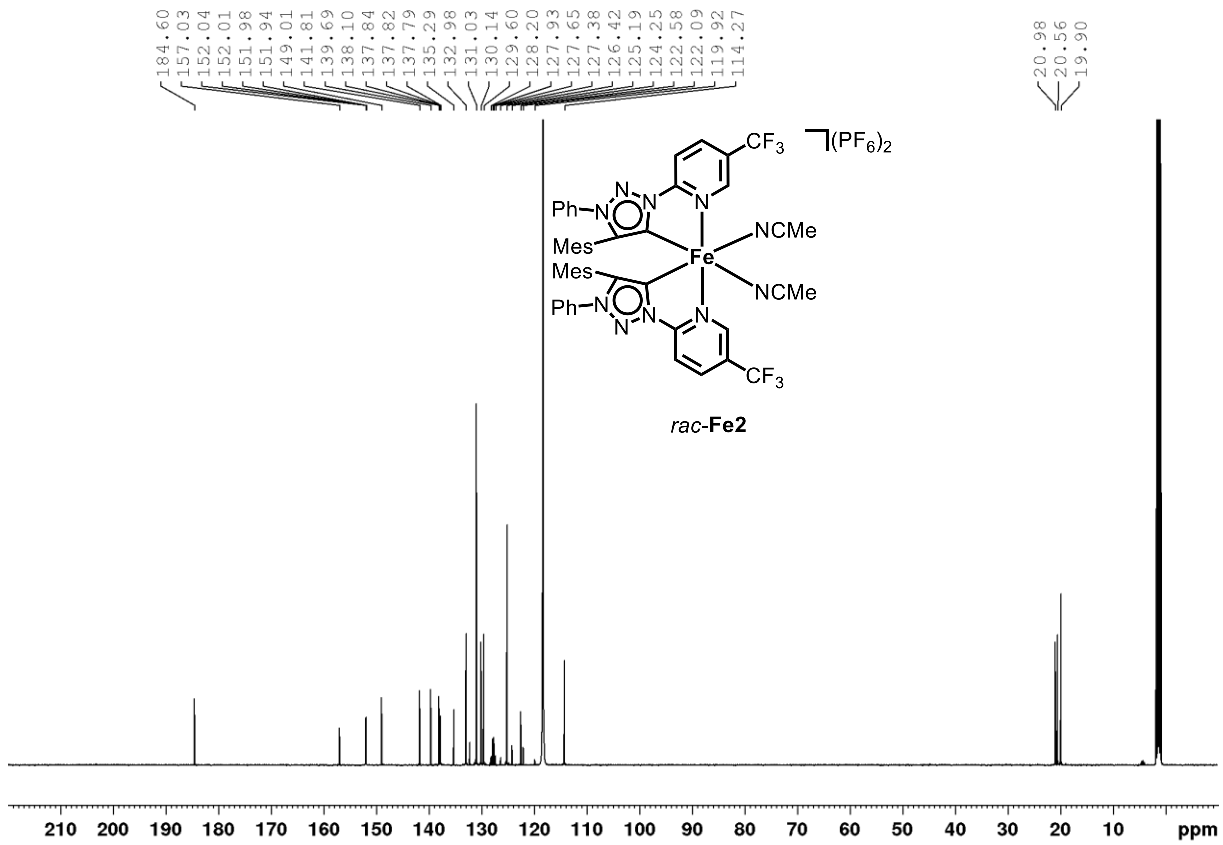

Figure S45. ^{13}C-NMR spectrum of ***rac*-Fe2** (125 MHz, CD_3CN, 300 K).

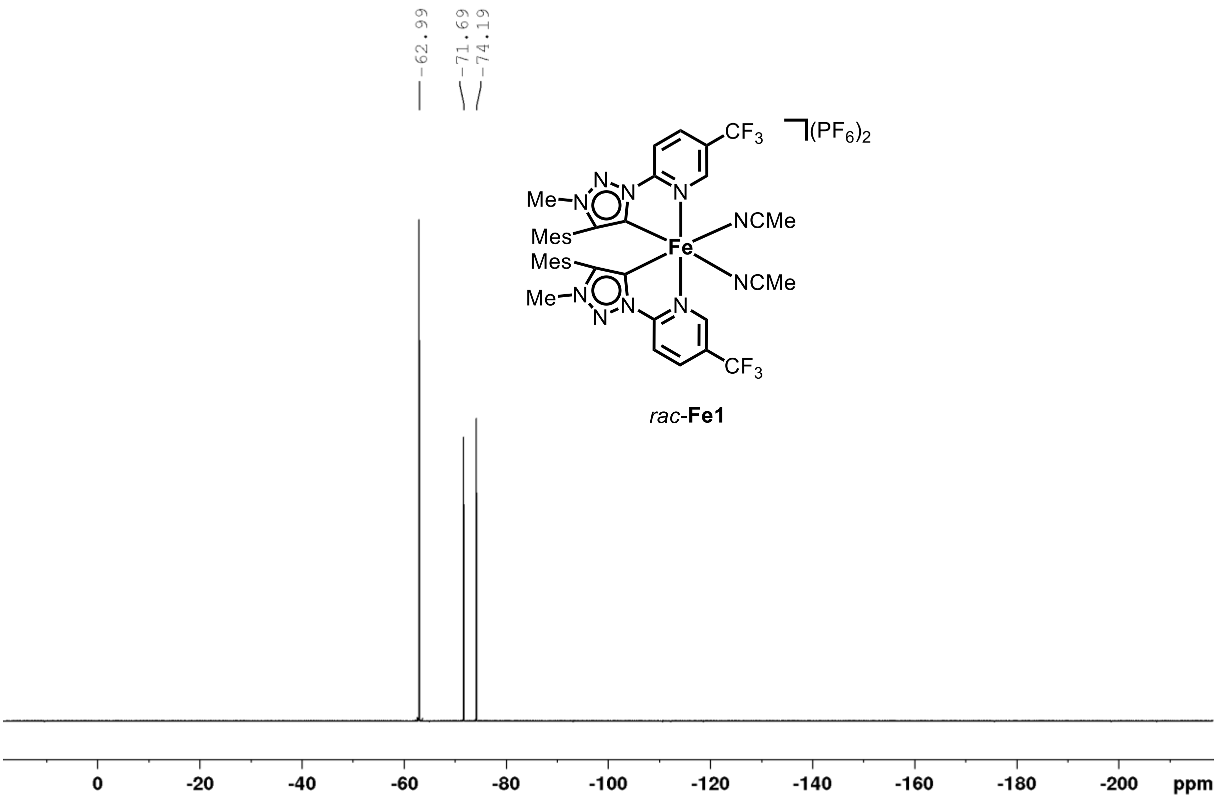

Figure S46. ^{19}F-NMR spectrum of ***rac*-Fe1** (282 MHz, CD_3CN, 300 K).

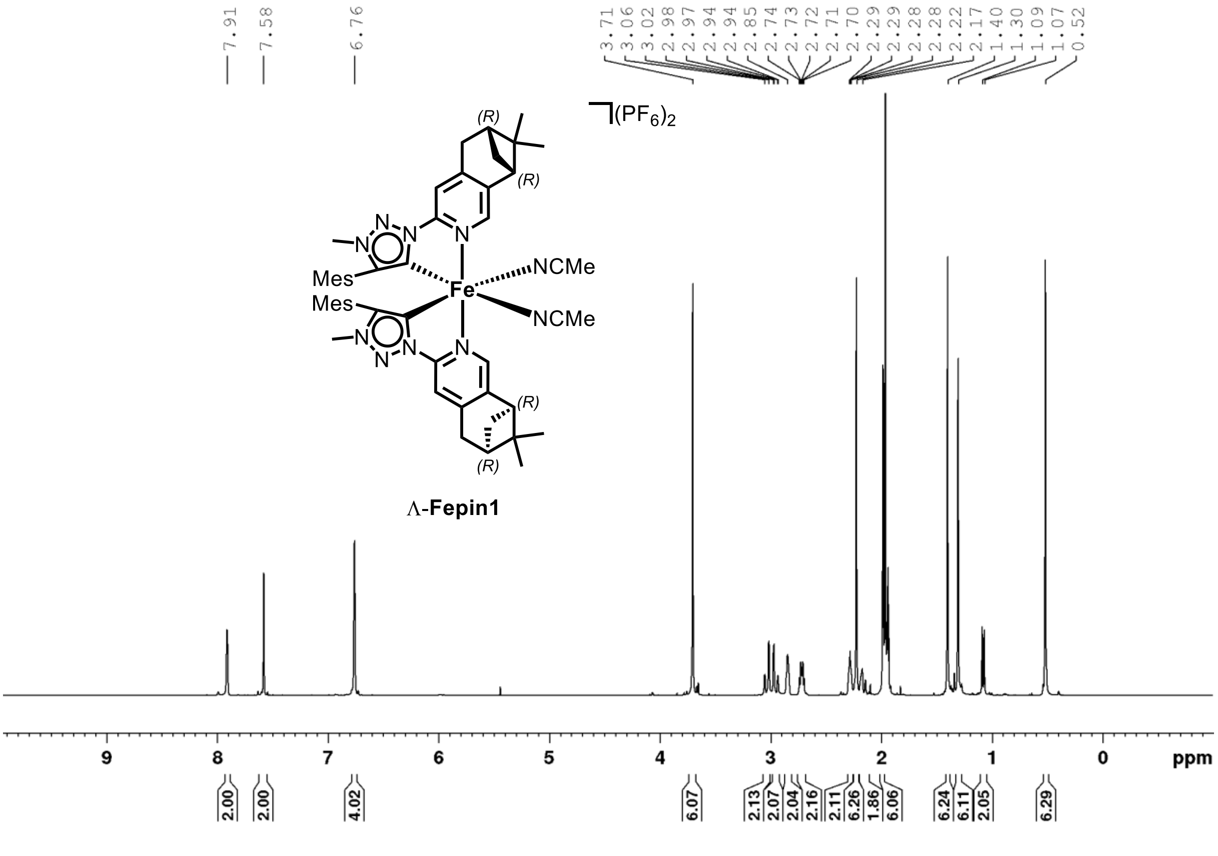

Figure S47. ^{1}H-NMR spectrum of **Λ-Fepin1** (500 MHz, CD_3CN, 300 K).

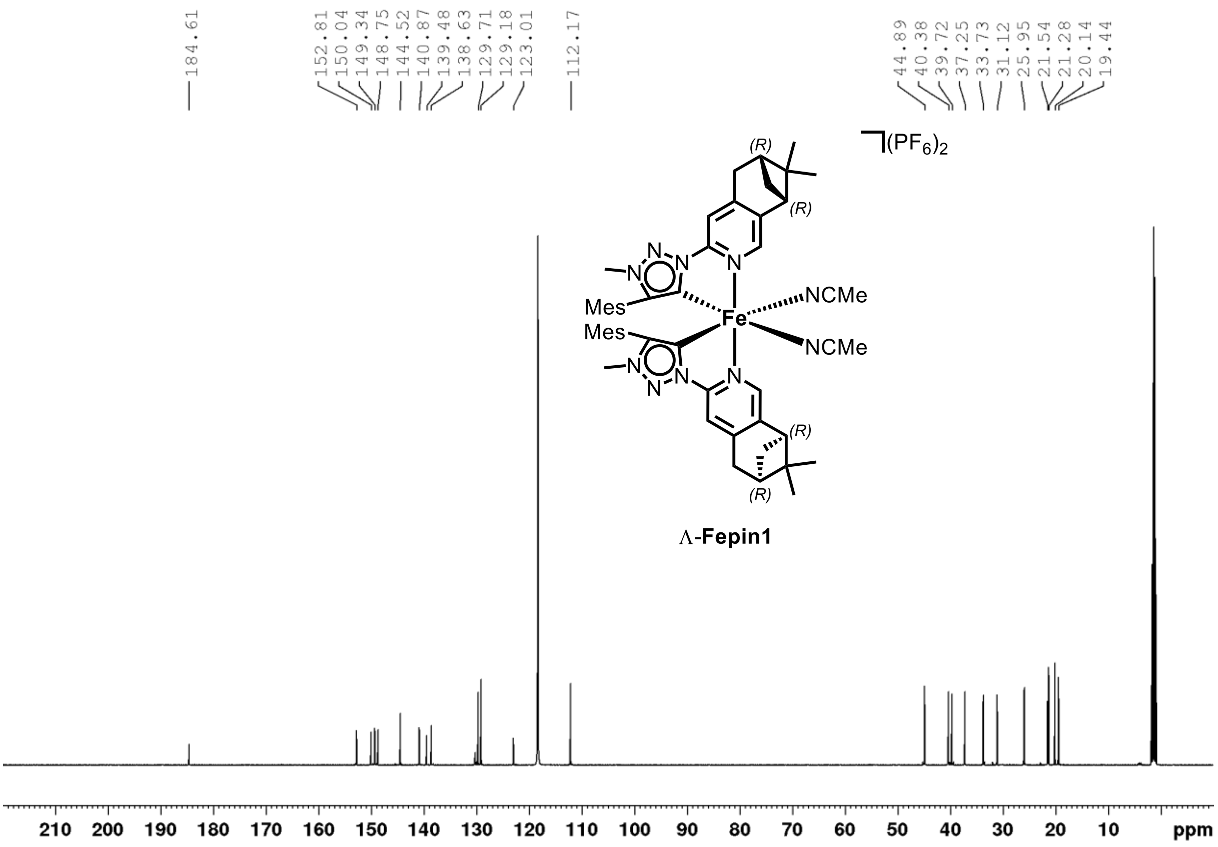

Figure S48. ^{13}C-NMR spectrum of **Λ-Fepin1** (125 MHz, CD_3CN, 300 K).

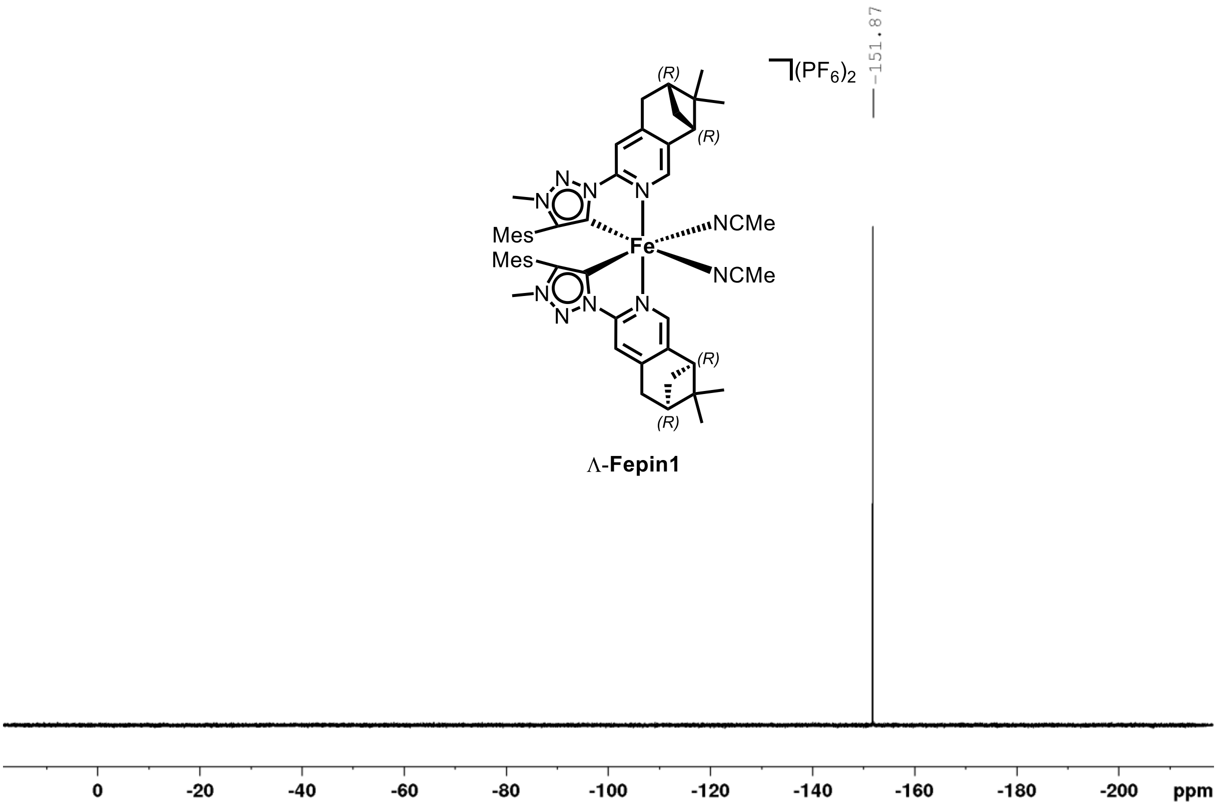

Figure S49. ^{19}F-NMR spectrum of **Λ-Fepin1** (282 MHz, CD_3CN, 300 K).

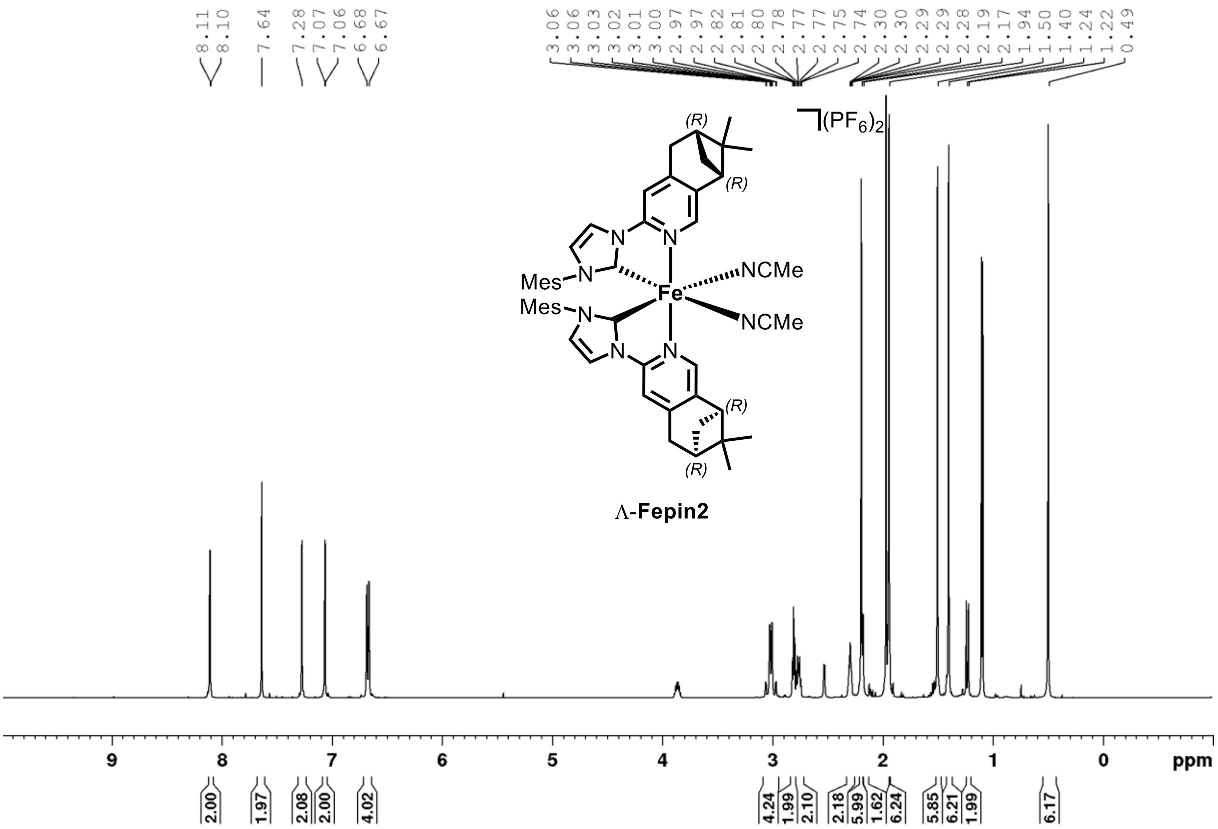

Figure S50. ^{1}H-NMR spectrum of **Λ-Fepin2** (500 MHz, CD_3CN, 300 K).

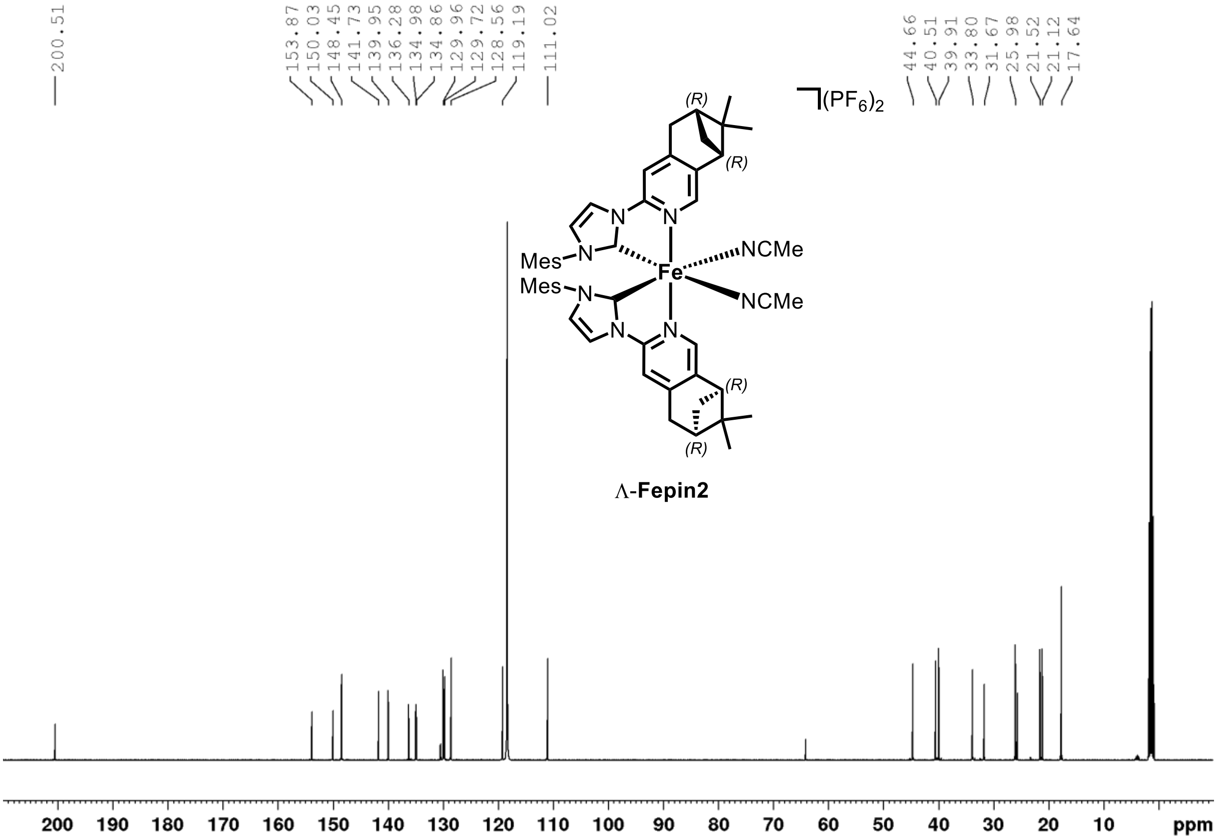

Figure S51. ^{13}C-NMR spectrum of **Λ-Fepin2** (125 MHz, CD_3CN, 300 K).

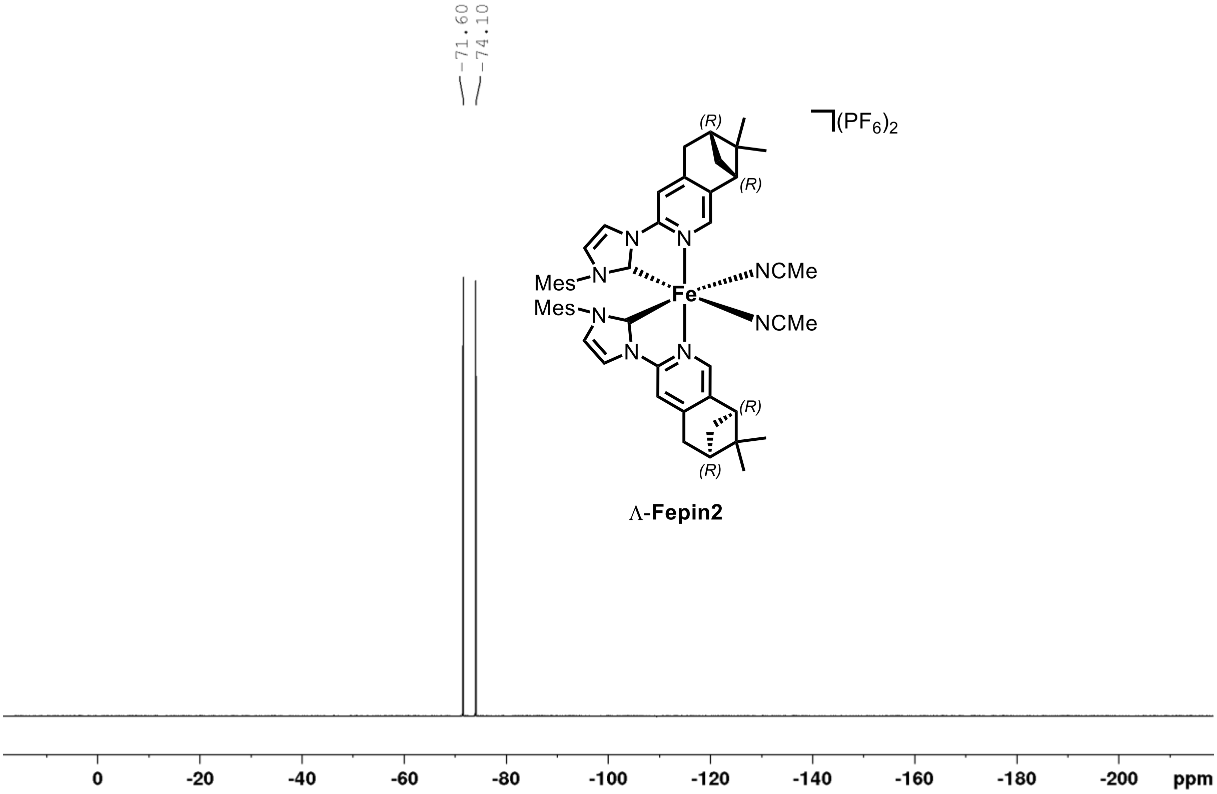

Figure S52. ^{19}F-NMR spectrum of **Λ-Fepin2** (282 MHz, CD_3CN, 300 K).

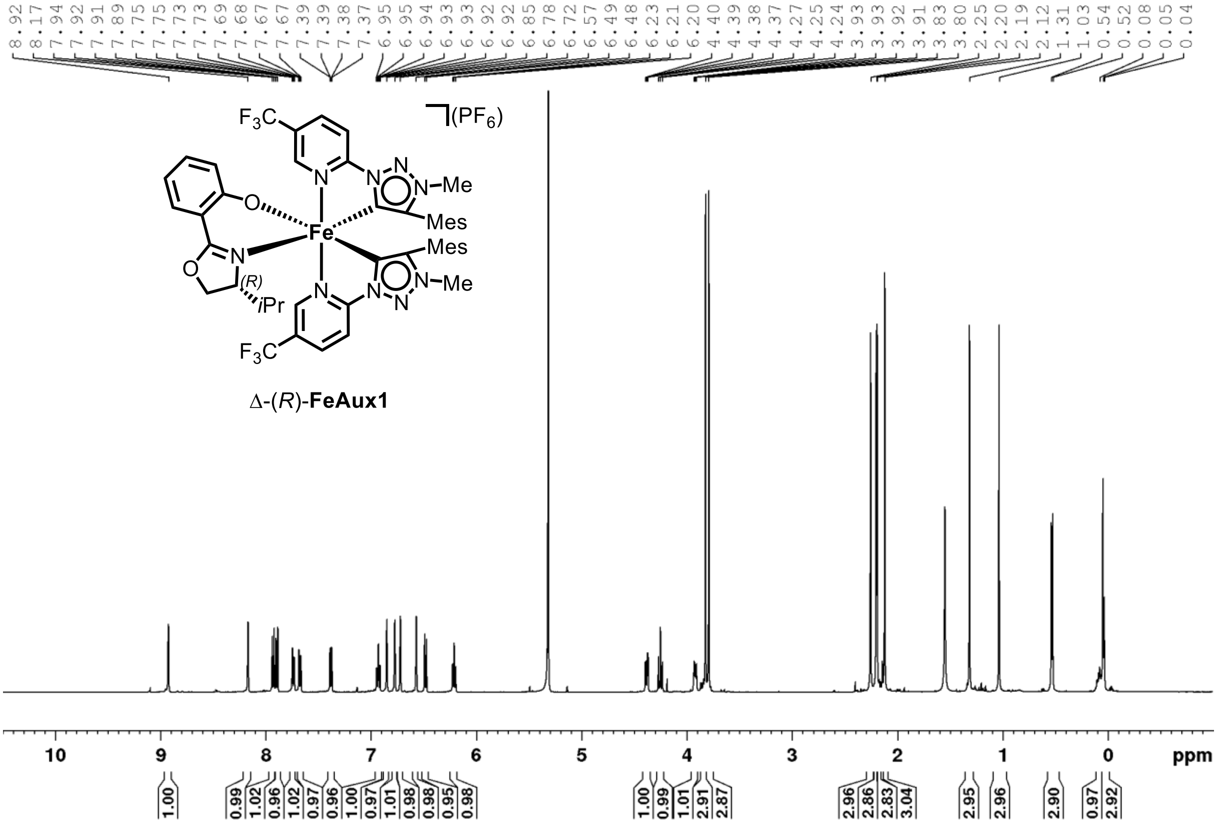

Figure S53. ^{1}H-NMR spectrum of **Δ-(*R*)-FeAux1** (500 MHz, CD_2Cl_2, 300 K).

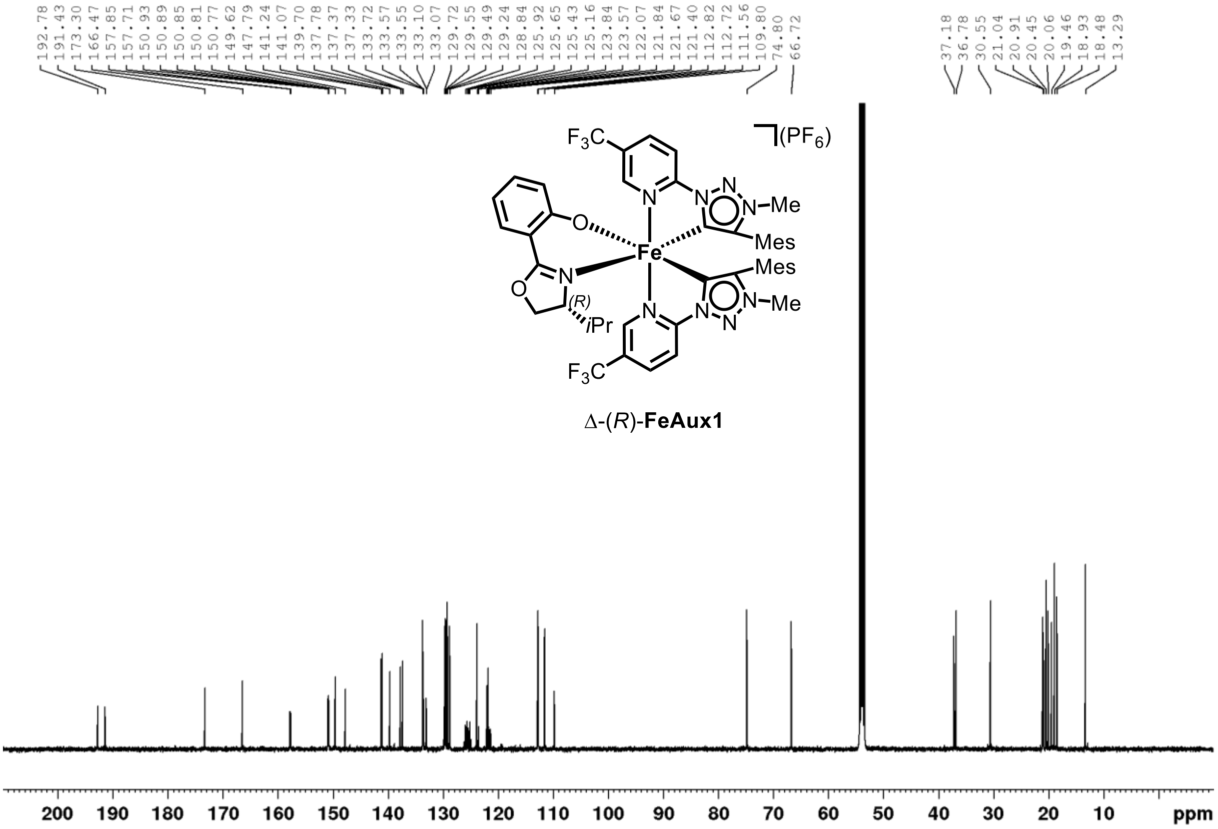

Figure S54. ^{13}C-NMR spectrum of **Δ-(*R*)-FeAux1** (125 MHz, CD_2Cl_2, 300 K).

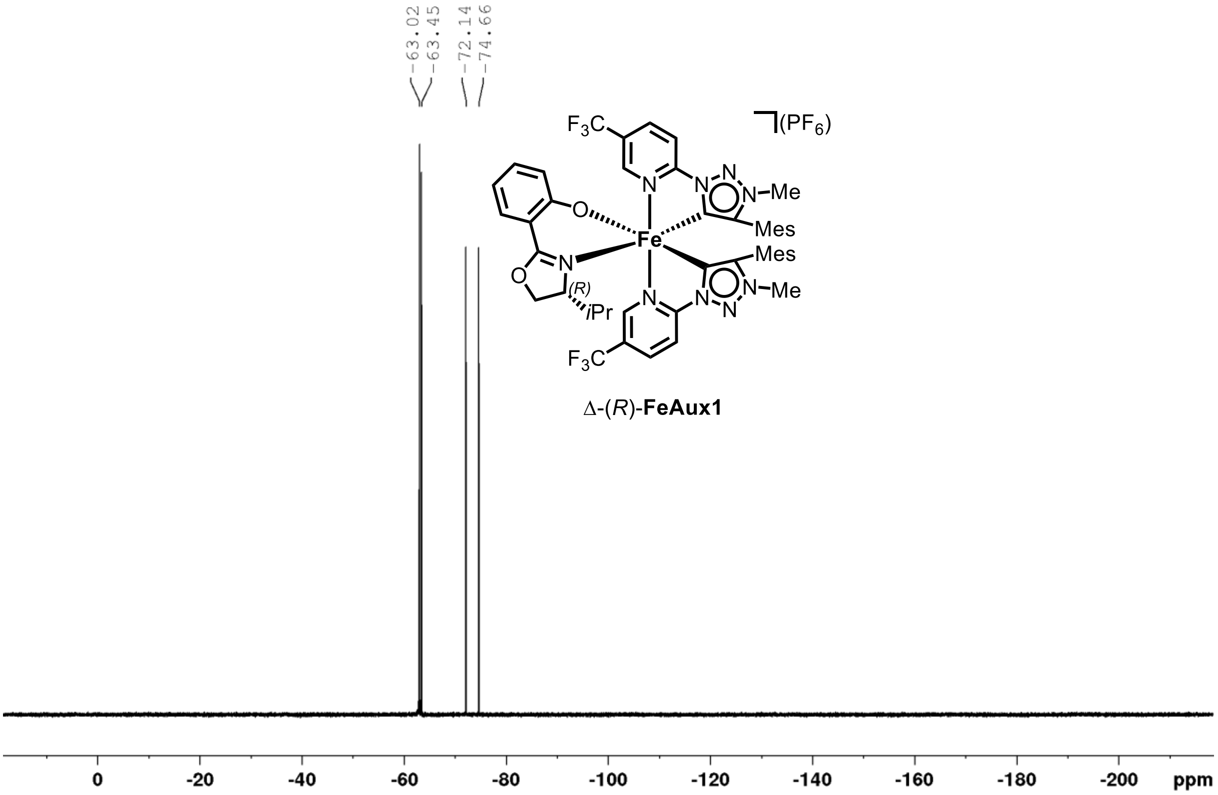

Figure S55. ^{19}F-NMR spectrum of **Δ-(*R*)-FeAux1** (282 MHz, CD_2Cl_2, 300 K).

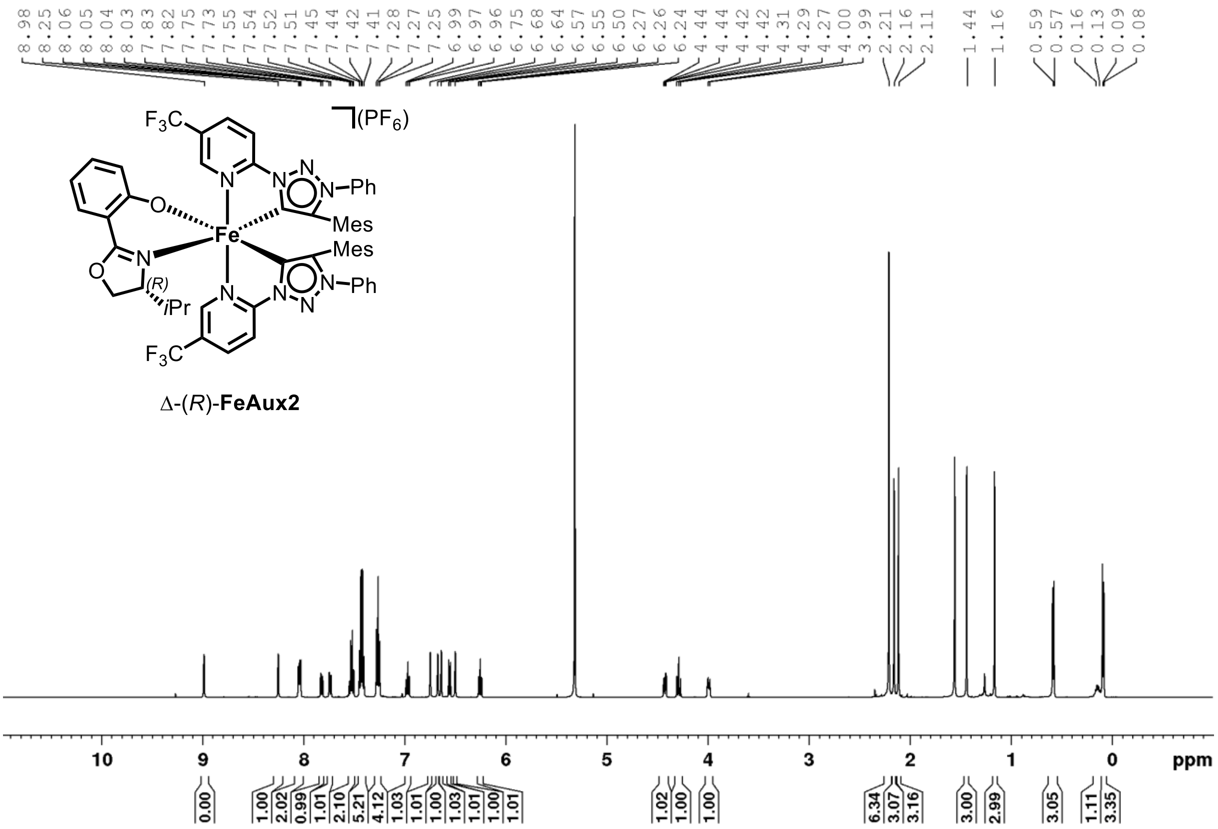

Figure S56. ^{1}H-NMR spectrum of **Δ-(*R*)-FeAux2** (500 MHz, CD_2Cl_2, 300 K).

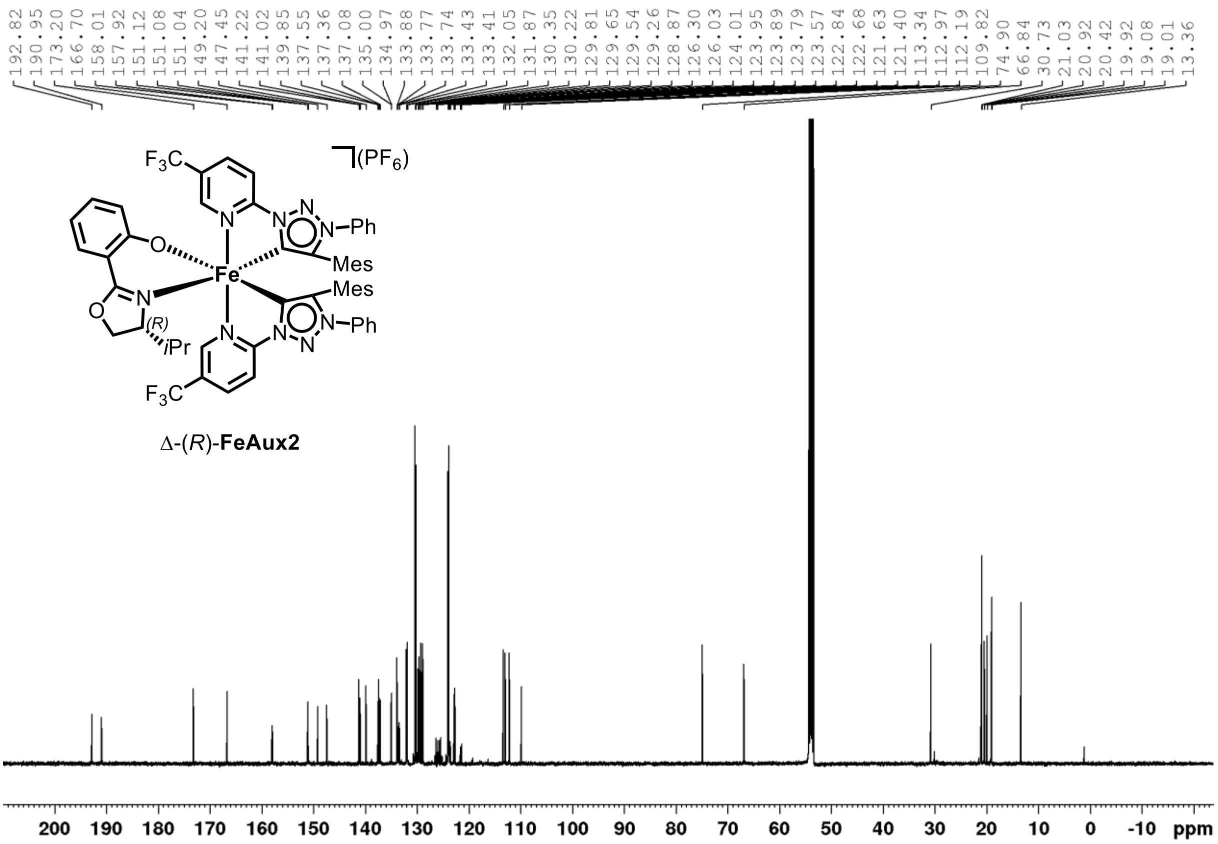

Figure S57. ^{13}C-NMR spectrum of **Δ-(*R*)-FeAux2** (125 MHz, CD_2Cl_2, 300 K).

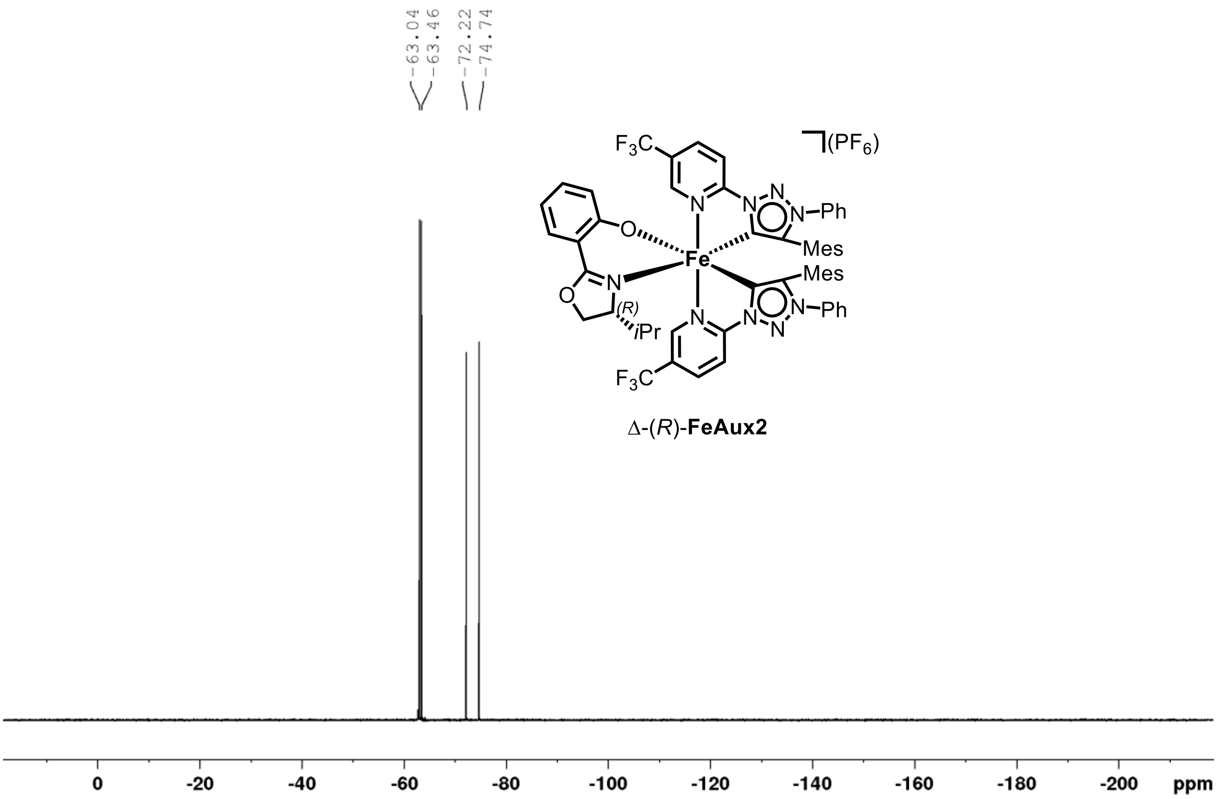

Figure S58. ^{19}F-NMR spectrum of **Δ-(*R*)-FeAux2** (282 MHz, CD_2Cl_2, 300 K).

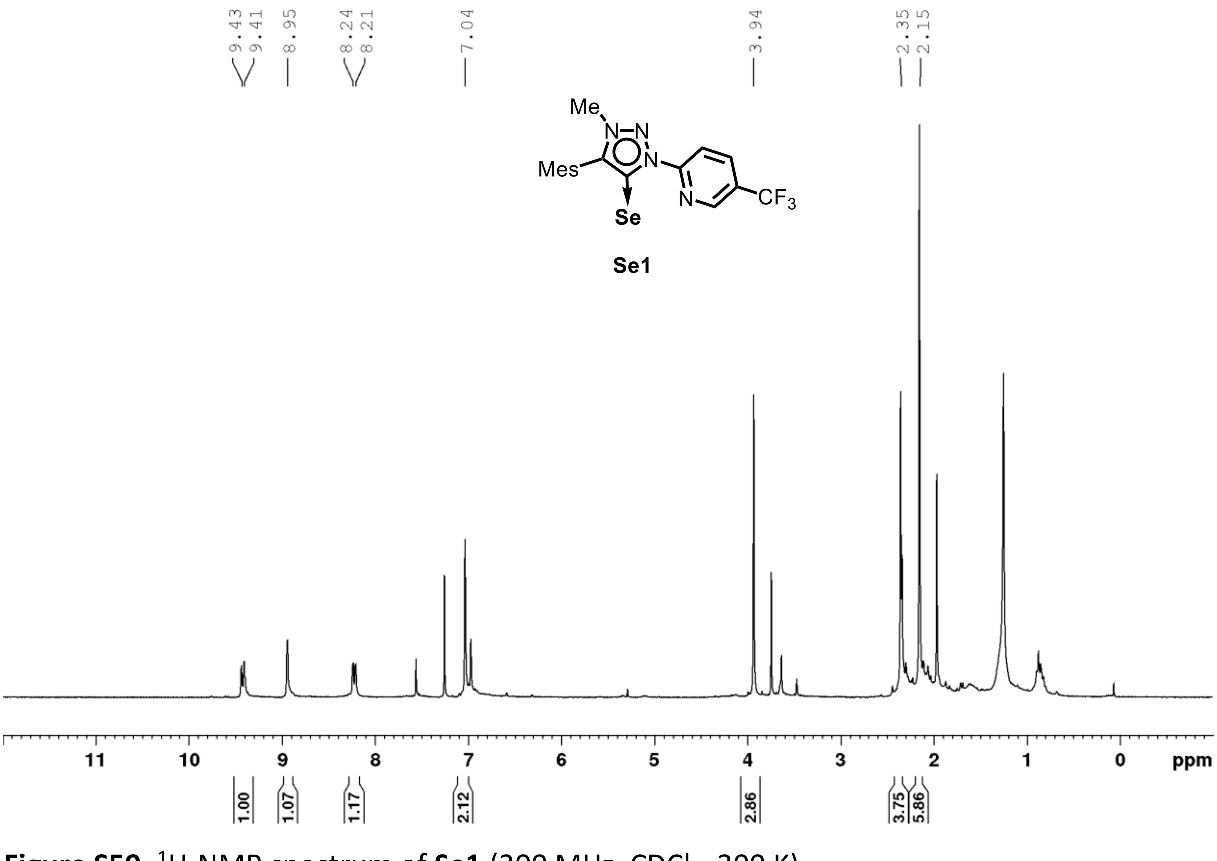

Figure S59. ^{1}H-NMR spectrum of **Se1** (300 MHz, $CDCl_3$, 300 K).

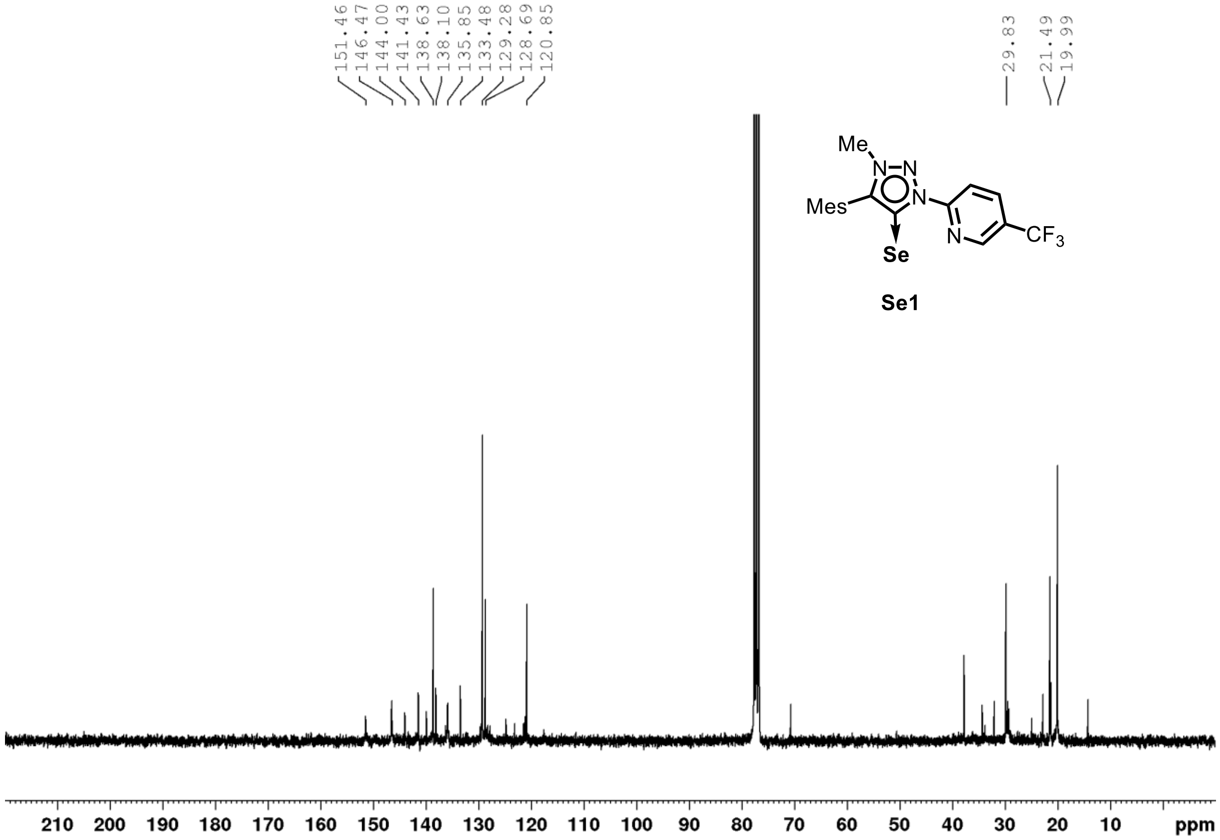

Figure S60. ^{13}C-NMR spectrum of **Se1** (75 MHz, $CDCl_3$, 300 K).

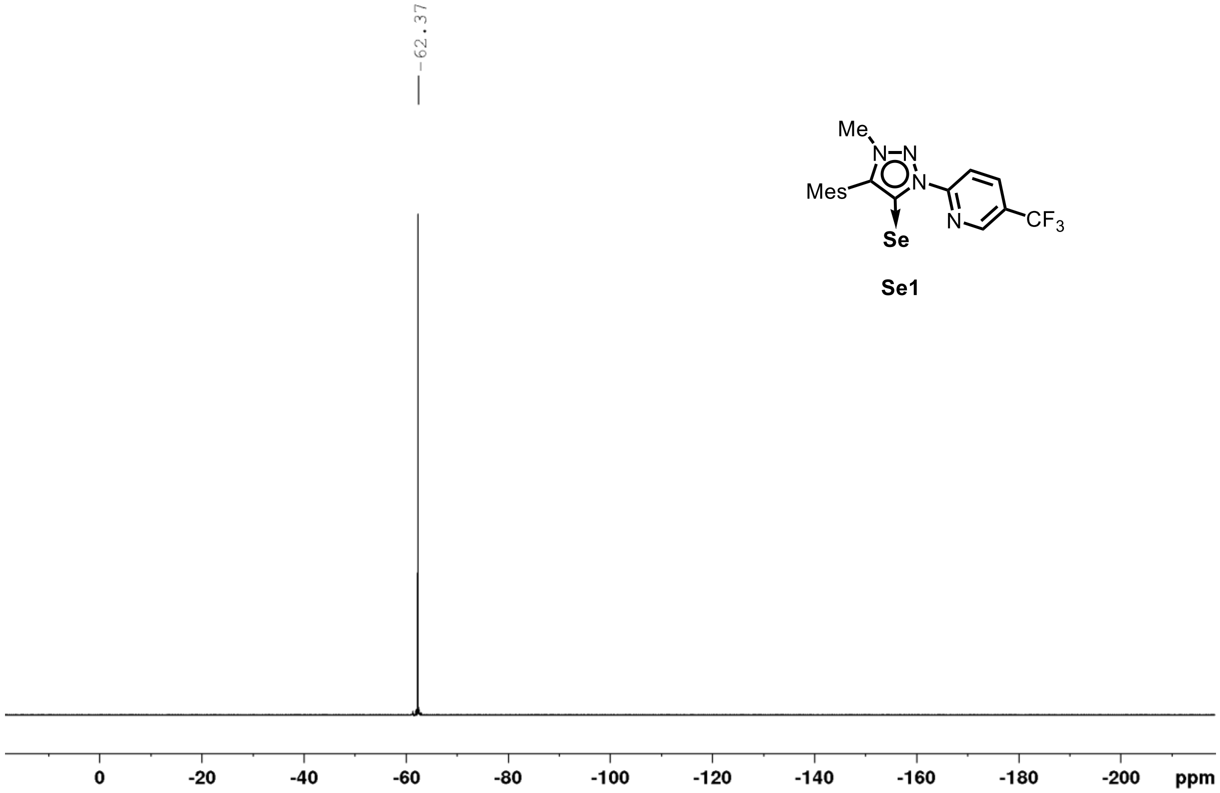

Figure S61. ^{19}F-NMR spectrum of **Se1** (282 MHz, $CDCl_3$, 300 K).

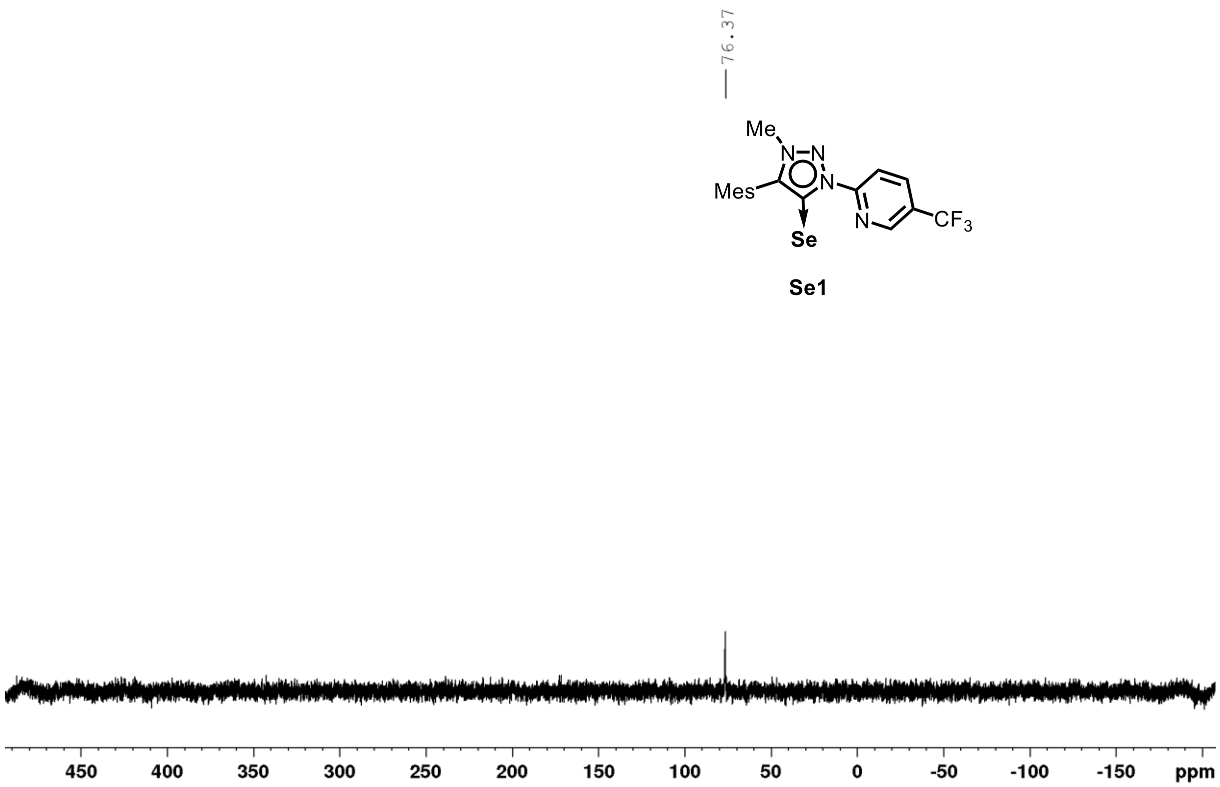

Figure S62. ^{77}Se-NMR spectrum of **Se1** (95 MHz, $CDCl_3$, 300 K).

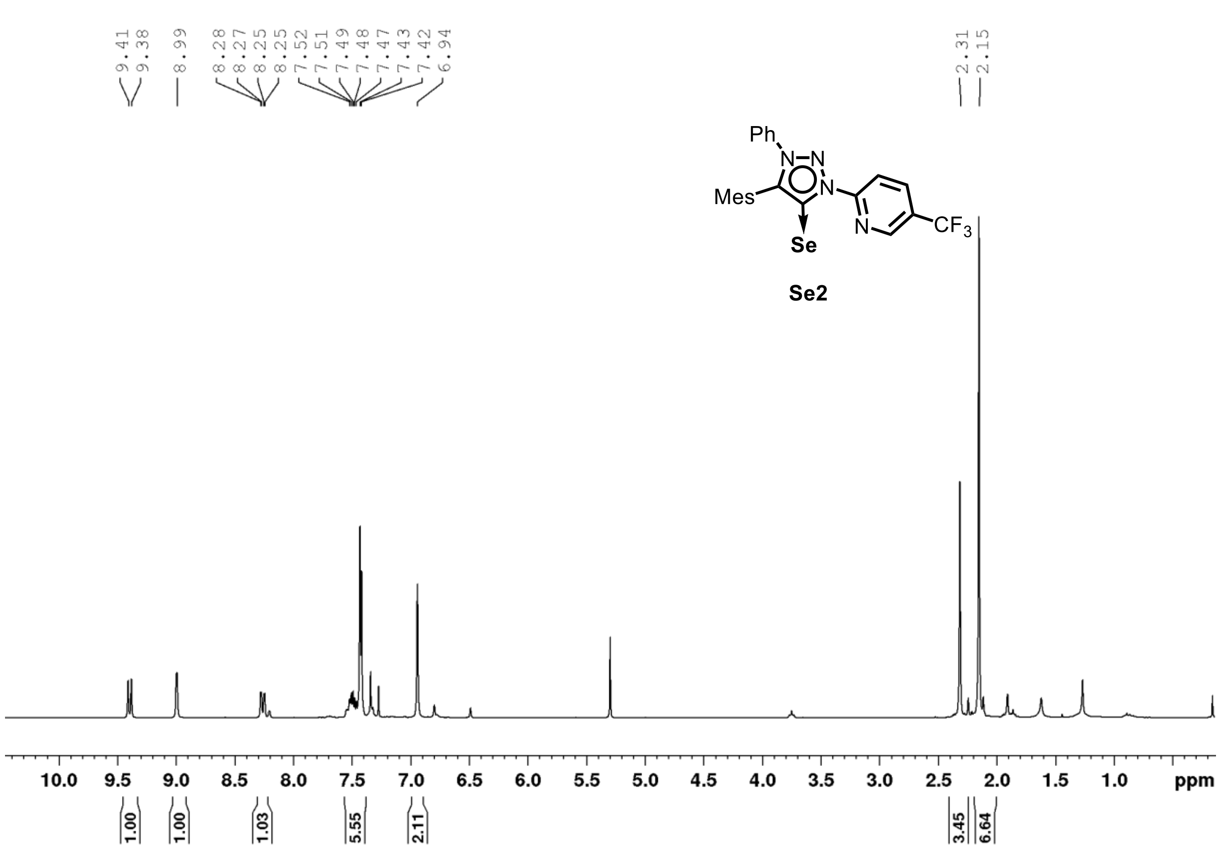

Figure S63. ^{1}H-NMR spectrum of **Se2** (300 MHz, $CDCl_3$, 300 K).

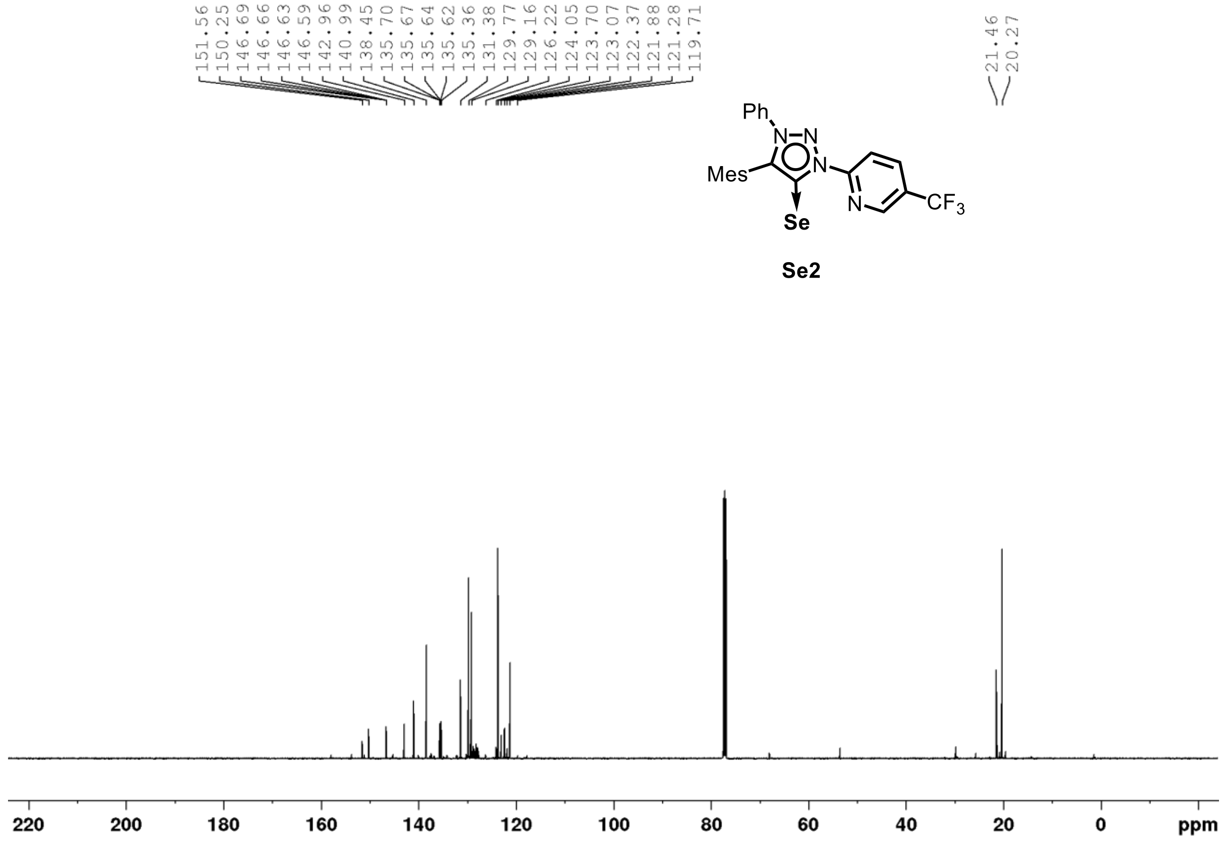

Figure S64. ^{13}C-NMR spectrum of **Se2** (125 MHz, $CDCl_3$, 300 K).

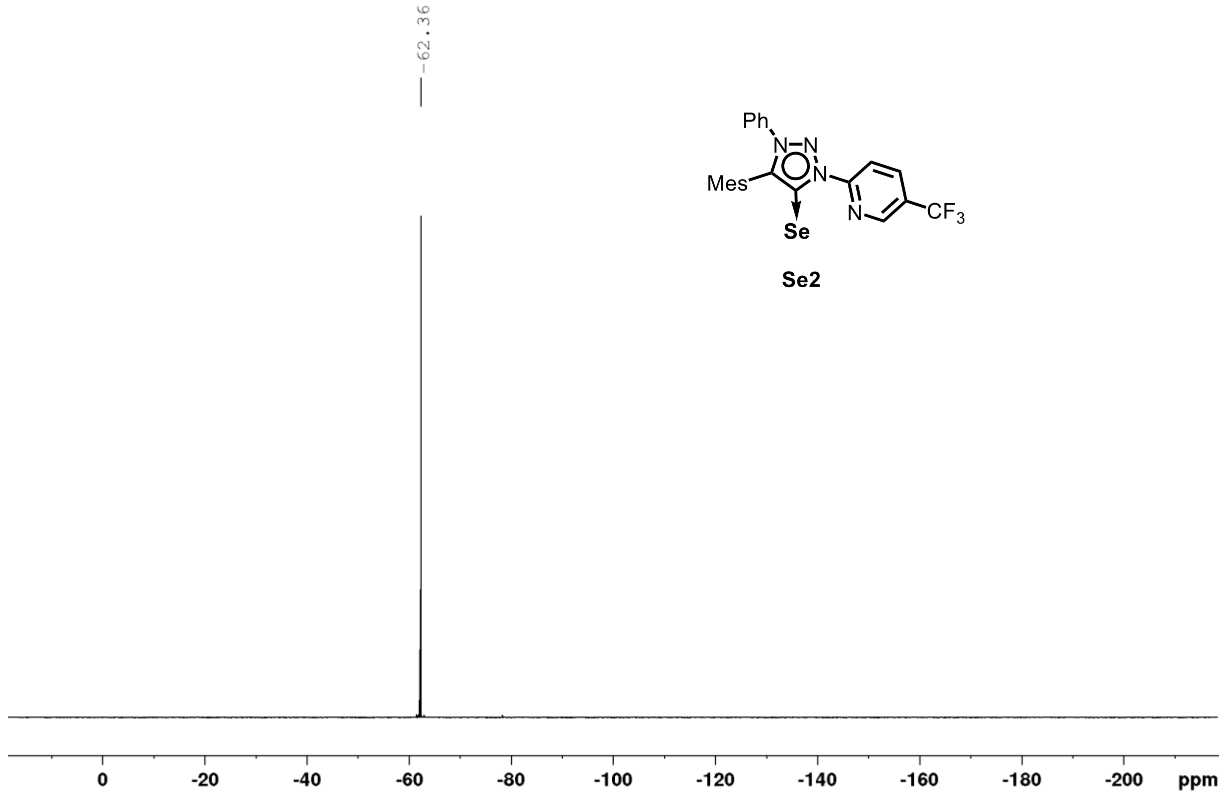

Figure S65. ^{19}F-NMR spectrum of **Se2** (282 MHz, $CDCl_3$, 300 K).

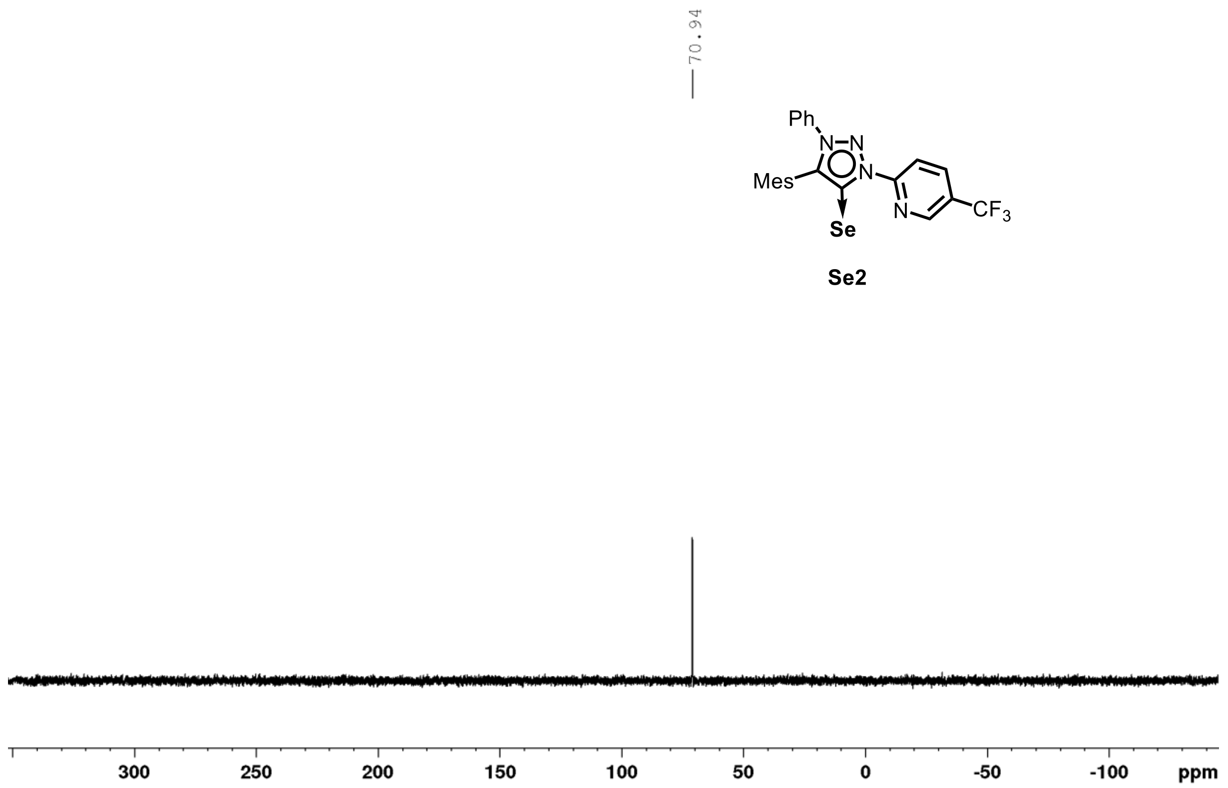

Figure S66. ^{77}Se-NMR spectrum of **Se2** (95 MHz, $CDCl_3$, 300 K).

11. Chiral HPLC Traces

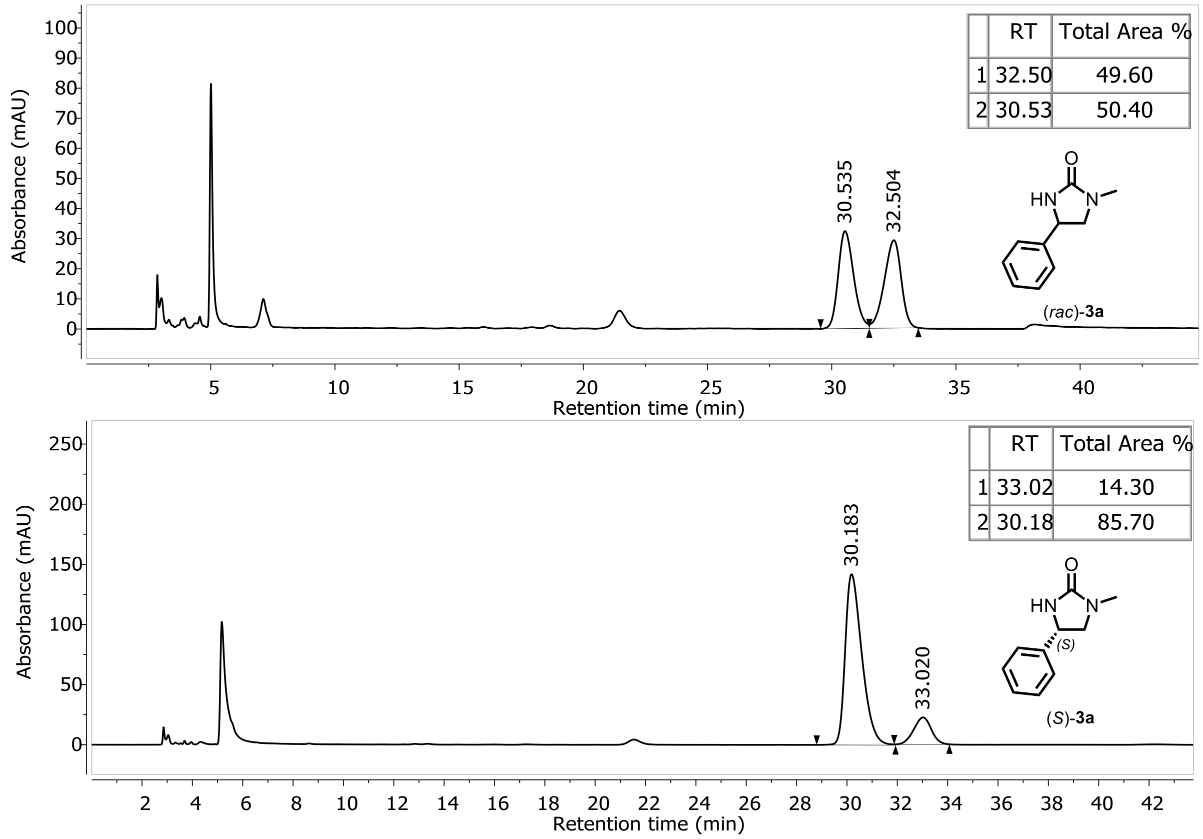

Figure S67. HPLC chromatogram of <u>up</u>: *rac*-**3a** and <u>down</u>: (*S*)-**3a** with (86:14 er) at –10 °C.

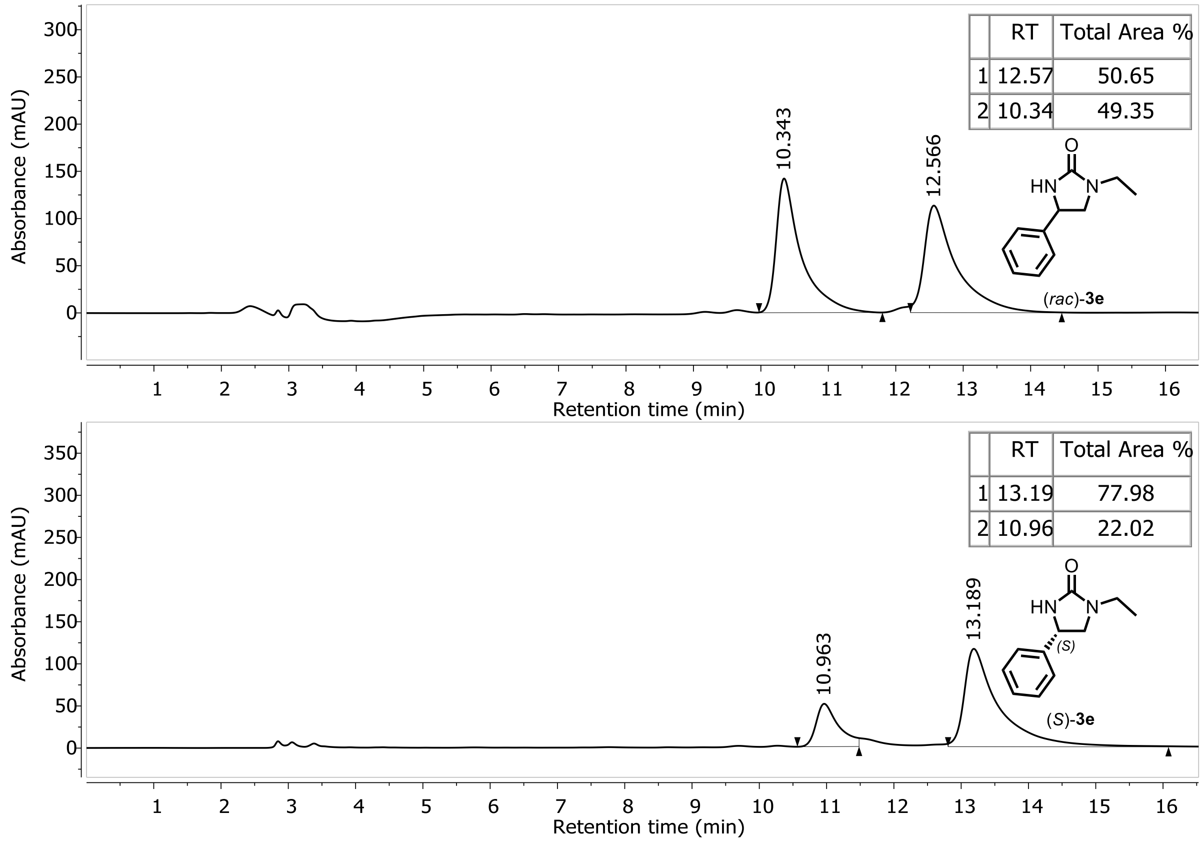

Figure S68. HPLC chromatogram of <u>up</u>: *rac*-**3e** and <u>down</u>: (*S*)-**3e** with (78:22 er) at 25 °C.

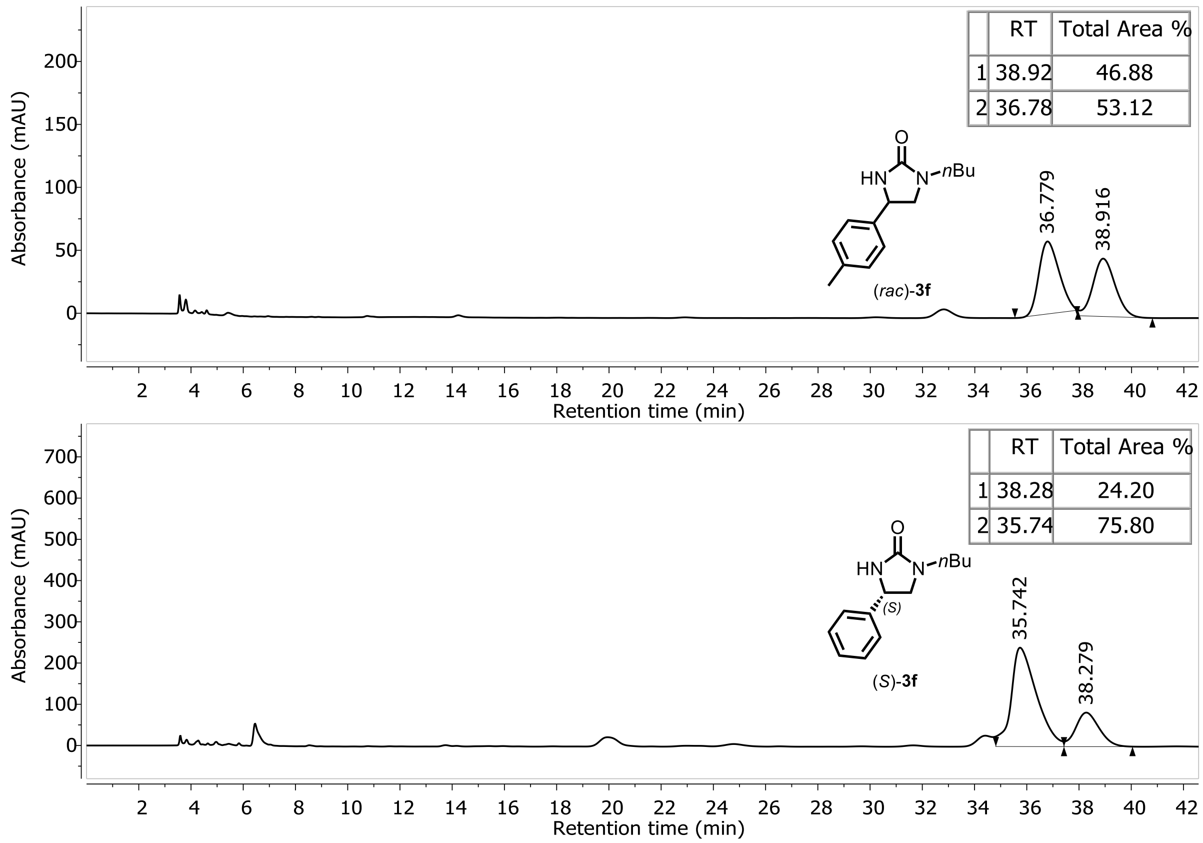

Figure S69. HPLC chromatogram of up: *rac*-**3f** and down: (*S*)-**3f** with (76:24 er) at 25 °C.

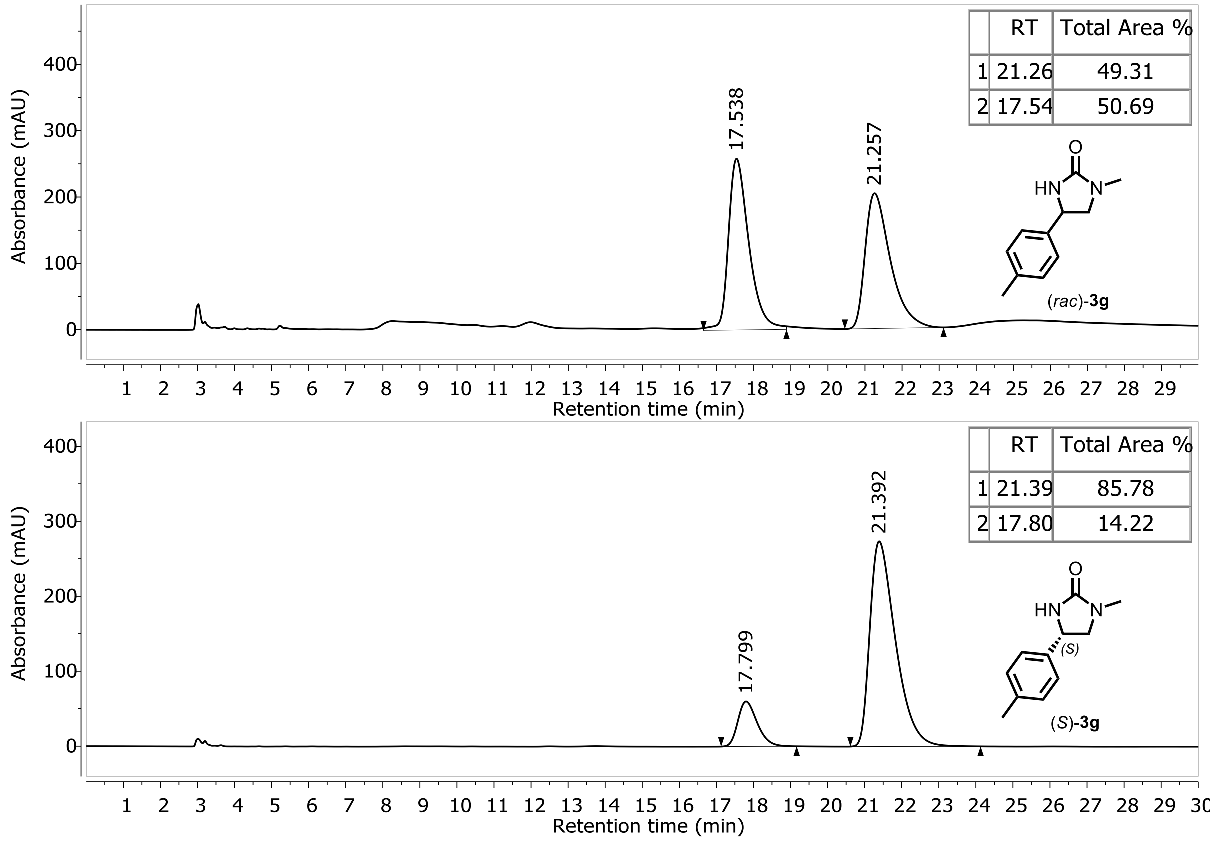

Figure S70. HPLC chromatogram of up: *rac*-**3g** and down: (*S*)-**3g** with (86:14 er) at 4 °C.

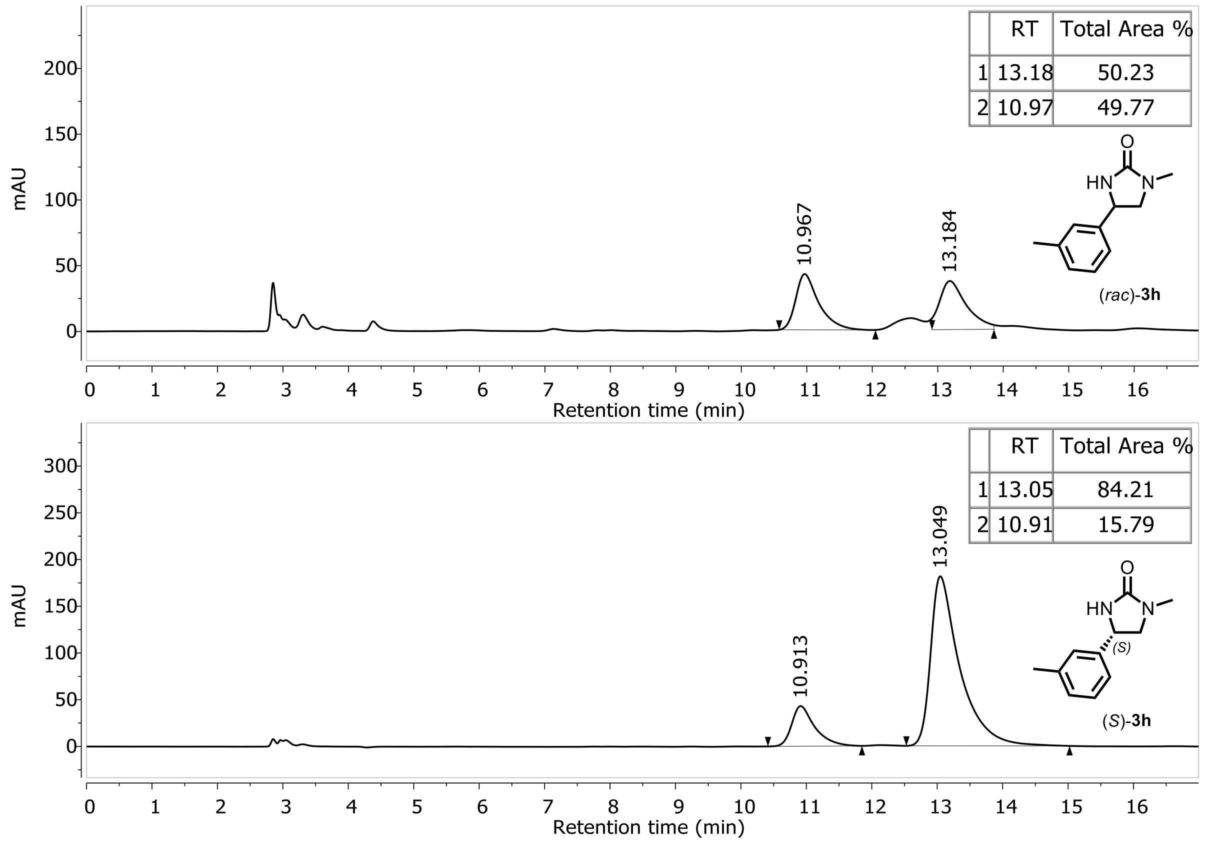

Figure S71. HPLC chromatogram of up: *rac*-**3h** and down: (*S*)-**3h** with (84:16 er) at 4 °C.

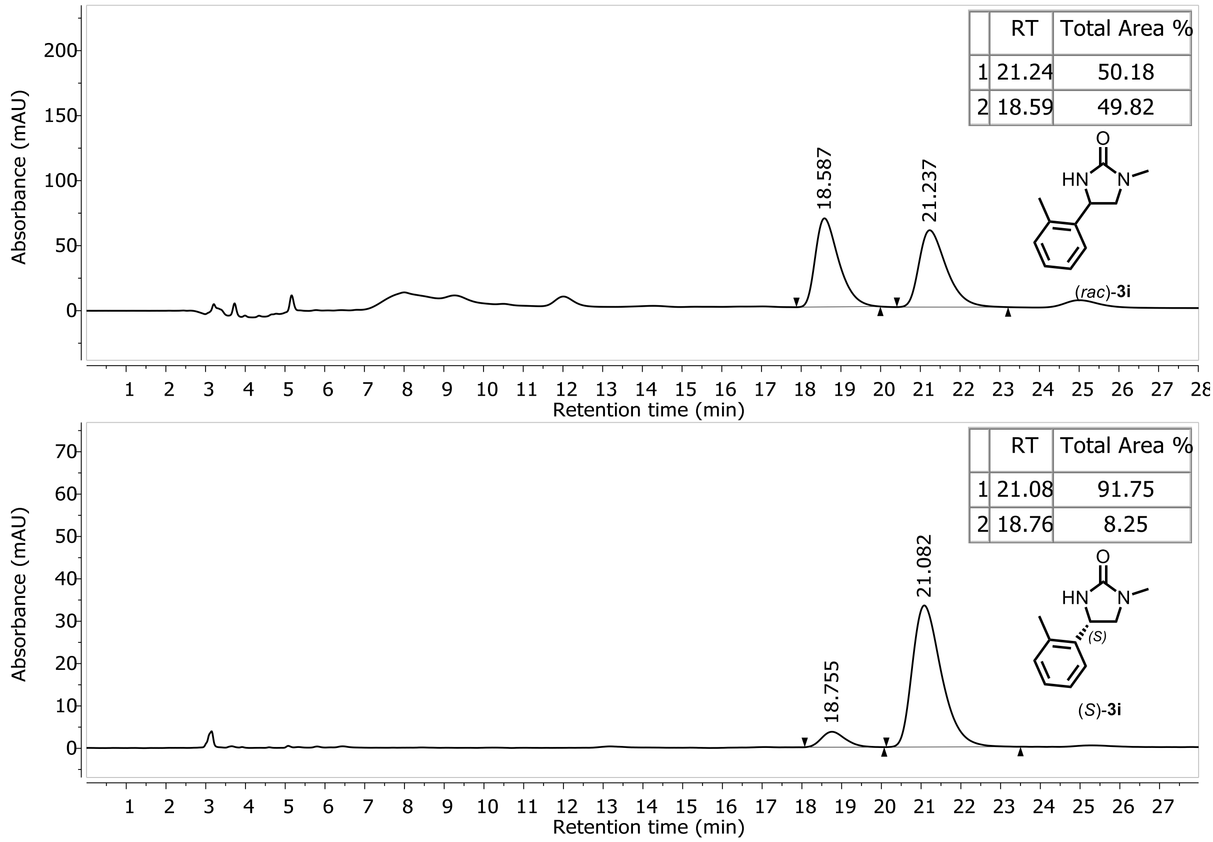

Figure S72. HPLC chromatogram of up: *rac*-**3i** and down: (*S*)-**3i** with (92:8 er) at –10 °C.

12. CD-Spectra

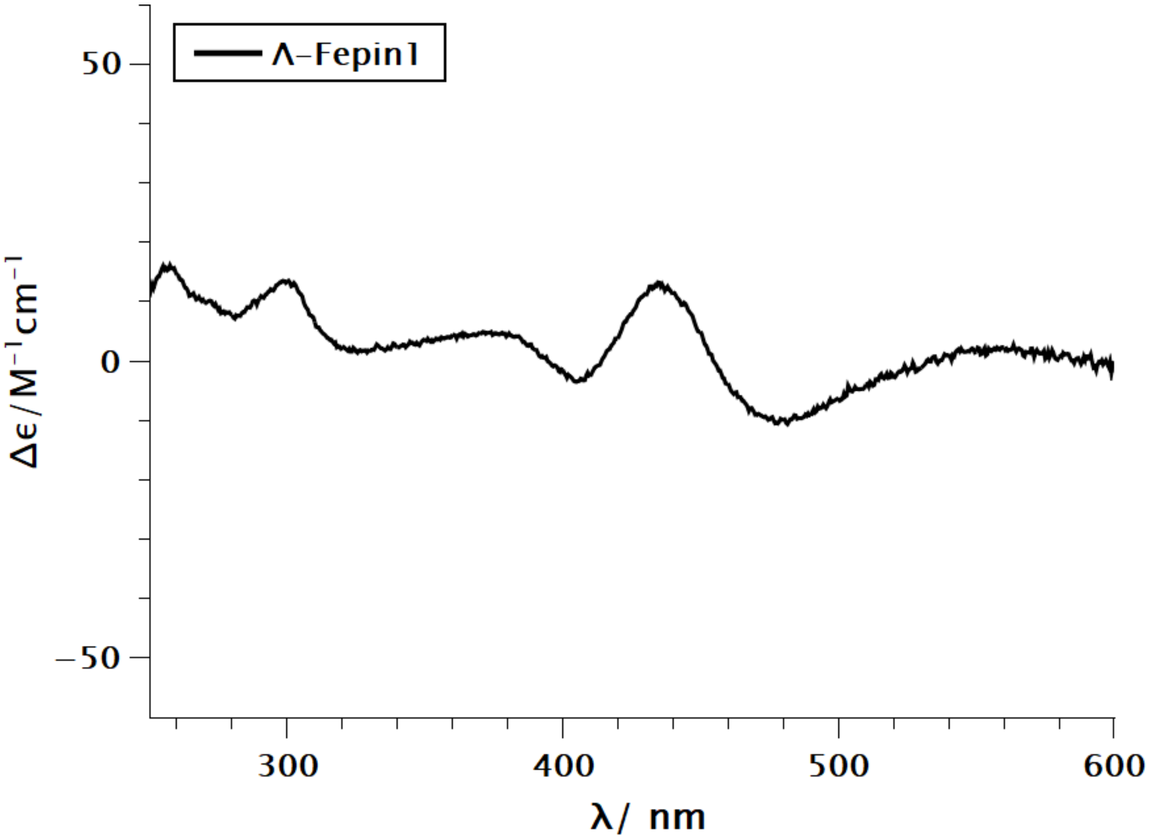

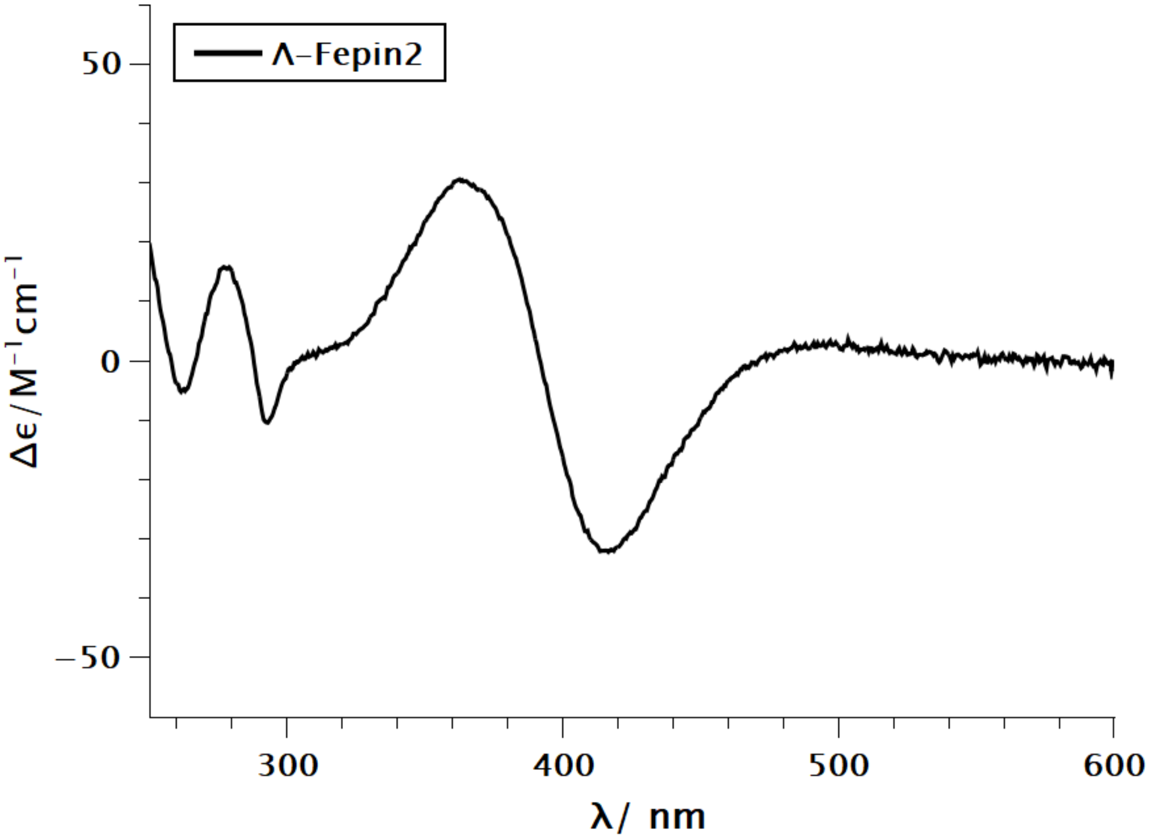

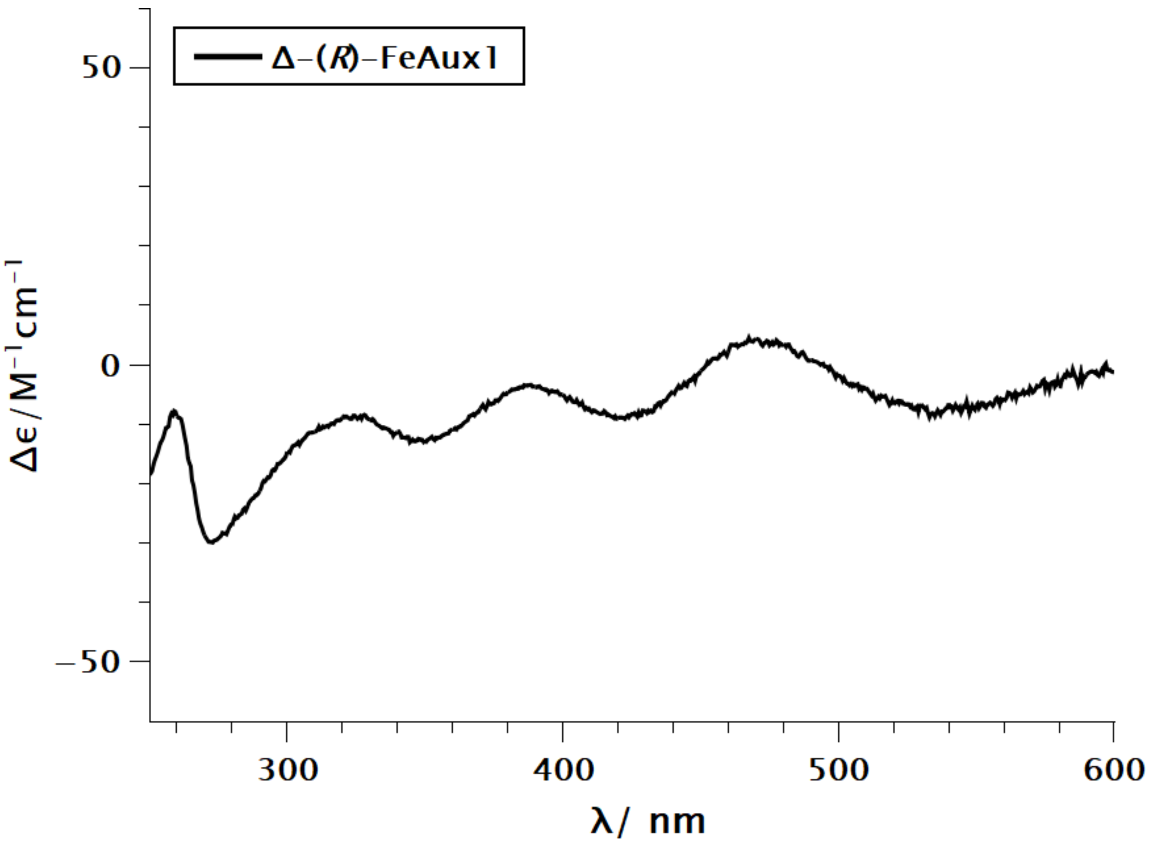
Δ-(*R*)-FeAux1
50
0
−50
Δε/M⁻¹cm⁻¹
300
400
500
600
λ/ nm

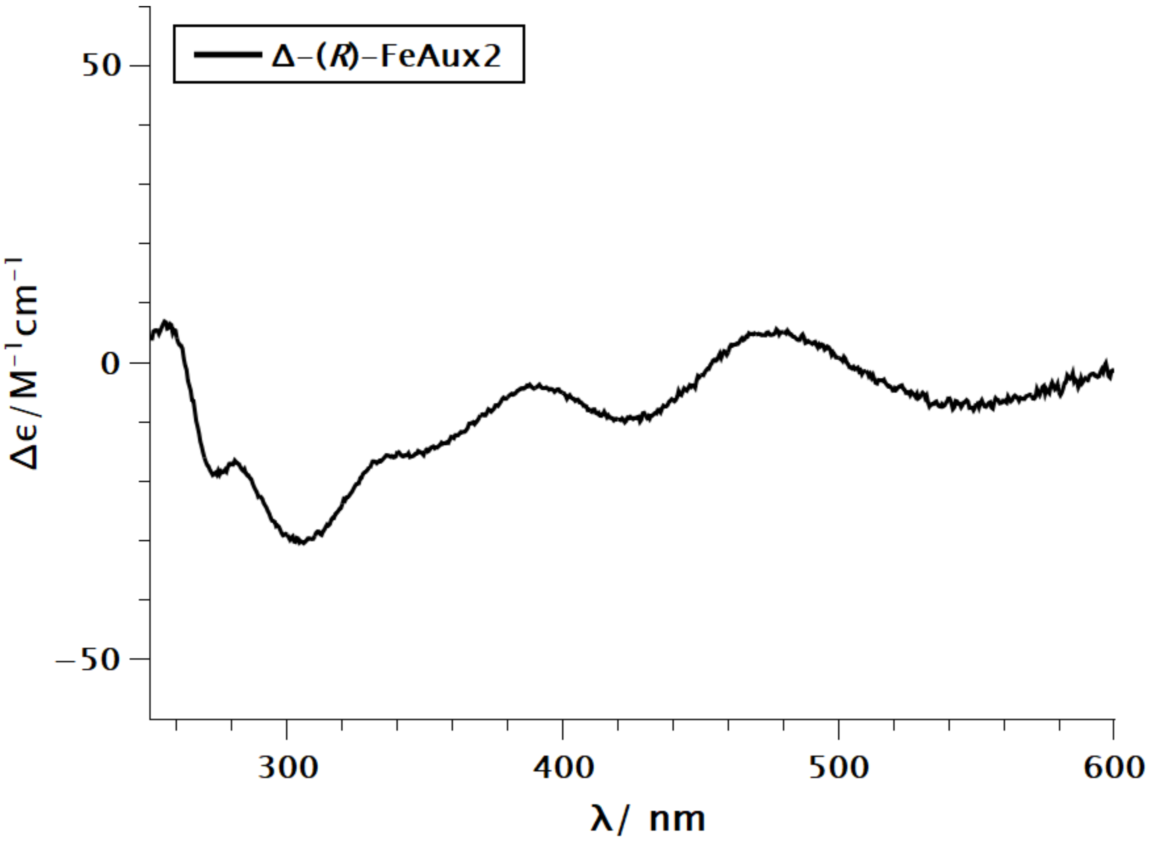
Δ-(*R*)-FeAux2
50
0
−50
Δε/M⁻¹cm⁻¹
300
400
500
600
λ/ nm

13. UV-Visible Spectra

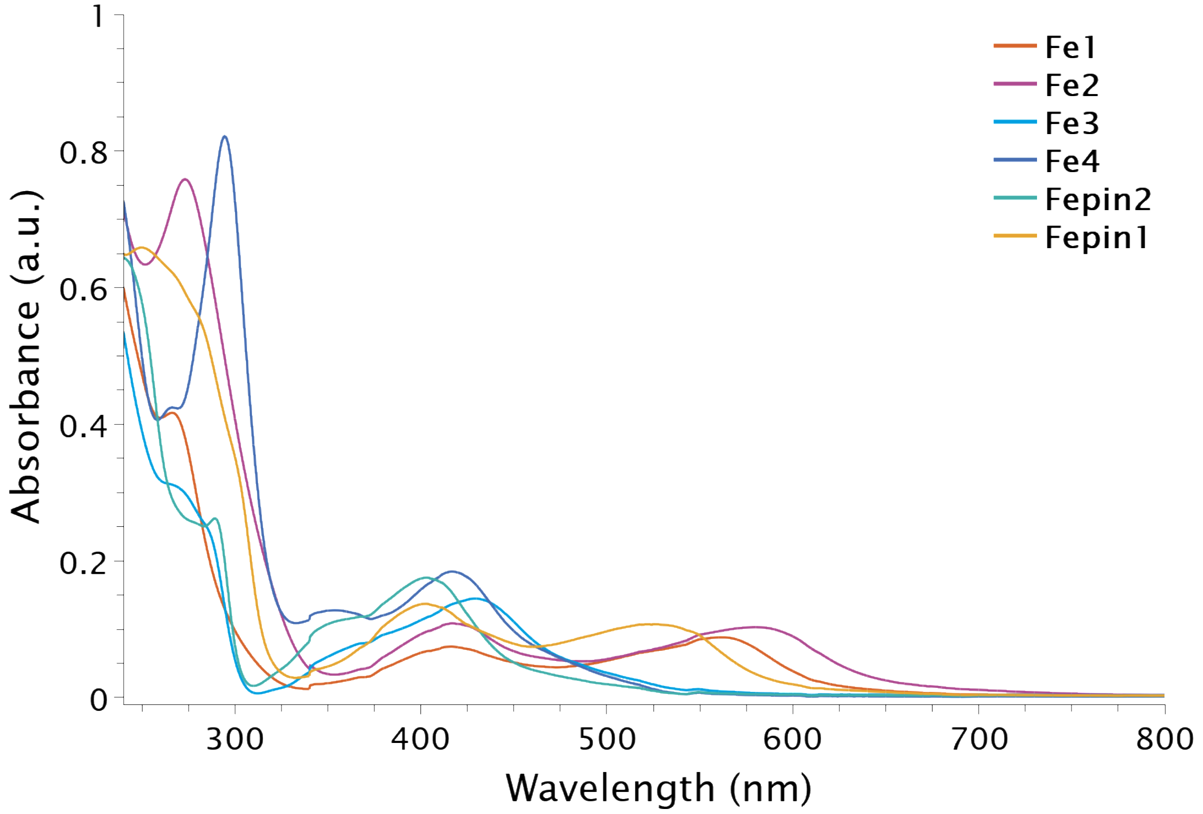

Figure S73. UV/Vis-absorbance measurements. UV/Vis absorption spectra of iron complexes **Fe1**-**4** and **Fepin1+2** in MeCN (0.02 mM, 25 °C).

Table S2. Absorption maxima λ_1-λ_3 for the iron complexes **Fe1-4** and **Fepin1+2**.

	Fe1	Fe2	Fe3	Fe4	Fepin1	Fepin2
λ_1 [nm]	270	274	288	294	284	290
λ_2 [nm]	416	418	368	354	403	358
λ_3 [nm]	563	582	430	417	527	403

14. Single Crystal X-Ray Diffraction

rac-**Fe1**

A suitable crystal of $C_{40}H_{40}F_6FeN_{10}(PF_6)_2$ was selected under inert oil and mounted using a MiTeGen loop. Intensity data of the crystal were recorded with a D8 Quest diffractometer (Bruker AXS). The instrument was operated with Mo-Kα radiation (0.71073 Å, microfocus source) and equipped with a PHOTON III C14 detector. Evaluation, integration and reduction of the diffraction data was carried out using the Bruker APEX 3 software suite.[20] Multi-scan and numerical absorption corrections were applied using the SADABS program.[21,22] The structure was solved using dual-space methods (SHELXT-2018/2) and refined against F2 (SHELXL-2018/3 using ShelXle interface).[23–25] All non-hydrogen atoms were refined with anisotropic displacement parameters. The hydrogen atoms were refined using the "riding model" approach with isotropic displacement parameters 1.2 times (1.5 times for terminal methyl groups) of that of the preceding carbon atom. CCDC 2346495 contains the supplementary crystallographic data for this paper. These data can be obtained free of charge from The Cambridge Crystallographic Data Centre via www.ccdc.cam.ac.uk/structures.

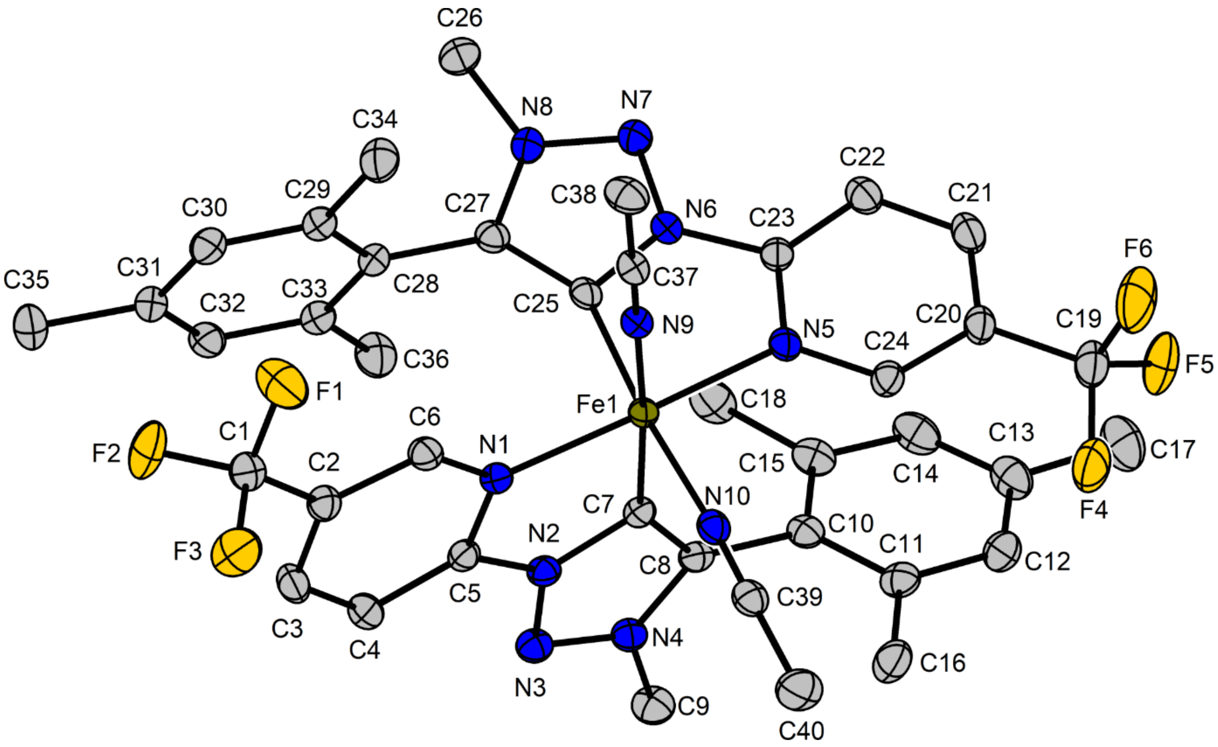

Figure S74. Crystal structure of product *rac*-**Fe1**. Only one enantiomer is shown. The $[PF_6]^-$ anions and the hydrogen atoms are not shown. Disorder is not shown. Displacement ellipsoids are shown for clarity at 50 % probability level at 100 K.

Table S3. Selected crystallographic data and details of the structure determination for $C_{40}H_{40}F_6FeN_{10}(PF_6)_2$.

Identification code	iND049
Empirical formula	$C_{40}H_{40}F_{18}FeN_{10}P_2$
Molar mass / g·mol^{-1}	1120.61
Space group (No.)	$P2_1/c$ (14)
a / Å	12.6131(5)
b / Å	23.9374(9)
c / Å	16.1455(7)
β / °	109.5190(10)
V / Å^3	4594.6(3)
Z	4
$\rho_{calc.}$ / g·cm^{-3}	1.620
μ / mm^{-1}	0.515
Color	dark red
Crystal habitus	block

Crystal size / mm^3	0.254 x 0.188 x 0.141
T / K	100
λ / Å	0.71073 (Mo-K_α)
θ range / °	2.165 to 28.313
Range of Miller indices	$-16 \leq h \leq 16$
	$-31 \leq k \leq 31$
	$-21 \leq l \leq 21$
Absorption correction	multi-scan and numerical
T_{min}, T_{max}	0.8363, 0.9693
R_{int}, R_σ	0.0509, 0.0296
Completeness of the data set	0.999
No. of measured reflections	104762
No. of independent reflections	11416
No. of parameters	650
No. of restraints	0
S (all data)	1.026
$R(F)$ ($I \geq 2\sigma(I)$, all data)	0.0370, 0.0498
$wR(F^2)$ ($I \geq 2\sigma(I)$, all data)	0.0791, 0.0842
Extinction coefficient	not refined
$\Delta\rho_{max}$, $\Delta\rho_{min}$ / e·Å^{-3}	0.466, –0.450

rac-**Fe2**

A suitable crystal of $C_{50}H_{44}F_6FeN_{10} \cdot 2\ PF_6 \cdot 3\ CH_2Cl_2$ was selected under inert oil and mounted using a MiTeGen loop. Intensity data of the crystal were recorded with a D8 Quest diffractometer (Bruker AXS). The instrument was operated with Mo-Kα radiation (0.71073 Å, microfocus source) and equipped with a PHOTON III C14 detector. Evaluation, integration and reduction of the diffraction data was carried out using the Bruker APEX 3 software suite.[20] Multi-scan and numerical absorption corrections were applied using the SADABS program.[21,22] The structure was solved using dual-space methods (SHELXT-2018/2) and refined against F2 (SHELXL-2018/3 using ShelXle interface).[23–25] All non-hydrogen atoms were refined with anisotropic displacement parameters. The hydrogen atoms were refined using the "riding model" approach with isotropic displacement parameters 1.2 times (1.5 times for terminal

methyl groups) of that of the preceding carbon atom. Two CH_2Cl_2 molecules, both [PF6]– anions, and both CF_3 groups exhibit disorder and were refined accordingly. CCDC 2346496 contains the supplementary crystallographic data for this paper. These data can be obtained free of charge from The Cambridge Crystallographic Data Centre via www.ccdc.cam.ac.uk/structures.

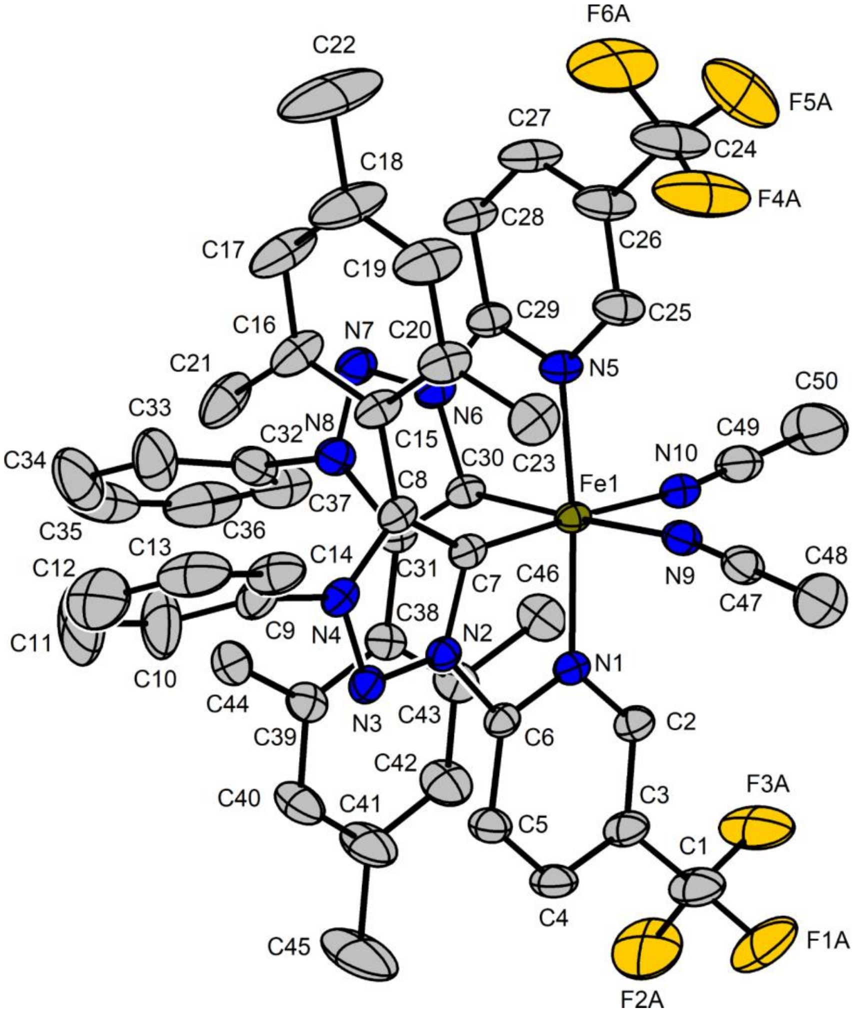

Figure S75. Crystal structure of product *rac*-**Fe2**. Only one enantiomer is shown. The $[PF_6]^–$ anions, solvent molecules, and the hydrogen atoms are not shown. Disorder is not shown. Displacement ellipsoids are shown for clarity at 50 % probability level at 100 K.

Table S4. Selected crystallographic data and details of the structure determination for $C_{50}H_{44}F_6FeN_{10}\cdot$ 2 PF_6 · 3 CH_2Cl_2.

Identification code	iND092
Empirical formula	$C_{53}H_{50}Cl_6F_{18}FeN_{10}P_2$
Molar mass / g·mol^{-1}	1499.52
Space group (No.)	$P\bar{1}$ (2)
a / Å	12.2526(5)
b / Å	13.4256(4)
c / Å	20.2744(8)
α / °	84.7870(10)
β / °	80.5560(10)
γ / °	76.5040(10)
V / Å^3	3194.3(2)
Z	2
$\rho_{calc.}$ / g·cm^{-3}	1.559
μ / mm^{-1}	0.635
Color	dark red
Crystal habitus	plate
Crystal size / mm^3	0.532 x 0.242 x 0.086
T / K	100
λ / Å	0.71073 (Mo-K$_\alpha$)
θ range / °	2.040 to 29.670
Range of Miller indices	$-16 \leq h \leq 17$
	$-18 \leq k \leq 18$
	$-28 \leq l \leq 28$
Absorption correction	multi-scan and numerical
T_{min}, T_{max}	0.8075, 0.9469
R_{int}, R_σ	0.0323, 0.0230
Completeness of the data set	0.999
No. of measured reflections	125711
No. of independent reflections	17919
No. of parameters	1016
No. of restraints	883
S (all data)	1.246
$R(F)$ ($I \geq 2\sigma(I)$, all data)	0.0606, 0.0682
$wR(F^2)$ ($I \geq 2\sigma(I)$, all data)	0.1488, 0.1539
Extinction coefficient	not refined
$\Delta\rho_{max}$, $\Delta\rho_{min}$ / e·Å^{-3}	1.153, –0.993

Λ-**Fepin2**:

A suitable crystal of $C_{52}H_{60}FeN_8(PF_6)_2$ was selected under inert oil and mounted using a MiTeGen loop. Intensity data of the crystal were recorded with a D8 Quest diffractometer (Bruker AXS). The instrument was operated with Mo-Kα radiation (0.71073 Å, microfocus source) and equipped with a PHOTON III C14 detector. Evaluation, integration and reduction of the diffraction data was carried out using the Bruker APEX 3 software suite.[20] Multi-scan and numerical absorption corrections were applied using the SADABS program.[21,22] The structure was solved using dual-space methods (SHELXT-2018/2) and refined against F2 (SHELXL-2019/1 using ShelXle interface).[23–25] All non-hydrogen atoms were refined with anisotropic displacement parameters. The hydrogen atoms were refined using the "riding model" approach with isotropic displacement parameters 1.2 times (1.5 times for terminal methyl groups) of that of the preceding carbon atom. One $[PF_6]^–$ anion and one acetonitrile ligand were disordered and were refined accordingly using the DSR plugin[26] implemented in ShelXle. The residual electron density in the solvent accessible voids likely belongs to an isopropanol solvent molecule. However, no plausible model could be obtained without using very hard restraints, so it was eliminated using the SQUEEZE algorithm in the PLATON software.[27,28] CCDC 2346497 contains the supplementary crystallographic data for this paper. These data can be obtained free of charge from The Cambridge Crystallographic Data Centre via www.ccdc.cam.ac.uk/structures.

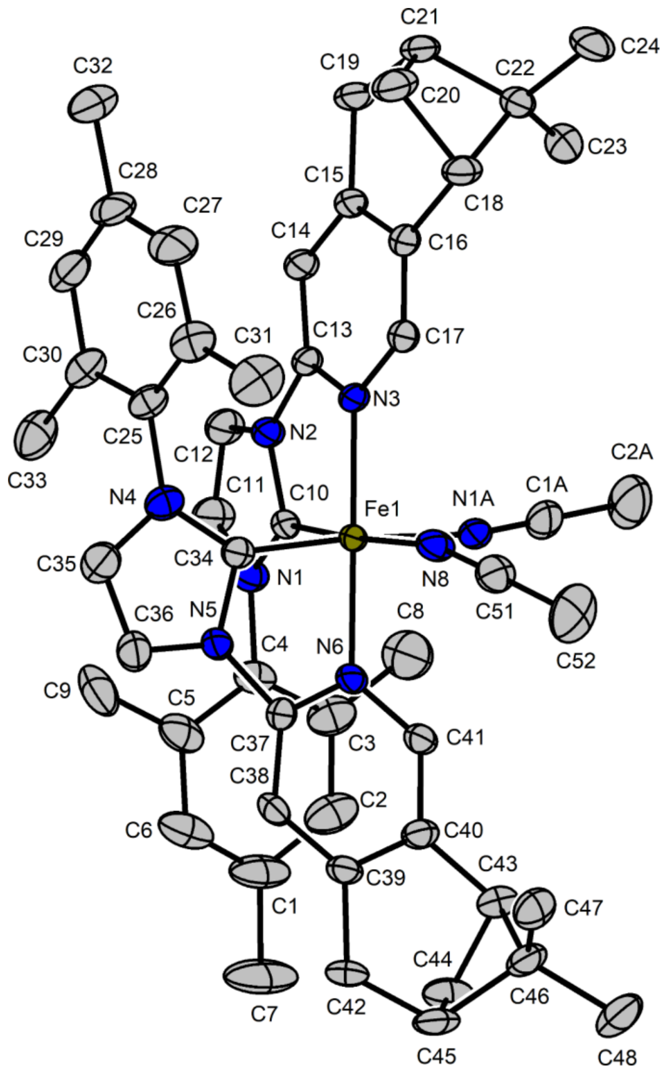

Figure S76. Crystal structure of product Λ-**Fepin2**. Only one enantiomer is shown. The $[PF_6]^-$ anions, solvent molecules, and the hydrogen atoms are not shown. Disorder is not shown. Displacement ellipsoids are shown for clarity at 50 % probability level at 100 K.

Table S5. Selected crystallographic data and details of the structure determination for $C_{52}H_{60}FeN_8(PF_6)_2$.

Identification code	MVN021K
Empirical formula	$C_{52}H_{60}F_{12}FeN_8P_2$
Molar mass / g·mol^{-1}	1142.87
Space group (No.)	*P*3$_2$ (145)
a / Å	11.2151(7)
c / Å	39.789(4)
V / Å^3	4334.1(7)

Z	3
$\rho_{calc.}$ / g·cm^{-3}	1.314
μ / mm^{-1}	0.396
Color	orange
Crystal habitus	plate
Crystal size / mm^3	0.377 x 0.152 x 0.132
T / K	100
λ / Å	0.71073 (Mo-K$_\alpha$)
θ range / °	2.599 to 28.280
Range of Miller indices	$-14 \leq h \leq 14$
	$-13 \leq k \leq 14$
	$-52 \leq l \leq 53$
Absorption correction	multi-scan and numerical
T_{min}, T_{max}	0.8485, 1.0000
R_{int}, R_σ	0.0370, 0.0302
Completeness of the data set	0.998
No. of measured reflections	70144
No. of independent reflections	14246
No. of parameters	781
No. of restraints	490
S (all data)	1.106
$R(F)$ ($I \geq 2\sigma(I)$, all data)	0.0484, 0.0495
$wR(F^2)$ ($I \geq 2\sigma(I)$, all data)	0.1274, 0.1283
Extinction coefficient	not refined
Flack parameter x	0.019(5)
$\Delta\rho_{max}$, $\Delta\rho_{min}$ / e·Å^{-3}	0.808, –0.354

15. References

[1] S. Roy, H. Khatua, S. K. Das and B. Chattopadhyay, *Angew. Chem. Int. Ed.*, 2019, **58**, 11439-11443.

[2] L. I. Dixon, M. A. Carroll, T. J. Gregson, G. J. Ellames, R. W. Harrington and W. Clegg, *Eur. J. Org. Chem.*, 2013, **2013**, 2334-2345.

[3] A. Bolje, D. Urankar and J. Košmrlj, *Eur. J. Org. Chem.*, 2014, **2014**, 8167-8181.

[4] A. Bolje and J. Košmrlj, *Org. Lett.*, 2013, **15**, 5084-5087.

[5] M. Virant and J. Košmrlj, *J. Org. Chem.*, 2019, **84**, 14030-14044.

[6] L. Liang, Y. Sun, R. Duan, R. Yang, L. Qu, K. Zhang and Z. Li, *Anal. Chem.*, 2021, **93**, 12434-12440.

[7] X. Sala, A. M. Rodríguez, M. Rodríguez, I. Romero, T. Parella, A. von Zelewsky, A. Llobet and J. Benet-Buchholz, *J. Org. Chem.*, 2006, **71**, 9283-9290.

[8] J. Wu, J. Zhou, Y. Shi and J. Zhu, *Synth. Commun.*, 2016, **46**, 1619-1624.

[9] A. V. Malkov, I. R. Baxendale, M. Bella, V. Langer, J. Fawcett, D. R. Russell, D. J. Mansfield, M. Valko and P. Kočovský, *Organometallics*, 2001, **20**, 673-690.

[10] S. Das, A. W. Ehlers, S. Patra, B. de Bruin and B. Chattopadhyay, *J. Am. Chem. Soc.*, 2023, **145**, 14599-14607.

[11] For our recent work focussing on chiral-at-iron complexes, see: (a) Y. Hong, L. Jarrige, K. Harms and E. Meggers, *J. Am. Chem. Soc.*, 2019, **141**, 4569-4572. (b) Y. Hong, T. Cui, S. Ivlev, X. Xie and E. Meggers, *Chem. Eur. J.*, 2021, **27**, 8557-8563. (c) N. Demirel, J. Haber, S. I. Ivlev and E. Meggers, *Organometallics*, 2022, **41**, 3852-3860.

[12] (a) A. Liske, K. Verlinden, H. Buhl, K. Schaper and C. Ganter, *Organometallics*, 2013, **32**, 5269-5272. (b) K. Verlinden, H. Buhl, W. Frank and C. Ganter, *Eur. J. Inorg. Chem.*, 2015,

2015, 2416-2425. (c) S. V. C. Vummaleti, D. J. Nelson, A. Poater, A. Gomez-Suarez, D. B. Cordes, A. M. Z. Slawin, S. P. Nolan and L. Cavallo, *Chem. Sci.*, 2015, **6**, 1895-1904.

[13] (a) H. V. Huynh, *Chem. Rev.* 2018, **118**, 9457-9492. (b) D. Munz, *Organometallics*, 2018, **37**, 275-289. (c) J. Beerhues, H. Aberhan, T.-N. Streit and B. Sarkar, *Organometallics*, 2020, **39**, 4557-4564. (d) J. Zhang, Md. M. Rahman, Q. Zhao, J. Feliciano, E. Bisz, B. Dziuk, R. Lalancette, R. Szostak and M. Szostak, *Organometallics*, 2022, **41**, 1806-1815.

[14] G. Meng, L. Kakalis, S. P. Nolan and M. Szostak, *Tetrahedron Lett.*, 2019, **60**, 378-381.

[15] (a) L. Falivene, Z. Cao, A. Petta, L. Serra, A. Poater, R. Oliva, V. Scarano and L. Cavallo, *Nat. Chem.*, 2019, **11**, 872-879. (b) A. Poater, F. Ragone, R. Mariz, R. Dorta and L. Cavallo, *Chem. Eur. J.*, 2010, **16**, 14348-14353. (c) A. Poater, F. Ragone, S. Giudice, C. Costabile, R. Dorta, S. P. Nolan and L. Cavallo, *Organometallics*, 2008, **27**, 2679-2681.

[16] G. Antinucci, B. Dereli, A. Vittoria, P. H. M. Budzelaar, R. Cipullo, G. P. Goryunov, P. S. Kulyabin, D. V. Uborsky, L. Cavallo, C. Ehm, A. Z. Voskoboynikov and V. Busico, *ACS Catal.*, 2022, **12**, 6934-6945.

[17] C. Barnett, M. L. Cole and J. B. Harper, *ACS Omega*, 2022, **7**, 34657-34664.

[18] Z. Zhou, Y. Tan, T. Yamahira, S. Ivlev, X. Xie, R. Riedel, M. Hemming, M. Kimura and E. Meggers, *Chem*, 2020, **6**, 2024-2034

[19] Y. Liu, X. Guan, E. L.-M. Wong, P. Liu, J.-S. Huang and C.-M. Che, *J. Am. Chem. Soc.*, 2013, **135**, 7194-7204.

[20] *APEX3*, Bruker AXS Inc., Madison, Wisconsin, USA, 2018.

[21] *SADABS*, Bruker AXS Inc., Madison, Wisconsin, USA, 2016.

[22] L. Krause, R. Herbst-Irmer, G. M. Sheldrick and D. Stalke, *J. Appl. Crystallogr.*, 2015, **48**, 3-10.

[23] G. M. Sheldrick, *Acta Crystallogr., Sect. A: Found. Adv.*, 2015, **71**, 3-8.

[24] G. M. Sheldrick, *Acta Crystallogr., Sect. C: Struct. Chem.*, 2015, **71**, 3-8.

[25] C. B. Hübschle, G. M. Sheldrick and B. Dittrich, *J. Appl. Crystallogr.*, 2011, **44**, 1281-1284.

[26] D. Kratzert and I. Krossing, *J. Appl. Crystallogr.*, 2018, **51**, 928-934.

[27] A. L. Spek, *Acta Crystallogr., Sect. C: Struct. Chem.*, 2015, **71**, 9-18.

[28] A. L. Spek, *PLATON - A Multipurpose Crystallographic Tool*, Utrecht University, Utrecht, The Netherlands, 2019.
